# Terminal differentiation and persistence of effector regulatory T cells essential for preventing intestinal inflammation

**DOI:** 10.1038/s41590-024-02075-6

**Published:** 2025-02-04

**Authors:** Stanislav Dikiy, Aazam P. Ghelani, Andrew G. Levine, Stephen Martis, Paolo Giovanelli, Zhong-Min Wang, Giorgi Beroshvili, Yuri Pritykin, Chirag Krishna, Xiao Huang, Ariella Glasner, Benjamin D. Greenbaum, Christina S. Leslie, Alexander Y. Rudensky

**Affiliations:** 1 Howard Hughes Medical Institute and Immunology Program, Ludwig Center at Memorial Sloan Kettering Cancer Center, New York, NY USA; 2 Immunology and Microbial Pathogenesis Program, Weill Cornell Graduate School of Medical Sciences, New York, NY USA; 3 Computational Oncology, Department of Epidemiology & Biostatistics, Memorial Sloan Kettering Cancer Center, New York, NY USA; 4 Gerstner Sloan Kettering Graduate School of Biomedical Sciences, Memorial Sloan Kettering Cancer Center, New York, NY USA; 5 Computational and Systems Biology Program, Memorial Sloan Kettering Cancer Center, New York, NY USA; 6 Lewis-Sigler Institute for Integrative Genomics and Department of Computer Science, Princeton University, Princeton, NJ USA; 7 Present Address: Department of Immunology and Microbiology, Scripps Research, La Jolla, CA USA; 8 Present Address: Department of Laboratory Medicine, University of California, San Francisco, San Francisco, CA USA

**Keywords:** Chronic inflammation, Autoimmune diseases, Regulatory T cells

## Abstract

Regulatory T (T_reg_) cells are a specialized CD4^+^ T cell lineage with essential anti-inflammatory functions. Analysis of T_reg_ cell adaptations to non-lymphoid tissues that enable their specialized immunosuppressive and tissue-supportive functions raises questions about the underlying mechanisms of these adaptations and whether they represent stable differentiation or reversible activation states. Here, we characterize distinct colonic effector T_reg_ cell transcriptional programs. Attenuated T cell receptor (TCR) signaling and acquisition of substantial TCR-independent functionality seems to facilitate the terminal differentiation of a population of colonic effector T_reg_ cells that are distinguished by stable expression of the immunomodulatory cytokine IL-10. Functional studies show that this subset of effector T_reg_ cells, but not their expression of IL-10, is indispensable for colonic health. These findings identify core features of the terminal differentiation of effector T_reg_ cells in non-lymphoid tissues and their function.

## Abstract (web summary)

The authors show that terminally differentiated colonic T_reg_ cells are required for maintaining colonic health and, although these cells are major producers of this cytokine, IL-10 is dispensable for their suppressive function.

## Main

T_reg_ cells are a specialized suppressive T cell lineage defined by the transcription factor (TF) Foxp3 (refs. ^1,2^). T_reg_ cells are essential for the prevention and control of autoimmunity, as their congenital deficiency or experimental depletion results in fulminant and fatal systemic autoimmune and inflammatory disease^3–9^. Studies have uncovered additional essential adaptive as well as maladaptive functions of T_reg_ cells in healthy tissue, during inflammation and in cancer in addition to immune regulation^10–22^.

T_reg_ cell generation from thymocytes and naive CD4^+^ T cells in the thymus and periphery, respectively, has been well characterized^1^. However, the activation and differentiation of mature T_reg_ cells in secondary lymphoid organs (SLOs) and non-lymphoid tissues, which results in the diversification of T_reg_ cell states and function, has only recently been explored. T_reg_ cells in the periphery have been described as having ‘resting’ versus ‘activated’ or ‘effector’ phenotypes. The former share features with naive or central memory CD4^+^ T cells, whereas the latter have more potent suppressor activity, preferentially traffic to non-lymphoid tissue and have been shown to expand under inflammatory conditions^23,24^. Several recent studies have leveraged genomic techniques to identify TFs associated with the transition of T_reg_ cells within the SLOs to those in non-lymphoid tissues. One pair of studies suggests that stepwise expression of the TFs Nfil3, Batf and Gata3 facilitates the acquisition of ‘effector’ molecules and migration to non-lymphoid tissues in both mouse and human T_reg_ cells, whereas another study points to increased expression of RORα and Gata3 as drivers of a ‘non-lymphoid’ T_reg_ cell phenotype^25–27^.

However, it remains unclear whether mature T_reg_ cells are truly undergoing differentiation in the periphery into multiple effector states, akin to conventional CD4^+^ T cells. Alternatively, these cells may adopt distinct transient gene expression programs specified by ongoing environmental conditioning. In the latter scenario, T_reg_ cells, retaining plasticity, would return to a ‘resting’ state upon the withdrawal of these stimuli and potentially adopt a different state of activation in response to distinct stimuli. Previous studies have suggested that this occurs for T_reg_ cells under a variety of perturbations, including systemic autoimmunity, viral infection and within tumors^21,24^. However, recent work has identified T_reg_ cells that durably express the TF T-bet and specifically control type 1 immune responses^28^. Importantly, these cells were inefficient at controlling other major types of immune responses, suggesting a specialized function with diminished plasticity and therefore terminal differentiation of this population.

A caveat of many comparative studies of ‘effector’ and ‘resting’ T_reg_ cells is that they contrast T_reg_ cells present in SLOs with those in non-lymphoid organs or those within SLOs bearing markers suggesting preferential retention versus exit from that tissue^23,25,27^. As T cells in SLOs are overall more quiescent than those from non-lymphoid tissues, quiescence can be conflated with a less differentiated state in at least some of these studies^29^. Therefore, we wanted to study T_reg_ cell differentiation specifically and exclusively within a non-lymphoid tissue. Additionally, we wanted to identify a T_reg_ cell effector state using a validated functional molecule that is not functionally associated with lymph node egress. Using a fate-mapping approach tied to the expression of the cytokine interleukin (IL)-10, we found distinct, specialized populations of T_reg_ cells in the colon that were apparently terminally differentiated and acquired a specialized gene expression program at least partly through attenuation of TCR signaling. Specific depletion of this IL-10-expressing colonic T_reg_ cell subset revealed its requisite function in colonic health.

## Results

### Identification of a stable effector T_reg_ cell population

**Fig. 1:**
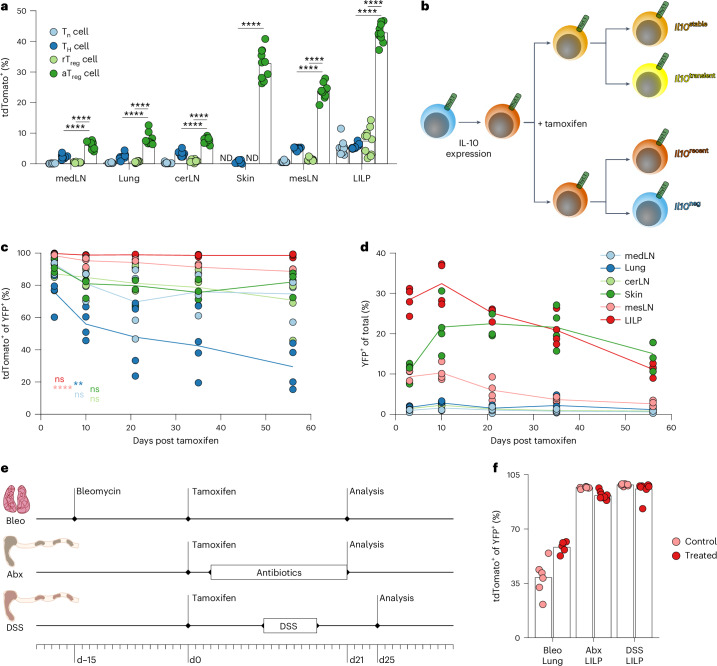
A population of colonic T_reg_ cells stably expresses IL-10. **a**, Frequencies of tdTomato^+^ cells among naive (T_n_) (CD44^lo^Thy1.1^–^) and helper (T_H_) cells (CD44^hi^Thy1.1^–^), resting (rT_reg_) (Thy1.1^+^CD62L^+^) and activated (aT_reg_) (Thy1.1^+^CD62L^–^) T_reg_ cells (all CD4^+^TCRβ^+^) isolated from tissues of 15-week-old *Il10*^FM^ mice. medLN, cerLN, and mesLN: mediastinal, cervical, and mesenteric lymph nodes, respectively. LILP: large intestine lamina propria; ND, not detected. Each point represents an individual mouse (*n* = 10) and data are representative of two independent experiments. Unpaired two-sided *t*-tests with Holm’s correction for multiple comparisons. **b**–**d,** *Il10*^FM^ mice (7–15 weeks old) were treated with tamoxifen and analyzed 3–56 days later. Mice analyzed together were littermates, treated at different times and analyzed on the same day. Schematic of possible cell states in IL-10 fate-mapping experiments (**b**). Frequencies of tdTomato^+^ cells among YFP^+^ (**c**) and YFP^+^ among all (**d**) T_reg_ cells (Thy1.1^+^CD4^+^TCRβ^+^) isolated from indicated tissues of mice at indicated days after tamoxifen treatment. Each point represents an individual mouse, lines indicate mean per tissue and data are pooled from two independent experiments (*n* = 4 mice per timepoint). In **c**, significance was determined by one-way ANOVA with Holm’s correction for multiple comparisons. **e**,**f**, Analysis of stability of IL-10 expression after perturbation in 7–10-week-old *Il10*^FM^ mice. For the bleomycin challenge, mice were first treated with bleomycin or vehicle, then 15 days later treated with tamoxifen and analyzed 21 days later (Bleo). For microbiota depletion, mice were treated with tamoxifen, 3 days later given antibiotics or control drinking water and analyzed 18 days later (Abx). For colitis induction, female mice were treated with tamoxifen, then 10 days later given drinking water containing 3% DSS or vehicle for 7 days and analyzed 8 days later (DSS). Experimental schematics (**e**) and frequencies of tdTomato^+^ cells among YFP^+^ T_reg_ cells isolated from the lungs (Bleo) or LILP (Abx, DSS) of challenged and littermate control mice (**f**). Each point represents an individual mouse (*n* = 6 per group for Bleo; 8–9 per group for Abx and DSS) and data are pooled from two independent experiments. ns, *P* > 0.05; **P < 0.01, ****P < 0.0001. Source data

**Extended Data Fig. 1:**
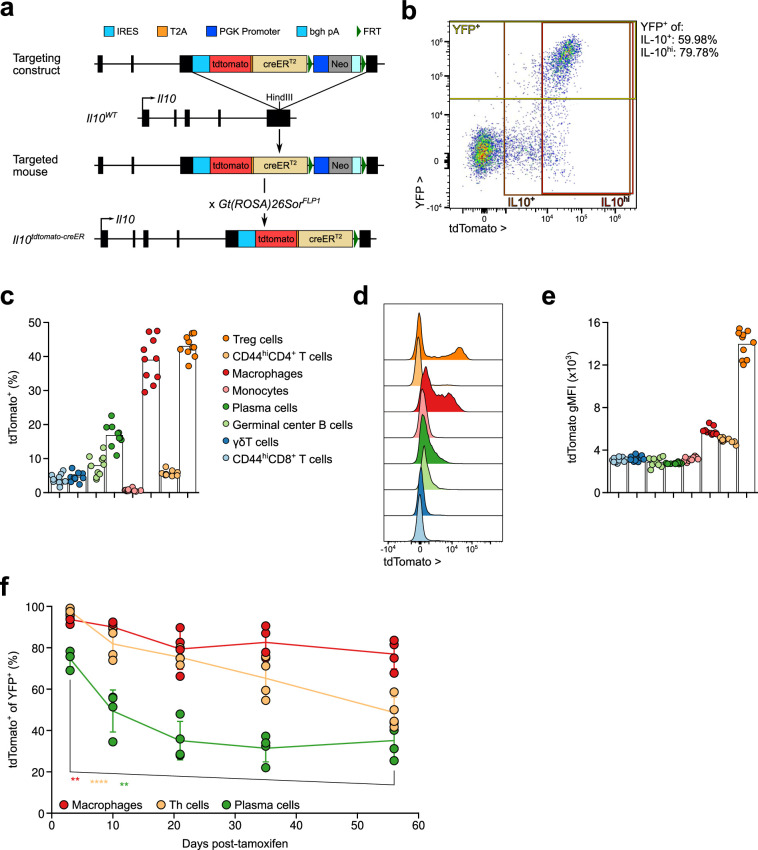
Characterization of *Il10*^*FM*^ mice. **a**. Strategy for generating *Il10*^*tdTomato-CreER*^ mice. Insertion of an IRES-tdTomato-T2A-CreER cassette into the 3’ untranslated region of the *Il10* gene allows for co-expression of a fluorescent reporter and inducible recombinase with endogenous *Il10* expression. See Methods for details. **b**. 15-week-old *Il10*^*FM*^ mice were treated with tamoxifen and analyzed 3 later. 2D flow plots of YFP^+^ cells among tdTomato^+^ Treg cells isolated from the colonic lamina propria. Representative frequencies of YFP^+^ among tdTomato^+^ or tdTomato^hi^ cells are shown. **c**-**e**. Frequencies (**c**) and representative histograms (**d**) of tdTomato^+^ cells and their level of tdTomato expression (geometric mean fluorescent intensity, gMFI) (**e**) among different immune cells subsets isolated from the colonic lamina propria of 15-week-old *Il10*^*FM*^ mice. See Methods for cell gating strategies. (**c**, **e**) Each point represents an individual mouse (n = 10) and data are representative of two independent experiments. (**d**) Histograms are from a single mouse, representative of 20 mice from two independent experiments. **f**. 7- to 15-week-old *Il10*^*FM*^ mice were treated with tamoxifen and analyzed 3 to 56 days later. Mice analyzed together were littermates, treated at different times, andanalyzed on the same day. Frequencies of tdTomato^+^ cells among YFP^+^ macrophages, plasma cells, and Th cells isolated from the colonic lamina propria of mice at indicated days after tamoxifen treatment. Each point represents an individual mouse, lines indicate mean per tissue, and data are pooled from two independent experiments (n = 4 mice per time point). One-way ANOVA with Holm’s correction for multiple comparisons. *P*-value < 0.01 **, < 0.0001 ****. Source data

To track the fate of T_reg_ cells after activation, we generated a mouse model allowing the identification and labeling of effector T_reg_ cells. As their distinguishing feature, we chose the immunomodulatory cytokine IL-10, as T_reg_ cells have been shown to produce this cytokine to modulate immune responses in diverse settings^30–32^. We generated an IL-10 reporter allele by inserting the coding sequence for a tdTomato fluorescent reporter fused with a tamoxifen-inducible Cre recombinase into the 3′ untranslated region (UTR) of the *Il10* gene (Extended Data Fig. 1a). In *Il10*^tdTomato-CreER^ mice, cells expressing IL-10 were detectable because of tdTomato expression and harbored a tamoxifen-inducible Cre recombinase. To track T_reg_ cells, we crossed these mice to *Foxp3*^Thy1.1^ mice^33^. We observed increased IL-10 expression by T_reg_ cells versus conventional activated or naive CD4^+^ T cells as well as by activated (CD44^hi^CD62L^–^) versus resting (CD44^lo^CD62L^+^) T_reg_ cells across lymphoid and non-lymphoid tissues (Fig. 1a). Importantly, high frequencies of tdTomato (IL-10)^+^ colonic macrophages and plasma cells, known IL-10^+^ populations, as well as differential intensity of tdTomato fluorescence across IL-10 expressing cell types, suggested that the *Il10*^tdTomato-CreER^ allele allows for sensitive and faithful reporting of endogenous IL-10 expression (Extended Data Fig. 1c–e).

To assess the stability of IL-10 expression in T_reg_ cells, we introduced a *Gt(ROSA)26Sor*^*LSL-YFP*^ recombination reporter into *Il10*^tdTomato-CreER^*Foxp3*^Thy1.1^ mice (referred to as *Il10*^FM^ mice). This enabled fate-mapping through activation of the Cre recombinase in IL-10^+^ cells by tamoxifen administration, inducing their irreversible marking by YFP expression^34^. Using *Il10*^FM^ mice, we sought to identify T_reg_ cells that were expressing IL-10 at the time of tamoxifen treatment and maintained (*Il10*^stable^) or lost IL-10 expression (*Il10*^transient^), acquired IL-10 expression after tamoxifen administration (*Il10*^recent^) or were not expressing IL-10 at the time of tamoxifen treatment or analysis (*Il10*^neg^; Fig. 1b). Analysis of YFP and tdTomato expression shortly after tamoxifen administration revealed efficient labeling of IL-10^+^ T_reg_ cells in the colon, particularly for cells with high IL-10 expression (Extended Data Fig. 1b). Analysis at different timepoints after tamoxifen administration revealed that all IL-10^+^ T_reg_ cells in the colon stably maintained IL-10 expression; that is, all tagged YFP^+^ cells continued to be tdTomato^+^, over at least 8 weeks (Fig. 1c). This contrasted with cells from other tissues and from the colon-draining mesenteric lymph node (mesLN), where variable proportions of YFP^+^ cells lost IL-10 expression (Fig. 1c). Even though they underwent gradual turnover, colonic *Il10*^stable^ T_reg_ cells were long-lived, with a population of YFP^+^ cells persisting at least 8 weeks after tagging (Fig. 1d). Analysis of other major IL-10^+^ cell types in the colon suggested that this stability was not an intrinsic feature of IL-10 expression in that tissue, as fate-mapped YFP^+^ plasma cells, macrophages and conventional T cells were prone to lose IL-10 expression over time (Extended Data Fig. 1f).

The stability and persistence of IL-10^+^ colonic T_reg_ cells suggested that they might represent a specialized, terminally differentiated T_reg_ cell population. However, T_reg_ cells in the colon may stably maintain IL-10 expression simply because the intestinal microbiota is a source of constant stimulation for colonic immune cells; a variable absent in ‘microbe-poor’ tissues such as the lung^35–37^. Thus, the unique phenotype of colonic T_reg_ cells could be explained by the fact that these cells, unlike those in the lung, are exposed to chronic stimuli and thus the apparent stability of IL-10 expression reflects ongoing, albeit reversible, activation of these cells rather than their terminal differentiation. To address this possibility, we induced chronic inflammation in the lungs of *Il10*^FM^ mice by intranasal bleomycin challenge. To assess the stability of IL-10 expression by T_reg_ cells in the presence of this inflammatory stimulation, *Il10*^FM^ mice were treated with tamoxifen 15 days after bleomycin challenge to ‘fate-map’ IL-10^+^ cells and were then analyzed for stability 21 days later (Fig. 1e). Although bleomycin challenge increased the pool of T_reg_ cells expressing IL-10, a proportion still lost IL-10 expression (Fig. 1f).

To directly test the role of the intestinal microbiota in maintaining the *Il10*^stable^ state, we administered tamoxifen to *Il10*^FM^ mice 3 days before treating them with broad-spectrum antibiotics (Fig. 1e). YFP^+^ T_reg_ cells in the colons of antibiotic-treated *Il10*^FM^ mice maintained stable IL-10 expression comparable to cells in littermate controls receiving vehicle (Fig. 1f). These results suggest that *Il10*^stable^ cells, once differentiated, largely maintain their phenotypic state even if the intestinal microbiota is depleted. Finally, we examined whether IL-10^+^ T_reg_ cells would maintain IL-10 expression during a severe inflammatory insult. To test this idea, we first labeled pre-existing IL-10^+^ cells by administering tamoxifen to *Il10*^FM^ mice. Then, 10 days later, we induced colitis in these mice by providing dextran sulfate sodium (DSS) in the drinking water for 7 days and analyzed cells in the colon 8 days after DSS withdrawal (Fig. 1e). Despite inflammation and T_reg_ cell expansion, pre-existing YFP^+^ T_reg_ cells retained IL-10 expression. Together, these results suggest that rather than being a product of ongoing stimulation, *Il10*^stable^ T_reg_ cells in the colon represent a terminally differentiated cell state robust to perturbations.

### Colonic IL-10^+^ T_reg_ cells appear terminally differentiated

**Fig. 2:**
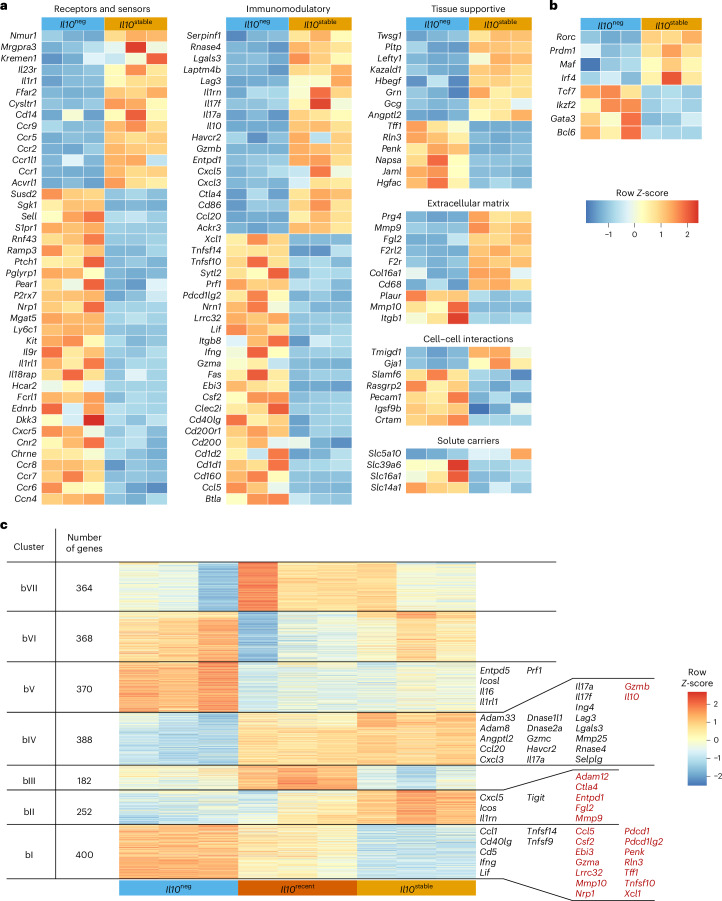
Distinct transcriptional features of *Il10*^neg^ and *Il10*^stable^ cells. RNA was isolated from *Il10*^neg^, *Il10*^recent^ and *Il10*^stable^ T_reg_ cells, as defined in Fig. 1b, sorted from the colonic lamina propria of 10-week-old *Il10*^FM^ mice treated 21 days earlier with tamoxifen and sequenced. **a**,**b**, Coloring depicts *Z*-score normalized log_2_-transformed gene FPKM counts for individual *Il10*^neg^ and *Il10*^stable^ samples. Genes shown are all significantly differentially expressed between *Il10*^neg^ and *Il10*^stable^ (log_2_FC > 1 and adjusted *P* < 0.05), annotated as encoding cell surface or secreted proteins and manually categorized (**a**) or select differentially expressed (adjusted *P* < 0.05) TFs (**b**). **c**, *K*-means clustering was performed on *Z*-score-normalized log_2_-transformed gene FPKM counts for genes significantly differentially expressed in any pairwise comparison (*P* < 0.05). Genes of interest differentially expressed between *Il10*^stable^ versus *Il10*^neg^ (black) or *Il10*^stable^ versus *Il10*^neg^ and *Il10*^recent^ (red) within clusters I, II, IV and V are indicated. See Methods for details. Negative binomial fitting with two-sided Wald’s significance test and the Benjamini–Hochberg correction for multiple comparisons. Significance testing and correction were performed on all genes. Source data

To elucidate the distinguishing features of stable IL-10^+^ colonic T_reg_ cells, we performed RNA-sequencing (RNA-seq) analysis on *Il10*^neg^, *Il10*^recent^ and *Il10*^stable^ T_reg_ cells isolated from the colonic lamina propria of *Il10*^FM^ mice 21 days after labeling. Owing to selective labeling of *Il10*^stable^ cells expressing high amounts of IL-10 (Extended Data Fig. 1b), we were careful to isolate *Il10*^recent^ cells with matched expression. Contrasting *Il10*^neg^ and *Il10*^stable^ cells revealed differential expression of immunomodulatory and tissue-supportive mediators, suggesting that these T_reg_ cell populations participate in distinct regulatory and physiological processes (Fig. 2a). This observation, combined with differential expression of genes encoding receptors for cytokines, chemokines, cell–cell interactions and the extracellular matrix overall suggested distinct specialized niches for the generation, residence and function of *Il10*^neg^ and *Il10*^stable^ cell populations (Fig. 2a). Indeed, based on differential TF expression, these populations seemed to represent the previously described Helios^hi^ and RORγt^hi^ colonic T_reg_ cells of thymic and extrathymic origin, respectively (Fig. 2b), which have been shown to expand in response to distinct cues and to contribute to the control of distinct immune responses^38–41^.

**Fig. 3:**
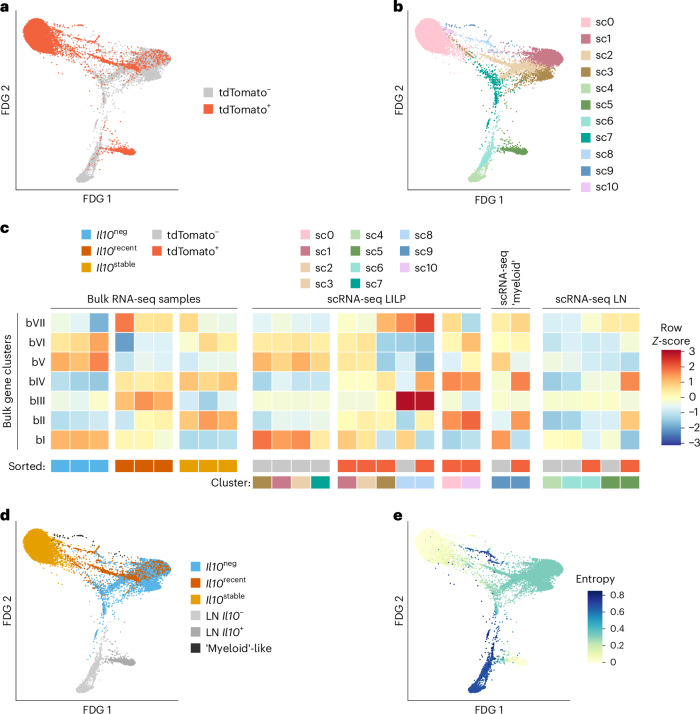
RNA-seq analysis of colonic T_reg_ cells indicating terminal differentiation of *Il10*^stable^ cells. tdTomato^+^ and tdTomato^–^ T_reg_ cells (Thy1.1^+^CD4^+^TCRβ^+^) were separately sorted from the colon (LILP) and mesLN of *Il10*^FM^ mice and processed for scRNA-seq. See Methods for details. Bulk RNA-seq data is from the analysis presented in Fig. 2. **a**,**b**, Two-dimensional force-directed graph layouts of tdTomato^+^ and tdTomato^–^ (**a**) T_reg_ cells from the LILP and lymph node or colored according to cluster (**b**). **c**, Integration of bulk and single-cell RNA-sequencing. Mean log_2_-transformed FPKM counts were computed for each *k*-means gene cluster for each bulk RNA-seq sample, and mean expression was then *Z*-score-normalized across samples per cluster. The scRNA-seq cells were scored for expression of bulk RNA-seq gene clusters, and the 11 nearest-neighbor cell clusters were then separated as originating from the tdTomato^+^ or tdTomato^–^ sample and manually organized. Per-subset expression score was *Z*-normalized across cell clusters per gene cluster. For clarity, scRNA-seq populations with very few cells are not depicted. Colored boxes indicate bulk RNA-seq samples sorted as *Il10*^neg^, *Il10*^recent^ or *Il10*^stable^ T_reg_ cell populations, and scRNA-seq populations by cluster and cell sample origin (tdTomato^+^ or tdTomato^–^). See Methods for details. **d**,**e**, Plots depicting two-dimensional force-directed graph layout for all tdTomato^+^ and tdTomato^–^ LILP and lymph node scRNA-seq cells. Coloring indicates manually determined similarity of gene expression to the bulk-sorted *l10*^neg^ (blue), *Il10*^recent^ (orange) and *Il10*^stable^ (yellow) T_reg_ cell populations or whether cells derive from the lymph node (light grays) or belong to a colonic ‘myeloid-like’ T cell population (dark gray) (**d**). Shading (color bar) indicates entropy, as determined by the Palantir algorithm (**e**). See Methods for details. Source data

**Fig. 4:**
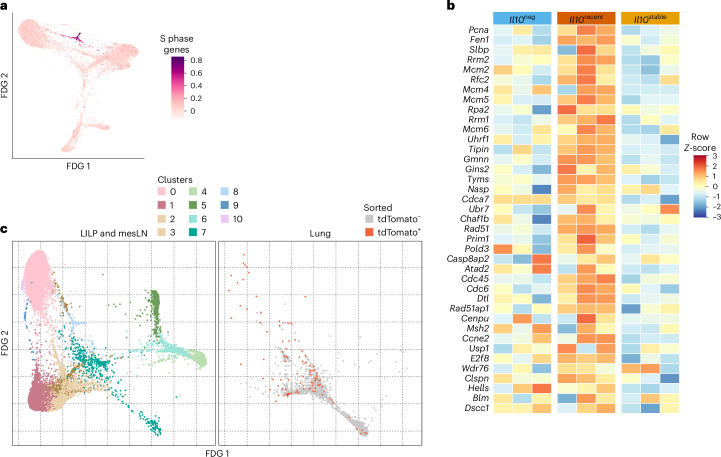
Characterization of IL-10^+^ and IL-10^−^ T_reg_ cells by scRNA-seq. **a**, tdTomato^+^ and tdTomato^–^ T_reg_ cells (Thy1.1^+^CD4^+^TCRβ^+^) were separately sorted from the colon (LILP) and mesLN of *Il10*^FM^ mice and scRNA-seq was performed; two-dimensional force-directed graph layout of all tdTomato^+^ and tdTomato^–^ LILP and lymph node scRNA-seq cells. Shading indicates gene expression score for genes associated with the S phase of the cell cycle. **b**, Bulk RNA-seq data from the analysis presented in Fig. 2. Heatmap showing log_2_-transformed, row Z-score-normalized FPKM counts for genes associated with the S phase of the cell cycle among bulk RNA-seq samples. **c**, tdTomato^+^ and tdTomato^–^ T_reg_ cells (Thy1.1^+^CD4^+^TCRβ^+^) were separately sorted from the colon lamina propria (LILP), mesLN and lung of *Il10*^FM^ mice and scRNA-seq was performed. Data are from scRNA-seq analysis presented in Fig. 3, with the addition of cells from the lung. two-dimensional force-directed graph layout of all tdTomato^+^ and tdTomato^–^ scRNA-seq cells. In the left panel, coloring indicates the assigned nearest-neighbor cluster for each cell when clustering LILP and lymph node cells, as in Fig. 3a; right panel coloring indicates cells sorted as tdTomato^+^ or tdTomato^–^ from the lung. Source data

**Extended Data Fig. 2:**
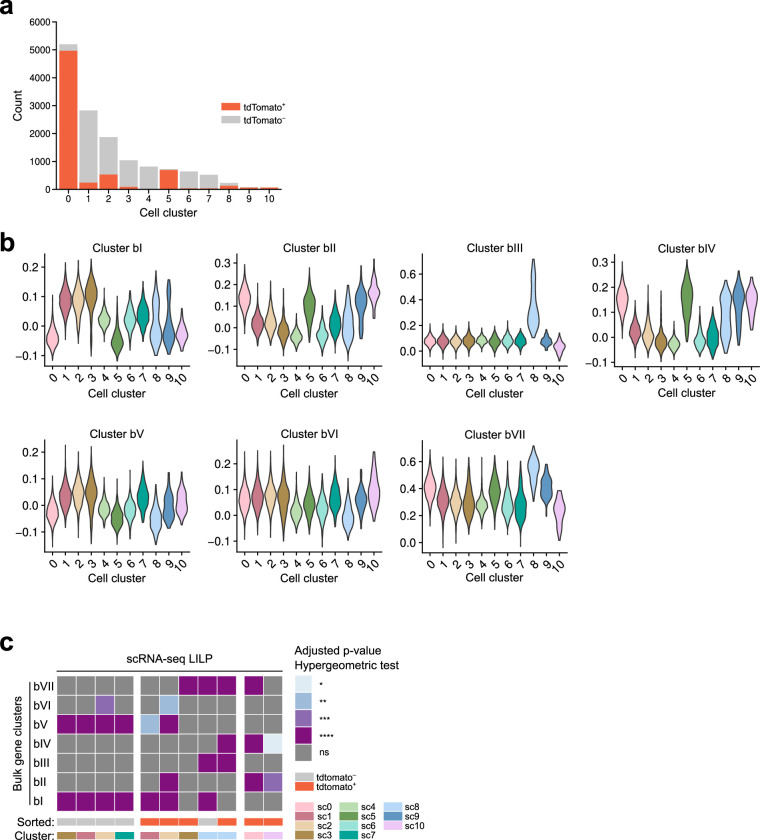
Expression of bulk RNA-seq gene clusters by scRNA-seq cell populations. Data are from scRNA-seq analysis presented in Fig. 3. **a**. Counts of cells originating from the tdTomato^+^ (red, IL-10^+^) or tdTomato^–^ (grey, IL-10^−^) samples assigned to each nearest neighbor cluster. **b**. The scRNA-seq cells were scored for expression of bulk RNA-seq gene clusters and violin plots depict per gene cluster score by each scRNA-seq cell cluster. See Methods for details. **c**. scRNA-seq cells were divided into subsets as in Fig. 3c, and statistical significance of enrichment of genes belonging to bulk RNA-seq k-means clusters bI-VII among genes with significantly higher expression in each scRNA-seq subset versus all other cells was determined. Shading indicates binned p-values of one-sided (upper tail) hypergeometric test for enrichment, with *p*-values adjusted for multiple comparisons using Holm’s method. See Methods for details. *P*-value > 0.05 ns, < 0.05 *, < 0.01 **, <0.001 ***. Source data

To gain insight into the differentiation of *Il10*^stable^ T_reg_ cells, we incorporated into our analysis the *Il10*^recent^ T_reg_ cell population—cells that gained IL-10 expression subsequent to tamoxifen treatment 21 days earlier—and performed single-cell RNA-seq (scRNA-seq) on colonic tdTomato^+^ and tdTomato^–^ T_reg_ cells (Figs. 2c, 3a–e, 4a–c and Extended Data Fig. 2a–c). Given that precursors of these tissue T_reg_ cells could be residing in the draining lymph nodes, we also analyzed the corresponding T_reg_ cells from the colonic mesLN^25,27^. First, we wished to reconcile our bulk RNA-seq and scRNA-seq analyses. We performed *k*-means clustering on the bulk RNA-seq data to identify gene clusters that distinguish *Il10*^neg^, *Il10*^recent^ and *Il10*^stable^ populations (Fig. 2c). This revealed two gene clusters: one including *Il10* itself, which increased in expression in *Il10*^stable^ versus both *Il10*^neg^ and *Il10*^recent^ cells (Fig. 2c, clusters bII and bIV). Conversely, many of the genes highly expressed in *Il10*^neg^ cells lost expression in *Il10*^stable^ versus *Il10*^recent^ cells (Fig. 2c, cluster bI). Next, we mapped expression of the bulk RNA-seq gene (bI–bVII) clusters to our scRNA-seq cell (sc0–sc10) clusters (Fig. 3c and Extended Data Fig. 2b,c). This analysis suggested that the *Il10*^stable^ cells were mostly present in sc0 and sc10, as these cell clusters had high expression of gene clusters bII and bIV (Fig. 3c and Extended Data Fig. 2b). Meanwhile, *Il10*^neg^ cells comprised cell clusters sc1, sc2, sc3 and sc7, as these had the highest expression of bulk gene clusters bI, bV and bVI (Fig. 3c and Extended Data Fig. 2b). Finally, *Il10*^recent^ cells were apparently present within cell cluster sc8, as these cells highly expressed genes from bulk clusters bIII and bVII but were also present among the tdTomato^+^ cells in clusters sc1, sc2 and sc3. These cells, and bulk-sequenced *Il10*^recent^ cells, both had intermediate expression of genes from bulk clusters bI and bII (Fig. 3c and Extended Data Fig. 2b). Altogether, this comparison of the scRNA-seq and bulk RNA-seq datasets pinpointed scRNA-seq cell subsets corresponding to the bulk-sorted, fate-mapped populations (Fig. 3d). Importantly, many of the bulk gene clusters showed statistically significant enrichment in the highly and differentially expressed genes of each scRNA-seq subset identified as cells representing the *Il10*^neg^, *Il10*^recent^ or *Il10*^stable^ bulk populations (Extended Data Fig. 2c).

To explore potential developmental relations between these cell clusters, we used the Palantir algorithm, which assigns entropy measures to cells, indicative of differentiation potential^42^. As a ‘starting cell’ for this analysis, we chose a random cell in cluster sc4, which was enriched for lymph node cells with markers of quiescence (*Lef1, Sell*). The relatively high entropy values in clusters sc1, sc2, sc3 and especially sc8 were consistent with the notion that these tdTomato^+^ cells might be differentiating into the *Il10*^stable^ (sc0) cells (Fig. 3e). Interestingly, the colonic high-entropy cluster (sc8) was enriched in cell-cycle-related genes, a feature also apparent in the bulk RNA-seq analysis of *Il10*^recent^ cells (Fig. 4a,b). Altogether, these analyses suggested that within the colon, *Il10*^recent^ cells underwent further differentiation into *Il10*^stable^ cells and that this process was associated with proliferation.

Our fate-mapping analysis of IL-10^+^ T_reg_ cells revealed *Il10*^transient^ cells in lungs (Fig. 1c). This suggested that in contrast to the colon, lung IL-10^+^ T_reg_ cells do not undergo similar terminal differentiation. This notion was supported by scRNA-seq analysis, as both tdTomato^+^ and tdTomato^–^ T_reg_ cells from the lung clustered separately from colonic T_reg_ cells with the lowest entropy values; that is, the most differentiated. Moreover, both lung T_reg_ cell populations tended to cluster together, suggesting overall similarity despite the difference in IL-10 expression (Fig. 4c). The presence of a small number of lung tdTomato^+^ cells clustering with colonic cell cluster sc0 might reflect a small population of terminally differentiated *Il10*^stable^ cells in the lung, either generated directly in the lung or migrant colonic *Il10*^stable^ T_reg_ cells, a possibility suggested by a recent study^43^.

### TCR-related TFs oppose colonic *Il10*^stable^ T_reg_ cell state

**Fig. 5:**
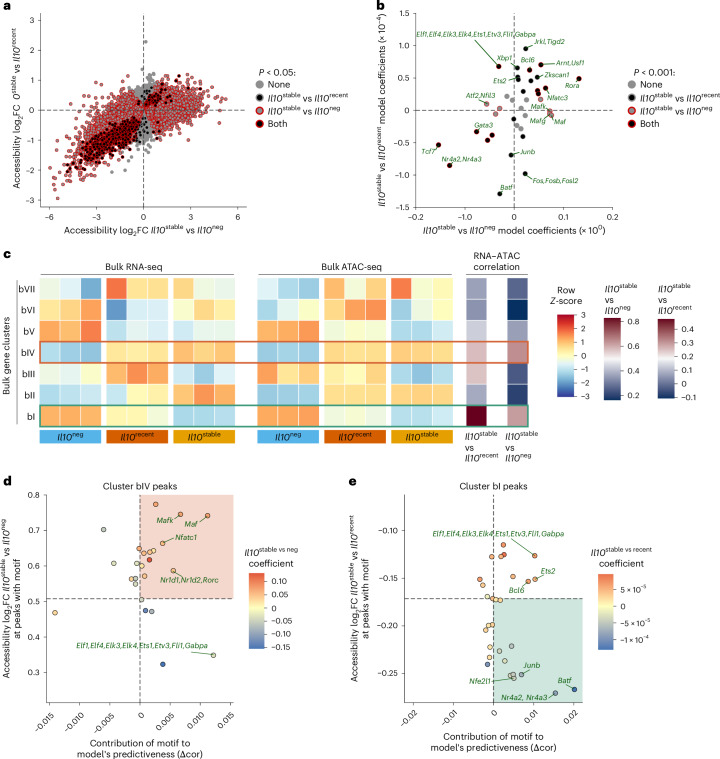
Characterization of colonic T_reg_ cell subsets by integrated RNA-seq and ATAC–seq analysis. *Il10*^neg^, *Il10*^recent^ and *Il10*^stable^ T_reg_ cells, as defined in Fig. 1b, were sorted from the LILP of 10-week-old *Il10*^FM^ mice treated 21 days earlier with tamoxifen and subjected to ATAC–seq analysis. Bulk RNA-seq data is from the analysis presented in Fig. 2. **a**, Plot showing mean log_2_FC peak accessibility for *Il10*^stable^ versus *Il10*^neg^ (*x* axis) and *Il10*^stable^ versus *Il10*^recent^ (*y* axis) samples. Coloring indicates peaks significantly differentially accessible (adjusted *P* < 0.05) in *Il10*^stable^ versus *Il10*^neg^ (red outline), *Il10*^stable^ versus *Il10*^recent^ (black fill), both (black fill: red outline) or neither (gray) comparisons. Negative binomial fitting with two-sided Wald’s significance test and the Benjamini–Hochberg correction for multiple comparisons. **b**, See Methods for model generation and coefficient determination. Plots showing per-motif coefficients for the svn (*y* axis) and svr (*x* axis) models. Coloring indicates motifs with significant (*P* < 0.001) coefficients: in neither (gray), svn (black fill), svr (red outline) or both (black fill, red outline) models. See Methods for modeling and significance testing details. **c**. Mean log_2_-transformed FPKM RNA or ATAC tag counts were computed for each *k*-means gene cluster for each bulk RNA-seq or ATAC–seq sample. Mean expression or accessibility was then Z-score normalized across samples per cluster. Shading indicates *Z*-score-normalized expression or accessibility count means. Pearson’s correlation coefficients were calculated for expression versus accessibility FC for the genes and associated peaks in each cluster. Shading indicates correlation coefficient. Clusters with the highest correlation for comparison (green, bI–svr; orange, bIV–svn) are boxed. **d**,**e,** See Methods for details. *x* axes, difference between original and motif withheld correlation coefficients (Δcor); *y* axes, log_2_FC peak accessibility for cluster bIV (**d**) and cluster bI (**e**) associated peaks containing each motif. Dashed line indicates log_2_FC peak accessibility for all cluster bIV (**d**) and cluster bI (**e**) associated peaks. Shading indicates coefficients for the svn (**d**) and svr (**e**) models. Highlighted quadrants with motifs positively contributing to the model’s predictiveness and associated with above-average increased (orange, **d**) or decreased (green, **e**) accessibility. Source data

Next, we sought to identify the transcriptional regulators and signaling pathways controlling the differentiation of *Il10*^stable^ colonic T_reg_ cells by performing assay for transposase-accessible chromatin with sequencing (ATAC–seq) on the same populations subjected to bulk RNA-seq analysis: *Il10*^stable^, *Il10*^recent^ and *Il10*^neg^ colonic T_reg_ cells isolated 21 days after tamoxifen treatment. Many of the differentially accessible peaks in the ATAC–seq atlas that distinguished *Il10*^stable^ from *Il10*^neg^ T_reg_ cells were also similarly differentially accessible between *Il10*^stable^ and *Il10*^recent^ populations (Pearson’s *r*, 0.602, *P* < 2.2 × 10^–16^), suggesting that identifying the pathways and TFs converging on these peaks might reveal which TFs differentially specify the *Il10*^stable^ versus *Il10*^neg^ state as well as TFs facilitating the differentiation of *Il10*^recent^ into *Il10*^stable^ cells (Fig. 5a).

To this end, we first identified motifs within each peak corresponding to TFs expressed in any of the three cell populations. We then fit separate linear ridge regressions on this peak-by-motif matrix to generate models that would predict accessibility differences at each peak in both the *Il10*^stable^ versus *Il10*^neg^ (svn model) and *Il10*^stable^ versus *Il10*^recent^ (svr model) comparisons^44–46^. Each unique motif is represented by a term in these models whose coefficient indicates the extent to which that motif contributes to predicting accessibility changes. Thus, large positive or negative coefficient values suggest that a motif is more strongly associated with increased or decreased accessibility at peaks harboring that motif, ultimately implying increased or decreased activity of the corresponding TFs at those loci. Comparing the coefficients from the two models revealed motifs associated with accessibility changes in a statistically significant manner in the svn model, the svr model or both (Fig. 5b). Coefficients for motifs for TFs differentially expressed between *Il10*^neg^ and *Il10*^stable^ cells, such as Gata3 and Maf, corresponded with their differential expression, suggesting biologically meaningful changes in TF activity revealed by this analysis (Figs. 2b and 5b). Notably, motifs of TFs that act downstream of TCR stimulation (AP-1 family members, Batf, Nr4a2/3) were associated with a loss of accessibility in *Il10*^stable^ versus *Il10*^recent^ and *Il10*^neg^ cells (Fig. 5b). This suggested that reduced TCR signaling was associated with the terminal differentiation of T_reg_ cells into the *Il10*^stable^ state.

We focused our subsequent analysis on the bulk RNA-seq gene clusters bI and bIV, as differential expression of those genes continued to increase (bIV) or decrease (bI) alongside *Il10* gene expression (Fig. 5c). Thus, expression of these genes was specifically gained or lost, respectively, during terminal differentiation of IL-10^+^ T_reg_ cells. A similar pattern was observed for differential chromatin accessibility of the peaks associated with these genes (Fig. 5c). Therefore, elucidation of motifs associated with chromatin accessibility changes at these peaks might reveal candidate TFs whose differential binding contributed to the regulation of gene expression at those loci. Furthermore, chromatin accessibility changes at these peaks had a stronger correlation with gene expression changes in both the *Il10*^stable^ versus *Il10*^neg^ and *Il10*^stable^ versus *Il10*^recent^ comparisons than in the overall ATAC–seq data (Fig. 5c).

**Extended Data Fig. 3:**
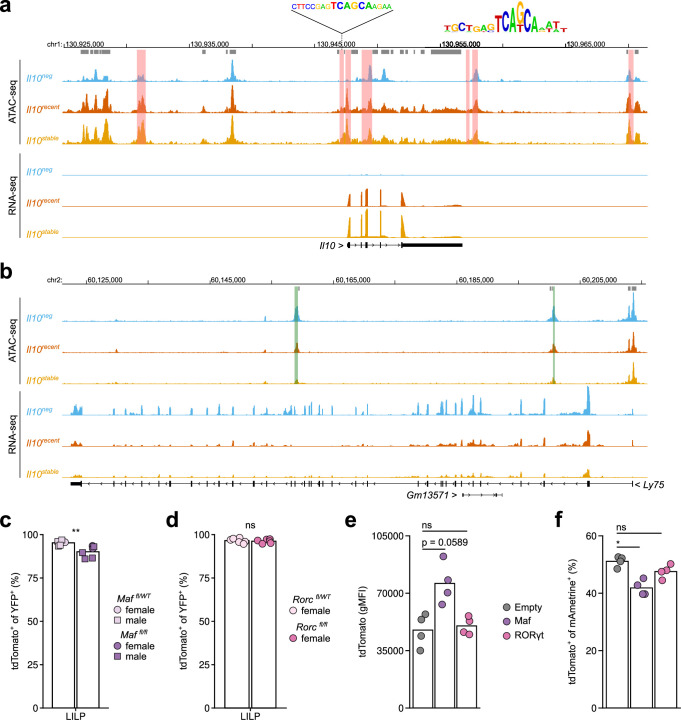
TFs regulating gene expression and phenotype of IL-10^+^ Treg cells. **a**, **b**. Data are from bulk RNA-seq analysis presented in Fig. 2. Representative tracks showing normalized pile-up of ATAC-seq and RNA-seq reads aligning to the *Il10* (**a**) and *Ly75* (**b**) gene loci. Coloring indicates cell states. Coordinates in a modified mm39 genome assembly are indicated. Grey bars indicate ATAC-seq peaks, orange bars indicate peaks containing at least one Maf motif, and green bars indicate peaks containing at least one Nr4a2/3 motif. Upper inset shows canonical Maf motif alongside the highest confidence identified Maf motif in the promoter region of *Il10*. **c**-**d**. 8- to 12-week-old male and female *Il10*^*tdTomato-CreER*^*Maf*^*FL/WT*^ or *Maf*^*FL/FL*^ (**a**) or *Il10*^*tdTomato-CreER*^*Rorc*^*FL/WT*^ or *Rorc*^*FL/FL*^ mice were treated with tamoxifen on days 0, 4, 11, 18, 25, and 32 and cells were isolated from the colonic lamina propria on day 35. Plots show frequencies of tdTomato^+^ cells among YFP^+^ Treg cells (Thy1.1^+^CD4^+^TCRβ^+^). Each point represents an individual mouse (n = 6 per group), and data are pooled from two (*Rorc*) or three (*Maf*) independent experiments. ANOVA for frequencies as a function of genotype and replicate, with *p*-value for the effect of genotype determined by Tukey’s ‘Honest Significant Difference’ method. **e**-**f**. IL-10^−^ Treg cells (Thy1.1^+^CD4^+^TCRβ^+^tdTomato^−^) were sorted from pooled secondary lymphoid organs, activated *in vitro*, and transduced with Cre-activated retroviral vectors expressing Maf or RORγt with mAmetrine fluorescence reporting on cDNA expression through an IRES. See Methods for details. Plots depict geometric mean of fluorescence intensity for tdTomato (**c**) or frequencies of tdTomato^+^ cells among Treg cells (**d**) three days after transduction. Each point represents cells obtained from an individual mouse (n = 4) and data are from a single experiment. *P*-value determined by paired, two-sided Student’s t-test for gMFI or frequency as a function of each over-expressing virus vs control, corrected for multiple comparisons using the Holm method. *P*-value > 0.05 ns, < 0.05 *. Source data

We wanted to use our models to elucidate the motifs that are associated with accessibility changes at gene clusters bI and bIV. We reasoned that comparing the performance of the models in predicting accessibility differences at specific peaks with individual motif terms removed might reveal the terms (motifs) that were specifically associated with altered accessibility at those peaks. For example, if removing motif *X* from the model decreased its performance (that is, reduced the correlation between predicted and actual accessibility fold change (FC) values) at peaks of cluster *Y*, this would suggest that TFs recognizing motif *X* are specifically active at cluster *Y* peaks. This analysis for cluster bIV peaks in the svn model associated Nfatc1, Rorc and Maf family motifs with increased accessibility in *Il10*^stable^ cells (Fig. 5d). For cluster bI peaks, the motifs of TCR-responsive TFs Nr4a2/3, Batf and AP-1 were associated with loss of accessibility in *Il10*^stable^ cells relative to less-differentiated *Il10*^recent^ cells (Fig. 5e). Reassuringly, peaks within each cluster harboring those motifs had greater magnitude accessibility differences than all peaks in the cluster (Fig. 5d,e, *y* axes), suggesting that the corresponding TFs contributed to the changes. Indeed, cluster bIV gene loci such as *Il10* itself showed peaks containing Maf motifs gaining accessibility alongside increased gene expression, whereas cluster bI loci such as *Ly75* showed decreased gene expression and accessibility at peaks containing Nr4a2/3 motifs (Extended Data Fig. 3a,b).

A hierarchy of Maf, RORγt and Blimp1 activities in IL-10 induction in colonic T_reg_ cells has been established^23,47–51^. Therefore, our epigenetic analysis seemed to confirm a role for at least two of these TFs in inducing IL-10 expression and suggested that they modulate the expression of co-regulated effector molecules. However, it has remained unknown whether these TFs are required to maintain IL-10 expression, let alone a larger gene expression program, in differentiated cells. Interestingly, our data comparing *Il10*^recent^ and *Il10*^stable^ T_reg_ cell populations did not indicate a discernable role for either Maf or RORγt in the latter after IL-10 induction, suggesting that these TFs might be dispensable for maintaining IL-10 expression and this T_reg_ cell state in general (Fig. 5b; note the lack of significant Δcor for Rorc or Maf motifs in Fig. 5e). We sought to test this possibility using both loss-of-function and gain-of-function strategies. First, we ablated conditional *Maf* and *Rorc* alleles in IL-10 expressing cells in *Il10*^tdTomato-CreER^*Maf*^fl^ and *Il10*^tdTomato-CreER^*Rorc*^fl^ mice by treating them with tamoxifen (Extended Data Fig. 3c,d). Induced loss of these TFs in T_reg_ cells, which had already acquired IL-10 expression, did not lead to substantial impairment of its maintenance, confirming the dispensability of these TFs for maintaining IL-10 expression (Extended Data Fig. 3c,d). Second, we inducibly expressed these TFs in in-vitro-activated T_reg_ cells using retroviral vectors. The enforced expression of Maf and RORγt did not increase the persistence of IL-10 expression, confirming that these TFs have a non-redundant role only in the induction of IL-10 expression by T_reg_ cells (Extended Data Fig. 3e,f). The higher per-cell expression of IL-10 in Maf over-expressing cells is consistent with the known role of Maf in directly activating the *Il10* gene, probably through a motif in the *Il10* promoter identified in our ATAC–seq analysis (Extended Data Fig. 3a,e). At the same time, the effect of enforced Maf expression in causing loss of IL-10 expression in a small proportion of cells may reflect a nuanced role for this TF in regulating IL-10 (Extended Data Fig. 3f).

### Diminished TCR dependence in long-lived *Il10*^stable^ cells

**Extended Data Fig. 4:**
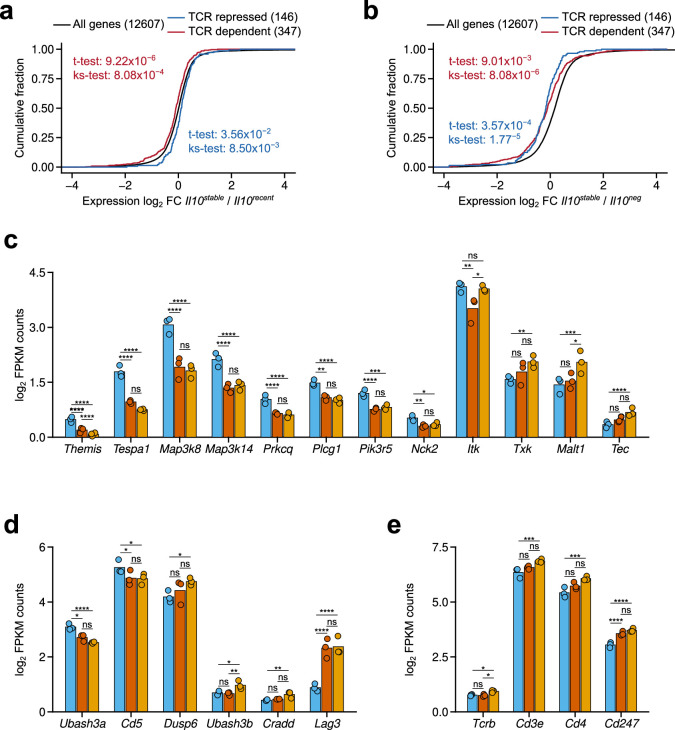
TCR dependent gene expression is attenuated in *Il10*^*stable*^ colonic Treg cells. Data are from bulk RNA-seq analysis presented in Fig. 2. **a**, **b**. Cumulative distribution function plot of mean log_2_ transformed gene expression fold change for *Il10*^*stable*^ versus *Il10*^*recent*^ (**a**) and *Il10*^*stable*^ versus *Il10*^*neg*^ (**b**) samples. All genes (black) and genes with increased (red) or decreased (blue) expression in TCR sufficient versus deficient Treg cells. *P*-values calculated by two-sided *t*-test and two-sided Kolmogorov-Smirnov test for genes with increased (red) or decreased (blue) expression versus all genes are indicated. **c**-**e**. Plots showing log_2_ transformed FPKM counts of indicated genes. Coloring indicates cell population. Negative binomial fitting with two-sided Wald’s significance test and the Benjamini & Hochberg correction for multiple comparisons. Significance testing and correction were performed on all genes. *P*-value > 0.05 ns, < 0.05 *, < 0.01 **, <0.001 ***, < 0.0001 ****. Source data

The data thus far suggested that effects downstream of TCR signaling were distinct among different colonic T_reg_ cell subsets and that IL-10^+^ colonic T_reg_ cells undergoing terminal differentiation had progressively diminished TCR signaling. Supporting this notion, expression of TCR-induced genes was overall decreased, whereas expression of a TCR-repressed geneset was overall increased in *Il10*^stable^ versus *Il10*^recent^ cells^52^ (Extended Data Fig. 4a). Although the TCR-activated geneset also had decreased expression in *Il10*^stable^ versus *Il10*^neg^ cells, the TCR-repressed genes did as well, suggesting that TCR signaling is somewhat attenuated in both *Il10*^stable^ and *Il10*^neg^ T_reg_ cells, albeit in qualitatively distinct ways (Extended Data Fig. 4b). This latter possibility was consistent with the association of Nfatc1 motifs, contrary to other TCR signaling-dependent motifs, with increased accessibility of peaks at cluster bIV genes in *Il10*^stable^ versus *Il10*^neg^ cells. Genes encoding proteins that promote TCR signaling were differentially expressed among *Il10*^neg^, *Il10*^recent^ and *Il10*^stable^ cells, with most having decreased expression in the IL-10^+^ populations (Extended Data Fig. 4c). Conversely, negative regulators of TCR signaling were highly expressed in *Il10*^stable^ cells, including significantly higher expression of *Ubash3b* in *Il10*^stable^ versus *Il10*^recent^ cells (Extended Data Fig. 4d). Finally, TCR component trancripts such as Cd247, Cd3e, and Tcrb, alongside Cd4 were more highly expressed in *Il10*^stable^ versus *Il10*^neg^ T_reg_ cells, with *Tcrb* also being significantly increased in *Il10*^stable^ versus *Il10*^recent^ cells (Extended Data Fig. 4e). Given that these genes are transcriptionally repressed with TCR stimulation, this observation was consistent with attenuated TCR signaling in *Il10*^stable^ cells^53^.

**Fig. 6:**
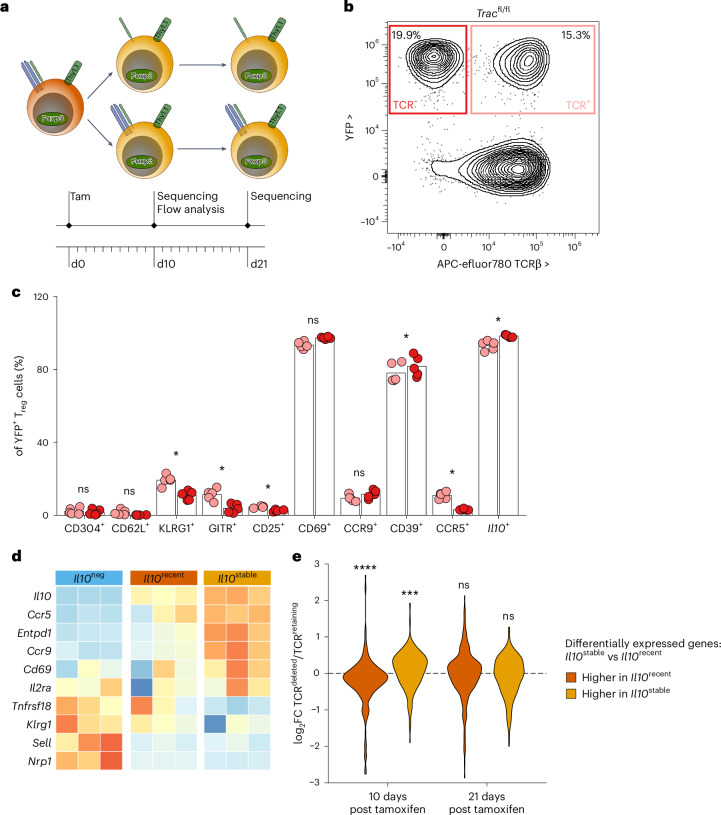
Characterization of *Il10*^stable^ cells maintained in a TCR-independent manner. **a**, Experimental schematic. 10-week-old male and female *Il10*^iΔTCR^ mice were treated with tamoxifen on day 0 and cells were isolated from the LILP on day 10 for flow cytometric analysis (**c**) or TCR-sufficient and TCR-deficient T_reg_ cells were sorted on days 10 and 21 after tamoxifen administration and subjected to RNA-seq analysis (**e**). **b**, Representative two-dimensional flow cytometry plots pre-gated on T_reg_ cells (Thy1.1^+^CD4^+^CD90^+^CD5^+^) from the LILP of *Il10*^*iΔTCR*^ mice. Plots show YFP expression (*y* axis) and TCRβ cell surface expression (*x* axis). **c**, Frequencies of cells positive for the indicated molecules among TCR-sufficient (pink) or TCR-deficient (red) YFP^+^ T_reg_ cells isolated from the LILP of *Il10*^iΔTCR^ mice. Paired two-sided *t*-tests corrected for multiple comparisons using the Benjamini–Hochberg FDR method. Each point represents an individual mouse (*n* = 5, with TCR-sufficient and TCR-deficient cells in each mouse) and represents data pooled from two independent experiments. **d**, Row *Z*-score-normalized log_2_ expression across *Il10*^neg^, *Il10*^recent^ and *Il10*^stable^ T_reg_ cell samples for genes encoding the molecules assessed in **c**. Data are from RNA-seq analysis presented in Fig. 2. **e**, Violin plots of log_2_-transformed gene expression fold change for TCR-sufficient versus TCR-deficient cells at 10 or 21 days post tamoxifen treatment. Genes differentially expressed between *Il10*^stable^ and *Il10*^recent^ T_reg_ cell populations are divided into those with significantly increased expression in *Il10*^stable^ T_reg_ cells (yellow) or *Il10*^recent^ cells (orange). *P* values calculated by two-sided Kolmogorov–Smirnov test for log_2_FC of genes with significantly increased expression in *Il10*^stable^ T_reg_ cells (yellow) or *Il10*^recent^ cells (orange) versus all genes are indicated. ns, *P* > 0.05; *P < 0.05; ***P < 0.001; ****P < 0.0001. Source data

These observations raised the possibility that the functions of *Il10*^stable^ T_reg_ cells might be TCR-independent and that loss of the TCR might even increase the proportion of effector IL-10^+^ T_reg_ cells undergoing terminal differentiation. We directly tested these possibilities through inducible ablation of the TCR in IL-10^+^ cells in *Il10*^tdTomato-CreER^*Trac*^fl/fl^ (*Il10*^iΔTCR^) mice. Tamoxifen-induced deletion of the conditional *Trac* allele leads to loss of the TCRα chain, and thus of the entire TCR signaling complex from the cell surface, enabling identification of TCR-deleted cells by TCRβ cell surface expression^54,55^ (Fig. 6b). These mice also harbored the *Gt(ROSA)26Sor*^*LSL-YFP*^ recombination reporter allele, allowing us to track tagged cells that lost or retained the TCR over time. We treated *Il10*^iΔTCR^ mice with tamoxifen and analyzed YFP^+^ colonic T_reg_ cells 10 days later (Fig. 6a). For various molecules whose expression was enriched in *Il10*^stable^ cells, we saw unchanged or even increased proportions of cells expressing these markers among TCR-negative cells (Fig. 6c-d). This included IL-10 itself as well as CD39 (*Entpd1*), CD69 and CCR9. However, this was not universal, as the expression of several proteins encoded by transcripts highly expressed in *Il10*^stable^ cells was TCR-dependent, such as CD25 (*Il2ra*) and CCR5 (Fig. 6c,d). Finally, the expression of proteins encoded by genes associated with the *Il10*^neg^ population, such as GITR (*Tnfrsf18*) and KLRG1, was further diminished in IL-10^+^ cells after TCR deletion (Fig. 6c,d). Overall, these data support the notion that the diminution of TCR signaling supports the terminal differentiation of *Il10*^stable^ T_reg_ cells and that, conversely, ongoing TCR stimulation maintains some phenotypic ‘plasticity’ of T_reg_ cells.

To broadly characterize the changes caused by TCR ablation in *Il10*^stable^ colonic T_reg_ cells, we performed RNA-seq analysis of TCR-sufficient and TCR-deficient YFP^+^ T_reg_ cells isolated from the colons of *Il10*^iΔTCR^ mice 10 days and 21 days after tamoxifen treatment. We assessed the expression of genes differentially expressed in *Il10*^stable^ versus *Il10*^recent^ T_reg_ cells; that is, genes with increasing or decreasing expression during the differentiation of *Il10*^stable^ cells. Overall, the genes more highly expressed in *Il10*^recent^ T_reg_ cells showed lower expression in TCR-deficient versus TCR-sufficient T_reg_ cells, whereas those that were increased in expression in *Il10*^stable^ cells had overall higher expression in TCR-deficient cells (Fig. 6e). Of note, this effect was more pronounced at day 10 versus day 21, suggesting that at the latter timepoint, even the TCR-sufficient YFP^+^ cells had largely completed their terminal differentiation (Fig. 6e). Overall, this was consistent with the notion that terminal differentiation of IL-10^+^ effector T_reg_ cells was constrained by TCR signaling and that its diminution facilitated this process.

### Colonic *Il10*^stable^ T_reg_ cells promote tissue health

Terminally differentiated effector T cells are often characterized as exhausted or dysfunctional relative to their source (‘stem-like’) effector T cells: contributing minimally to clearing infectious agents, controlling tumor progression or causing autoimmune disease^56–59^. However, this notion largely comes from studies of CD8^+^ T cells, and it remains unclear whether this extends to T_reg_ cells. Given the apparent terminal differentiation of colonic *Il10*^stable^ T_reg_ cells, we wondered to what extent these cells remain functional and contribute to immune regulation. Considering the high expression of IL-10 by this population and the known role of T_reg_ cell-derived IL-10 in preventing spontaneous colitis, one could assume that *Il10*^stable^ T_reg_ cells might be an important source of this cytokine^32,60^. However, previous studies of the role of T_reg_ cell-derived IL-10 using *Foxp3*^Cre^*Il10*^fl/fl^ mice relied on constitutive ablation of IL-10 expression from T_reg_ cells throughout the lifespan of the mice, including the critical early life period of microbial colonization. Therefore, colitis reported in these animals might be caused by the loss of T_reg_ cell-derived IL-10 during a critical developmental window rather than its continuous production by a specialized colonic population. Indeed, studies of T_reg_ cells during the time of weaning and microbial community assembly support this notion^61,62^.

**Extended Data Fig. 5:**
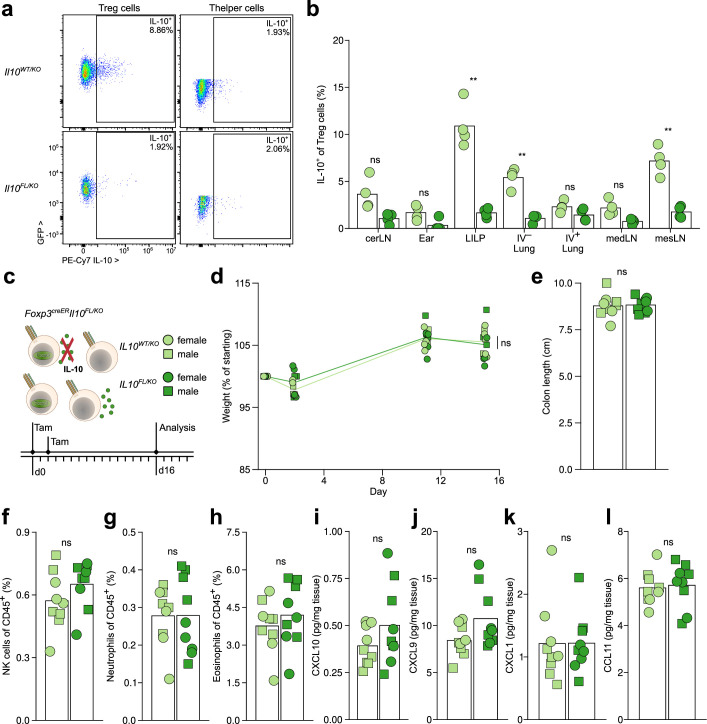
Loss of IL-10 expression by Treg cells in adult mice does not result in colonic inflammation. **a**-**l**. 8- to 10-week-old *Foxp3*^*CreER*^*Il10*^*FL/KO*^ and littermate control *Foxp3*^*CreER*^*Il10*^*WT/KO*^ mice were treated with tamoxifen on days 0 and 2 and analyzed on day 16. **a**, **b**. Representative 2D flow plots showing IL-10 production by Treg cells (GFP^+^CD4^+^TCRβ^+^) and conventional activated CD4^+^ T cells (Tconv) (GFP^–^CD44^+^CD4^+^TCRβ^+^) in the colon (**a**) and plot showing frequencies of IL-10^+^ Treg cells in various tissues (**b**). Each point represents an individual mouse (n = 4 per genotype), and data are representative of three independent experiments (**b**). **c**. Diagram depicting strategy for ablation of IL-10 production by Treg cells. Legend for all plots: *Foxp3*^*CreER*^*Il10*^*WT/KO*^ (light green) and *Foxp3*^*CreER*^*Il10*^*FL/KO*^ (dark green), and female (circles) or male (squares) mice. Experimental treatment regimen timeline. **d**. Plot showing weights over time, normalized to starting body weight for each mouse. ANOVA for weight change as a function of genotype, sex, replicate, and time with *p*-value for the effect of treatment determined by Tukey’s ‘Honest Significant Difference’ method. **e**. Plot showing colon lengths on day 16. ANOVA for colon length as a function of treatment, sex, and replicate with *p*-value for the effect of treatment determined by Tukey’s ‘Honest Significant Difference’ method. **f**-**h**. Flow cytometry of LILP from DT and bDT treated mice. Plots depict frequencies of NK cells (**f**), neutrophils (**g**), and eosinophils (**h**) among all live CD45^+^ cells. See Methods for cell gating strategies. **i**-**l**. Protein was extracted from colonic tissue and chemokines quantified using a multiplexed bead assay. Plots depict abundance (normalized to tissue weight) of CXCL10 (**i**), CXCL9 (**j**), CXCL1 (**k**), and CCL11 (**l**). (**d**-**l**) Each point represents an individual mouse. Data shown are pooled from three independent experiments (n = 9 per group). (**f**-**l**) ANOVA for variable as a function of treatment, sex, and experimental replicate with *p*-value for the effect of treatment determined by Tukey’s ‘Honest Significant Difference’ method and then corrected for multiple comparisons (all measured parameters) using the FDR method of Benjamini and Hochberg. *P*-value > 0.05 ns. Source data

**Extended Data Fig. 6:**
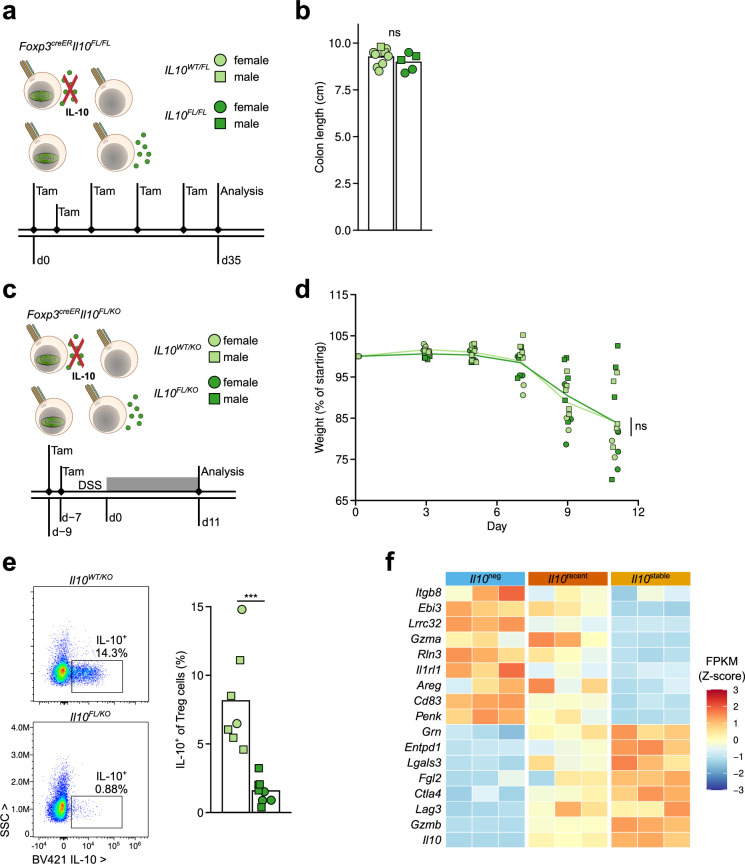
Loss of IL-10 expression by Treg cells does not exacerbate chemically induced colitis. **a**-**b**. 8- to 10-week-old *Foxp3*^*CreER*^*Il10*^*FL/FL*^ and littermate control *Foxp3*^*CreER*^*Il10*^*WT/FL*^ mice were treated with tamoxifen on days 0, 4, 11, 18, 25, and 32 before analysis on day 35. **a**. Diagram depicting strategy for ablation of IL-10 production by Treg cells. Legend for all plots: *Foxp3*^*CreER*^*Il10*^*WT/KO*^ (light green) and *Foxp3*^*CreER*^*Il10*^*FL/KO*^ (dark green), and female (circles) or male (squares) mice. Experimental treatment regimen timeline. **b**. Plot showing colon lengths on day 3. Each point represents an individual mouse (n = 5-9) and data are pooled from two independent experiments. ANOVA for colon length as a function of genotype, sex and replicate, with *p*-value for the effect of genotype determined by Tukey’s ‘Honest Significant Difference’ method. **c**-**e**. 11- to 14-week-old *Foxp3*^*CreER*^*Il10*^*FL/KO*^ and *Foxp3*^*CreER*^*Il10*^*WT/KO*^ male and female mice were treated with tamoxifen on days −9 and −7 and cells and were given DSS in drinking water from day 0. **c**. Diagram depicting experimental strategy. Legend for all plots: *Foxp3*^*CreER*^*Il10*^*WT/KO*^ (light green) and *Foxp3*^*CreER*^*Il10*^*FL/KO*^ (dark green), and female (circles) or male (squares) mice. Experimental treatment regimen timeline. **d**. Plot showing weights over time, normalized to starting (day 0) body weight for each mouse. ANOVA for weight change as a function of genotype and time with *p*-value for the effect of genotype determined by Tukey’s ‘Honest Significant Difference’ method. **e**. Representative 2D flow plots (left) and plot showing frequencies (right) of IL-10 production by Treg cells (GFP^+^CD4^+^TCRβ^+^) in the mesenteric lymph. (left) A single mouse, representative of 7 mice of each genotype from two experiments. (right) Each point represents an individual mouse (n = 7 per genotype), and data are representative of two independent experiments. One-way ANOVA for frequency as a function of genotype. **f**. Row Z-score normalized log_2_ expression (FPKM) across *Il10*^*neg*^, *Il10*^*recent*^, and *Il10*^*stable*^ Treg cell samples for genes encoding select known effectors of Treg cell function. Data are from RNA-seq analysis presented in Fig. 2. *P*-value > 0.05 ns, < 0.01 **, <0.001 ***. Source data

Therefore, we sought to assess the need for ongoing IL-10 production by T_reg_ cells using tamoxifen-inducible ablation in healthy adult *Foxp3*^CreER^*Il10*^fl^ mice. *Foxp3*^CreER^*Il10*^fl/KO^ and littermate control *Foxp3*^CreER^*Il10*^WT/KO^ mice were treated with two doses of tamoxifen to achieve loss of IL-10 secretion by T_reg_ cells and then analyzed 16 days later (Extended Data Fig. 5a–c). Unexpectedly, these mice showed no signs of inflammatory disease as assessed by weight loss or colon shortening (Extended Data Fig. 5d,e). This observation suggested that continuous production of IL-10 by T_reg_ cells was dispensable for preventing colonic inflammation in adult mice and appeared to support the notion that terminally differentiated colonic *Il10*^stable^ T_reg_ cells were non-functional, or at least redundant in local immune regulation. We confirmed this finding in a longer-duration experiment, with no changes in colon length observed after 5 weeks of IL-10 ablation in T_reg_ cells (Extended Data Fig. 6a,b). Furthermore, induced IL-10 deficiency in T_reg_ cells did not further exacerbate weight loss in the DSS-induced colitis model compared to identically treated littermate controls (Extended Data Fig. 6c–e).

The above reasoning relies on the assumption that IL-10 production was the dominant effector modality of colonic *Il10*^stable^ T_reg_ cells. However, based on their gene expression program, IL-10 could constitute just one of multiple, potentially redundant immune regulatory mechanisms deployed by these cells. Indeed, colonic IL-10^+^ T_reg_ cells, in addition to IL-10, were enriched for expression of numerous effector molecules, including CD39, granzyme B, galectin-3, fibroleukin (*Fgl2*) and CTLA-4, whereas their IL-10 non-expressing counterparts expressed a distinct set of effector molecules (Extended Data Fig. 6f and Fig. 2a).

**Fig. 7:**
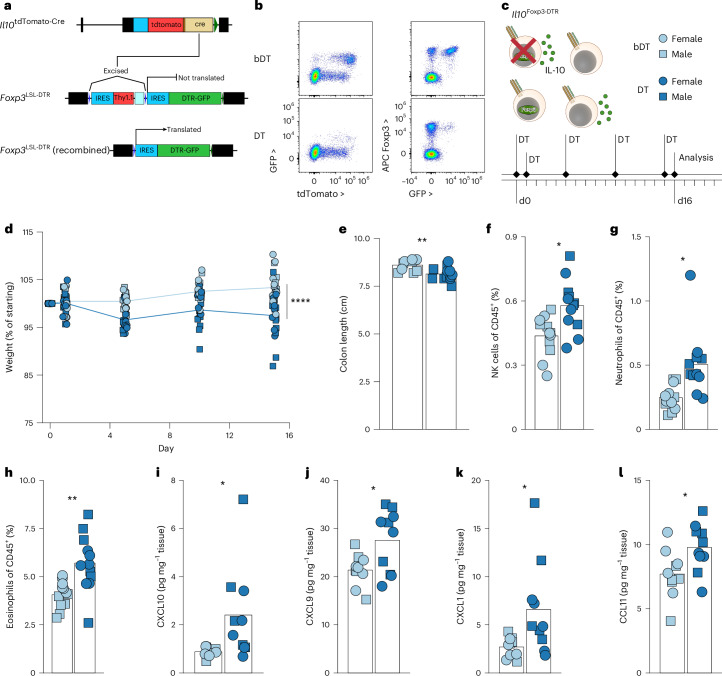
Acute ablation of IL-10^+^ T_reg_ cells results in colonic inflammation. **a**, Schematic of targeted *Il10* and *Foxp3* loci in *Il10*^tdTomato-Cre^ and *Foxp3*^LSL-DTR^ mice. Bottom panel shows recombined (LSL cassette deleted by Cre recombinase) allele permitting DTR expression. See Methods for details. **b**–**l**, *Il10*^tdTomato-Cre^*Foxp3*^LSL-DTR^ (*Il10*^Foxp3-DTR^) mice (8–11 weeks old) were treated with active (DT) or heat-inactivated (bDT) diphtheria toxin over 16 days. **b,** Representative two-dimensional flow plots showing IL-10 (tdTomato) by GFP (left) and GFP versus Foxp3 protein (right) expression in CD4^+^ T cells. **c,** Diagram depicting strategy for ablation of IL-10^+^ T_reg_ cells in *Il10*^Foxp3-DTR^ mice. For all plots, DT, light blue; bDT, dark blue; females, circles; males, squares. Experimental treatment regimen timeline. **d**, Plot showing weights over time, normalized to starting body weight for each mouse. Data are pooled from five independent experiments (*n* = 19 for each group overall). ANOVA for weight change as a function of treatment, sex, replicate and time, with *P* value for the effect of treatment determined by Tukey’s honest significant difference method. **e,** Plot showing colon lengths on day 16 of DT-treated and bDT-treated mice. Data are pooled from five independent experiments (*n* = 19 for each group overall). **f**–**h**, Flow cytometry of LILP from DT-treated and bDT-treated mice. Plots depict frequencies of natural killer (NK) cells (**f**), neutrophils (**g**) and eosinophils (**h**) among all live CD45^+^ cells. Data shown are pooled from three independent experiments (*n* = 12 per group). **i**–**l**, Protein was extracted from colonic tissue and chemokines were quantified. Plots depict the abundance (normalized to weight of tissue) of CXCL10 (**i**), CXCL9 (**j**), CXCL1 (**k**) and CCL11 (**l**). Data shown are from tissues from three independent experiments (*n* = 10 per group). In **d**–**l**, each point represents an individual mouse; in **e**–**l,** ANOVA for variable as a function of treatment, sex and experimental replicate, with *P* value for the effect of treatment determined by Tukey’s honest significant difference method; in **f**–**l**, corrections for multiple comparisons (all measured parameters) made using the Benjamini–Hochberg FDR method. ns, *P* > 0.05; *P < 0.05; **P < 0.01; ****P < 0.0001. Source data

**Extended Data Fig. 7:**
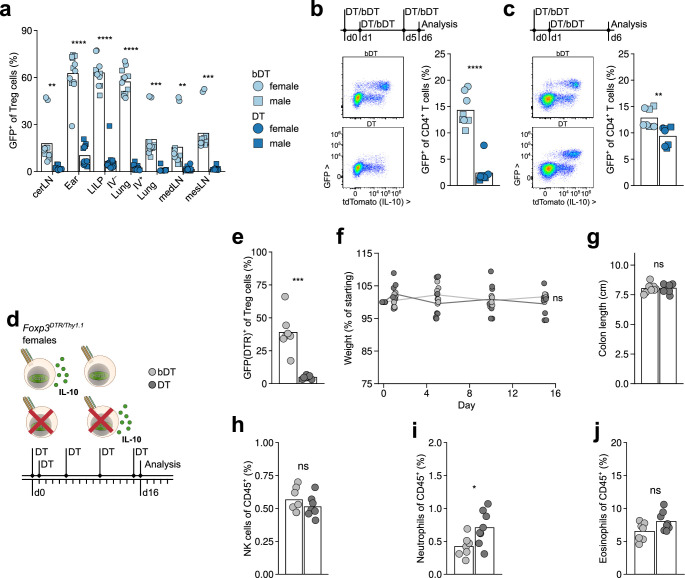
Analysis of IL-10 expressing Treg cell depletion. **a**-**c**. 8- to 11-week-old *Il10*^*tdTomato-Cre*^*Foxp3*^*LSL-DTR*^ (*Il10*^*Foxp3-DTR*^) mice were treated with active (DT) or heat-inactivated (boiled, bDT) diphtheria toxin on days 0, 1, 5, 10, 15 (**a**); days 0, 1, 5 (**b**); or days 0, 1 (**c**) before analysis on day 16 (**a**) or day 6 (**b**, **c**). **a**. Plots showing frequency of GFP^+^ cells among Treg cells in indicated tissues. Each point represents an individual mouse (n = 12 per group) and data shown are pooled from three independent experiments. **b**, **c**. Frequencies (right) of GFP^+^ cells among CD4^+^ T cells from the colonic lamina propria. Each point represents an individual mouse (n = 8 per group) and data shown are pooled from two independent experiments for each regimen. Two-sided unpaired Student’s t-tests. **d**-**j**. 8- to 11-week-old *Il10*^*tdTomato-Cre*^*Foxp3*^*GFP-DTR/Thy1.1*^ (*Foxp3*^*DTR-het*^) mice were treated with active (DT) or heat-inactivated (boiled, bDT) diphtheria toxin over 16 days. **d**. Diagram depicting experimental strategy. Legend for all plots: DT (light grey) and bDT (dark grey). Experimental treatment regimen timeline (bottom). **e**. Plot showing frequency of GFP-DTR^+^ Treg cells among all Treg cells in the LILP. **f**. Plot showing weights over time, normalized to starting body weight for each mouse. ANOVA for weight change as a function of treatment, replicate, and time with *p*-value for the effect of treatment determined by Tukey’s ‘Honest Significant Difference’ method. **g**. Plot showing colon lengths on day 16. **h**-**j**. Plots depict frequencies of NK cells (**j**), neutrophils (k), and eosinophils (l) among all live CD45^+^ cells. **a**, **e**, **g**, **h**-**j**. ANOVA for variable as a function of treatment and replicate with *p*-value for the effect of treatment determined by Tukey’s ‘Honest Significant Difference’ method. Corrected for multiple comparisons using the FDR method of Benjamini and Hochberg (**a**, **e**, **h**-**j**). **e**-**j**. Each point represents an individual mouse (n = 7 mice in bDT, 8 mice in DT groups) and data shown are pooled from two independent experiments. *P*-value < 0.01 **, <0.001 ***, <0.0001 ****. Source data

Therefore, ablating IL-10^+^ T_reg_ cells rather than their IL-10-producing capacity was required for rigorous testing of their function. We therefore developed a novel model for selective ablation of IL-10^+^ T_reg_ cells by engineering a *Foxp3* knock-in allele harboring a loxP–STOP–loxP (LSL) cassette upstream of the coding sequence for the simian diphtheria toxin receptor (DTR) in the 3′ UTR (Fig. 7a). In these *Foxp3*^LSL-DTR^ mice, DTR expression by Foxp3^+^ T_reg_ cells requires a Cre recombinase from a locus of interest. We therefore generated *Il10*^tdTomato-Cre^*Foxp3*^LSL-DTR^ (*Il10*^Foxp3-DTR^) mice to allow for the specific depletion of IL-10 expressing T_reg_ cells by diphtheria toxin (DT) administration. We confirmed that treating mice with DT, but not heat-inactivated DT (boiled, bDT) resulted in the loss of IL-10^+^ T_reg_ cells while sparing other IL-10^+^ populations and other Foxp3^+^ cells (Fig. 7b and Extended Data Fig. 7a). Transient depletion of IL-10^+^ T_reg_ cells in *Il10*^Foxp3-DTR^ mice upon administration of DT followed by a brief recovery revealed that these cells quickly repopulated the colon, suggesting that any progenitor population was spared by our depletion strategy and that it enabled probing the function of terminally differentiated cells (Extended Data Fig. 7b,c).

Depletion of IL-10^+^ T_reg_ cells by DT treatment of *Il10*^Foxp3-DTR^ mice resulted in notable colon shortening and weight loss by day 16 in comparison to bDT-treated controls (Fig. 7c–e). This was accompanied by increased frequencies of natural killer cells, neutrophils and eosinophils in the colonic lamina propria (Fig. 7f–h). Increased abundance of the inflammatory chemokines responsible for recruiting these populations, CXCL9/10, CXCL1 and CCL11, suggested that loss of IL-10^+^ T_reg_ cells resulted in a broadly heightened inflammatory state of the colon (Fig. 7i–l). Importantly, neither the inflammatory cell populations nor the chemo-attractants were increased after the loss of T_reg_ cell-derived IL-10, consistent with the notion that the function of *Il10*^stable^ T_reg_ cells was not limited to IL-10 production (Extended Data Fig. 5f–l). Collectively, these results suggested that rather than being dysfunctional, IL-10^+^ T_reg_ cells represented a specialized population of effectors supporting colonic health.

**Fig. 8:**
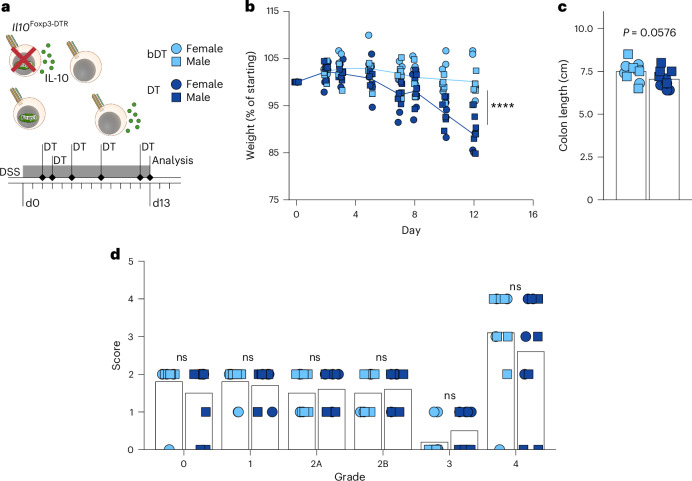
Acute ablation of IL-10^+^ T_reg_ cells increases colitis pathology. *Il10*^Foxp3-DTR^ mice (8–10 weeks old) were given DSS (1.5% w/v) in drinking water and then treated with active (DT) or heat-inactivated (bDT) diphtheria toxin over 11 days before analysis by flow cytometry on day 13 after DSS initiation. For all plots, DT, light blue; bDT, dark blue; females, circles; males, squares. **a**, Diagram depicting DT-induced ablation of IL-10^+^ T_reg_ cells in *Il10*^Foxp3-DTR^ mice. Experimental treatment regimen timeline. **b**, Plot showing weights over time, normalized to starting body weight for each mouse. ANOVA for weight change as a function of treatment, sex, replicate and time, with *P* value for the effect of treatment determined by Tukey’s honest significant difference method. **c,** Plot showing colon lengths on day 13 of DT-treated and bDT-treated mice. ANOVA for colon length as a function of treatment, sex and replicate, with *P* value for the effect of treatment determined by Tukey’s honest significant difference method. **d**, Histopathological scoring (Simplified Geboes Score rubric) of colon sections collected on day 13 from DT-treated and bDT-treated mice. See Methods for details. ANOVA for score as a function of treatment, sex, grade and experimental replicate, with *P* value for the effect of treatment determined by Tukey’s honest significant difference method. In **b**–**d**, each point represents an individual mouse (*n* = 10 for each group) and data shown are pooled from four independent experiments. ns, *P* > 0.05; ****P < 0.0001. Source data

One caveat to this conclusion is the possibility that the observed inflammation was caused by a non-specific effect of depleting approximately half of colonic T_reg_ population represented by IL-10^+^ T_reg_ cells (Extended Data Fig. 7a). To test this idea, we generated *Il10*^tdTomato-Cre^*Foxp3*^Thy1.1/GFP-DTR^ female mice (hereafter referred to as *Foxp3*^DTR-het^) harboring equal numbers of DT-sensitive GFP-DTR-expressing or DT-resistant Thy1.1-expressing T_reg_ cells owing to random X-inactivation. These mice allowed for the depletion of approximately half of T_reg_ cells regardless of their localization or IL-10 expression (Extended Data Fig. 7d,e). Contrary to depleting IL-10^+^ T_reg_ cells, such ‘subset-unaware’ halving of colonic T_reg_ cells did not result in detectable weight loss or colon shortening, even though a mild increase in innate immune cell infiltration of the colon was observed (Extended Data Fig. 7f–j). This suggested that terminally differentiated IL-10^+^ T_reg_ cells have a non-redundant function in supporting colonic and overall organismal health. To assess a role for these cells in settings of induced colonic injury and inflammation, mice were administered with a low dose of DSS in drinking water for 2 days before and then throughout the ablation of IL-10^+^ T_reg_ cells (Fig. 8a). This treatment resulted in increased weight loss compared to control non-depleted but DSS-treated mice, demonstrating a role for these cells in inflammatory settings (Fig. 8b,c). Evaluation of the colonic tissue did not reveal any obvious worsening of histological signs of pathology following IL-10^+^ T_reg_ cell ablation on the simplified Geboes rubric^63^ (Fig. 8d). This lack of a histopathological difference may be attributed to the severity of tissue damage in this model at the late timepoint analyzed. Together, these studies suggest that IL-10^+^ T_reg_ cells deploy a multitude of redundant mechanisms to maintain colonic health.

### Heterogeneity of intestinal IL-10-expressing T_reg_ cells

**Extended Data Fig. 8:**
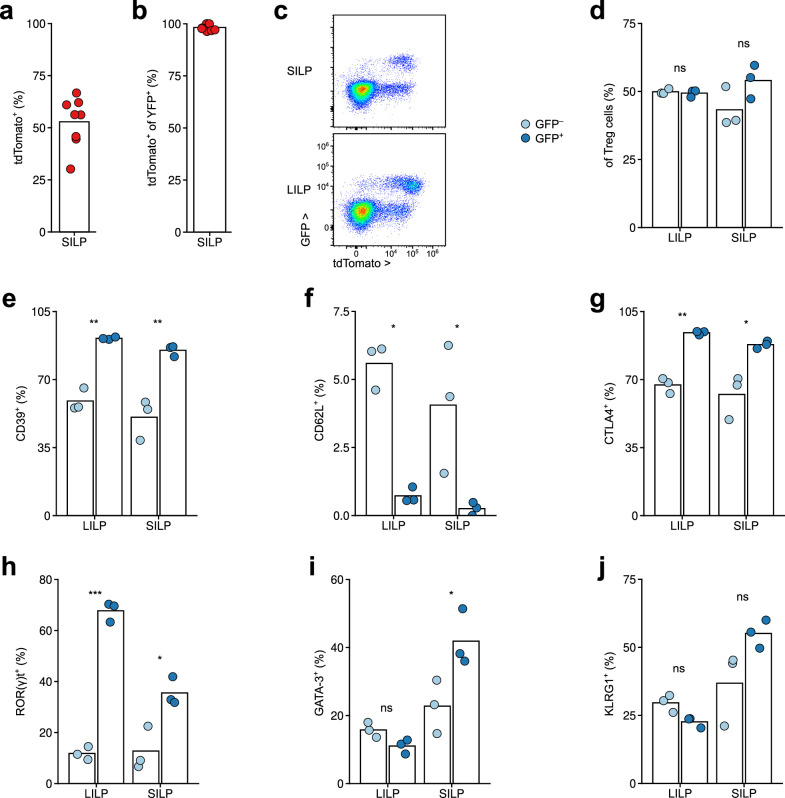
Characterization of small intestinal IL-10 expressing Treg cells. **a**-**b**. *Il10*^*FM*^ mice were treated at 8 weeks of age with tamoxifen and analyzed 21 days later. Frequencies of tdTomato^+^ cells among Treg cells (**a**) and among YFP^+^ Treg cells (**b**) isolated from SILP. Each point represents an individual mouse and data are pooled from two independent experiments (n = 4 mice per replicate). **c**-**j**. Cells were isolated from the SILP and LILP of *Il10*^*Foxp3-DTR*^ mice and analyzed by flow cytometry. Representative 2D flow cytometry plot showing GFP and tdTomato expression among CD4^+^ T cells from the SILP (top) and LILP (bottom) (**c**). Plots showing frequencies of GFP^–^ (light blue) and GFP^+^ (dark blue) Treg cells from the LILP and SILP (**d**). Plots showing frequencies of CD39 (**e**), CD62L (f), CTLA4 (**g**), RORγt (**h**), Gata3 (**i**), and KLRG1 (**j**) expression among GFP^–^ (light blue) and GFP^+^ (dark blue) Treg cells from the LILP and SILP of *Il10*^*Foxp3-DTR*^ mice. (**d**-**j**) Each point represents an individual mouse (n = 3), with paired GFP^–^ and GFP^+^ cells from each mouse. Data are from a single experiment. ANOVA for frequencies as a function of tissue and GFP status with *p*-value for the effect of GFP determined by Tukey’s ‘Honest Significant Difference’ method and then corrected for multiple comparisons using the FDR method of Benjamini and Hochberg. *P*-value > 0.05 ns, < 0.05 *, < 0.01 **, <0.001 ***. Source data

The stability of IL-10 expression among terminally differentiated T_reg_ cells in the colon was consistent with the abundance of IL-10^+^ T_reg_ cells in that tissue and raised the question of whether the colon is unique in enabling terminal differentiation of IL-10^+^ T_reg_ cells^32,47,48,64^. Given the known phenotypic overlap between colon and small intestine (SI) T_reg_ cells and the integrated functions and lymphatic drainage of these tissues, we asked whether this feature was also shared across these tissues. We assessed the stability of IL-10 expression by T_reg_ cells in the SI by analyzing the cells 21 days after tamoxifen treatment of *Il10*^FM^ mice. As in the colon, IL-10^+^ T_reg_ cells in the SI were also *Il10*^stable^ (Extended Data Fig. 8a,b). Further characterization of these *Il10*^stable^ SI T_reg_ cells in *Il10*^Foxp3-DTR^ mice revealed substantial commonality between SI and colonic *Il10*^stable^ T_reg_ cells, including heightened expression of effector molecules CD39 and CTLA-4 and the loss of CD62L expression (Extended Data Fig. 8e–g). At the same time, IL-10^+^ T_reg_ cells in the SI expressed either RORγt or Gata3, in contrast to their colon counterparts, which were overwhelmingly RORγt^+^ (Extended Data Fig. 8h,i). SI IL-10^+^ T_reg_ cells were also distinguished by their expression of the marker KLRG1 (Extended Data Fig. 8j).

**Extended Data Fig. 9:**
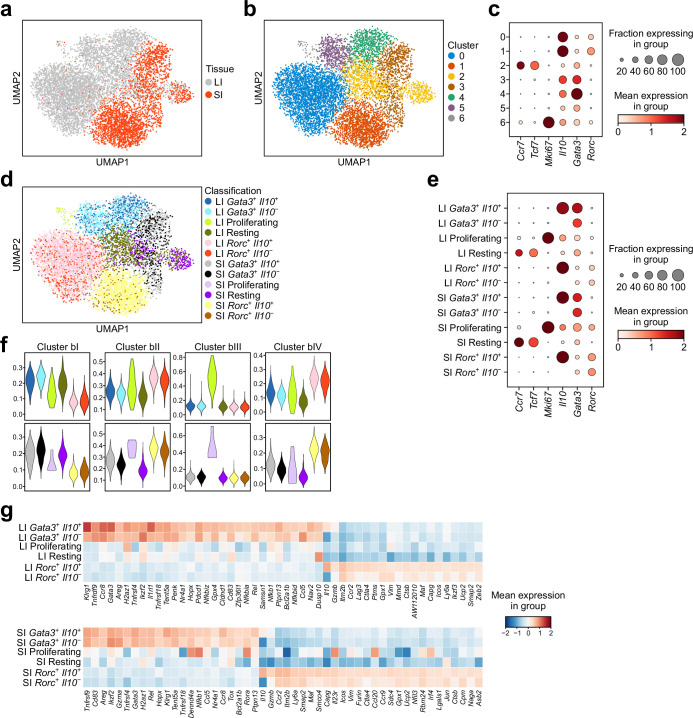
Transcriptional features of SI and colon IL-10^+^ Treg cells. Treg cells (Thy1.1^+^CD4^+^TCRβ^+^) were separately sorted from the Large Intestine (LILP) and Small Intestine Lamina Propria (SILP) processed for 10X paired scRNA-seq and VDJ-seq analysis. See Methods for details. **a**-**b**. Union Manifold Approximation and Projection (UMAP) plot for LILP and SILP Treg cells colored based on tissue of origin (**a**) or clustering based on nearest neighbor graph (**b**). **c**. Expression of indicated genes in each cluster. Size of the dot in the dot plot indicates proportion of cells expressing the gene in each cluster and color indicates mean expression value of the gene in a cluster. **d**-**e**. UMAP plot (**d**) or dot plot showing expression of indicated genes (**e**) for annotated LILP and SILP Treg cell clusters. **f**. The annotated LILP or SILP Treg cell clusters were assessed for expression of bulk RNA-seq gene clusters (bI-IV); violin plots depict gene cluster score by each annotated scRNA-seq cell cluster. **g**. Top differentially expressed genes in *Gata3*^+^*Il10*^+^ Treg cells and *Rorc*^*+*^*Il10*^*+*^ Treg cells from LILP (top) and SILP (bottom) (adjusted *p*-value < 0.05 & log_10_ fold change > 0.5). Color indicates mean Z-score value. Source data

**Extended Data Fig. 10:**
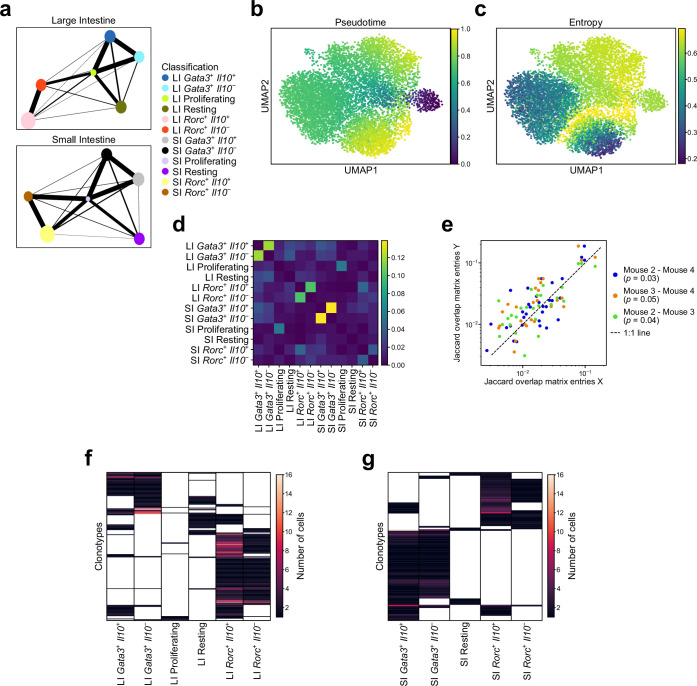
Relatedness of IL-10^+^ and IL-10^−^ Treg cells from the SI and colon. Analysis of scRNA-seq data presented in Extended Data Fig. 9. **a**. Partition-based graph abstraction plot for LILP and SILP Treg cells. Color of each node indicates cluster annotation as in Extended Data Fig. 9d; thickness of the edge and distance from the other clusters indicate degree of transcriptional connectivity/proximity between each cluster. **b**, **c**. UMAP plot depicting pseudotime (**b**) or entropy values (**c**) of LILP and SILP Treg cells. Shading represents the value of pseudotime (**b**) or entropy (**c**) values. **d**, **e**. Jaccard overlap between annotated Treg cell clusters, for cells pooled across three mice (**d**) and correlation between mouse-level Jaccard overlap matrices (**e**). **f**, **g**. Clonotype sharing across clusters in the LILP (**f**) and SILP (**g**). Source data

The observed heterogeneity of RORγt and Gata3 expression within SI IL-10^+^ T_reg_ cells raised the question of the relatedness between these subsets and their colonic counterparts. We addressed this question by performing paired scRNA-seq and TCR-seq analysis of total colon and SI T_reg_ cells. T_reg_ cells from both tissues comprised clusters distinguished by *Rorc* (clusters 0 and 1) or *Gata3* expression (clusters 3–5) and clusters enriched for genes associated with either quiescence (*Ccr7*, *Tcf7*; cluster 2) or proliferation (*Mki67*; cluster 6) (Extended Data Fig. 9a–c). To directly assess IL-10^+^ and IL-10^−^ T_reg_ subsets expressing either RORγt or Gata3, we annotated each cell based on the tissue of origin (colon or SI), *Rorc* (Rorc^+^) or *Gata3* expression (Gata3^+^), enrichment for quiescence (‘resting’) and cell cycle (‘proliferating’) and *Il10* expression (*Il10*^+^ or *Il10*^−^) (Extended Data Fig. 9d,e). IL-10 was detected in the majority of cells in the *Rorc*^+^ cluster, as well as some in the *Gata3*^+^ cluster (Extended Data Fig. 9c). Projecting expression of the bulk RNA-seq gene clusters bI–bIV (Fig. 2c) to the annotated scRNA-seq T_reg_ cell clusters suggested that *Rorc*^+^ T_reg_ cells in both colon and SI represented *Il10*^stable^ T_reg_ cells, as they were enriched for expression of cluster bII and bIV genes (Extended Data Fig. 9f). Interestingly, both colon and SI *Gata3*^+^ cells were enriched for genes expressed by *Il10*^neg^ T_reg_ cells (cluster bI) (Extended Data Fig. 9f) regardless of their IL-10 expression. Again, ‘proliferating’ cells represented *Il10*^recent^ T_reg_ cells (cluster bIII) (Fig. 4a,b and Extended Data Fig. 9f). *Gata3*^+^*Il10*^+^ T_reg_ cells were transcriptionally distinct from *Rorc*^+^*Il10*^+^ T_reg_ cells despite shared IL-10 expression (Extended Data Fig. 9g). Partition-based graph abstraction (PAGA) analysis of T_reg_ cell subsets further confirmed that *Gata3*^+^*Il10*^+^ and *Rorc*^+^*Il10*^+^ T_reg_ cells were transcriptionally distant and closer to their *Il10*^−^ counterparts (Extended Data Figs. 9d and 10a). Additionally, *Gata3*^+^ and *Rorc*^+^ T_reg_ cells were closer to ‘proliferating’ T_reg_ cells, suggesting that ‘proliferating’ T_reg_ cells may serve as a pool of progenitors for these T_reg_ cell subsets (Extended Data Figs. 9d and 10a). Analysis of differentiation potential using the Palantir algorithm showed that both colonic and SI *Il10*^+^*Rorc*^+^ but not *Il10*^+^*Gata3*^+^ cell clusters exhibited low entropy values consistent with the terminal differentiation state of the former (Extended Data Fig. 10b,c). We also examined TCR usage by the identified SI and colonic T_reg_ cell subsets. The highest degree of clonotype overlap was observed between *Il10*^+^ and *Il10*^−^ counterparts within either *Gata3*^+^ or *Rorc*^+^ populations, suggesting that IL-10^+^ Gata3^+^ and RORγt^+^ T_reg_ cells arise from a largely non-overlapping pool of precursor cells in both tissues (Extended Data Fig. 10d–g).

## Discussion

Using genetic cell tracking and targeting, we demonstrate that stable expression of the immunomodulatory cytokine IL-10 defines terminally differentiated effector T_reg_ cells in the colon with an essential function in colonic health. IL-10^+^ T_reg_ cells in the colon seemed to adopt this fate following proliferative expansion. Although less well characterized, the *Il10*^neg^ T_reg_ cells in the colon appeared to harbor distinct varieties of effector T_reg_ cells. Importantly, our analysis of the consequences of antibiotic-induced depletion of the microbiota or bleomycin-induced lung inflammation suggests that ongoing inflammatory exposure is not necessary to maintain, and not sufficient to induce, stable IL-10 expression by activated T_reg_ cells. The observation that SI alongside colon, but not other tissues, afforded stable IL-10 expression by T_reg_ cells suggested a distinct ability of these tissue environments to support the generation of *Il10*^stable^ T_reg_ cells. Considering that the majority of *Il10*^stable^ T_reg_ cells express RORγt, they probably represent extrathymically generated T_reg_ cells.

Analysis of transcriptomes and epigenomes of *Il10*^neg^, *Il10*^recent^ and *Il10*^stable^ cells suggested that attenuated TCR signaling alongside Maf and RORγt TF activities impart the distinct features of terminally differentiated *Il10*^stable^ T_reg_ cells. The importance of Maf and RORγt for a population of colonic T_reg_ cells has been demonstrated, supporting our results^38,40,47–49^. Although the importance of TCR signaling in T_reg_ cells for the induction of IL-10 is well established, our analysis of T_reg_ cells post-activation suggests that ongoing TCR signaling opposes stable IL-10 expression and, more broadly, terminal differentiation. This hypothesis was supported by the deletion of TCR expression after the acquisition of IL-10 expression. That the identified population of IL-10-expressing T_reg_ cells becomes independent of TCR signaling upon their terminal differentiation suggests the acquisition of an ‘innate-like’ function in these cells. This ‘innate-like’ functional state is reminiscent of other T cell populations, which have been shown to acquire TCR-independent functionality when highly differentiated^55,65–69^. This finding suggests that loss of TCR dependence for function represents a common if not universal feature among terminally differentiated T cell populations. Furthermore, in contrast to studies of CD8^+^ T cells, which suggest that immune responses are reliant on ‘stem-like’ populations rather than their dysfunctional terminally differentiated progeny, our experiments suggest that terminally differentiated T_reg_ cells have an essential role in controlling inflammatory processes and maintaining colonic health^56–59^.

Although our experiments do not indicate whether ongoing TCR stimulation is required to specify the *Il10*^neg^ state or that attenuation of TCR signaling is required to calcify the *Il10*^stable^ state, the analysis of cells from *Il10*^iΔTCR^ mice favors the latter possibility. It is possible that TCR signaling persists in both populations but is qualitatively distinct, with diminished output from specific signaling cascades in *Il10*^stable^ cells. In this regard, Nr4a2 is a candidate TF favoring the *Il10*^neg^ over the *Il10*^stable^ T_reg_ cell program, given that *Nr4a2* expression and apparent TF activity is specifically diminished in *Il10*^stable^ T_reg_ cells.

Previous investigation of TFs involved in the specification of tissue T_reg_ cells identified Batf/BATF as important in facilitating their tissue-supportive functions in both mouse and human^25,26^. Interestingly, the Batf motif appears in our analysis as associated with increased accessibility of gene loci with increased expression in *Il10*^neg^ cells. This observation suggests that *Il10*^neg^ cells in the colon share features with T_reg_ cells performing tissue-supportive functions in other organs. However, the *Il10*^stable^ T_reg_ cells also have high expression of specific genes encoding molecules known to support tissue function. Although we did not exclude a role for Batf in *Il10*^stable^ T_reg_ cell function, our results suggest that tissue-supportive functions are not restricted to a specific population of T_reg_ cells and are not exclusively controlled by Batf.

Our study identifies transcriptional programs and highlights signaling pathways supporting the specification of distinct populations of colonic T_reg_ cells. In addition, we demonstrate that a subset of colonic T_reg_ cells undergo functional specialization, assuming a stable terminally differentiated state that is robust to environmental perturbations and not reliant on ongoing conditioning for its maintenance. The importance of these terminally differentiated T_reg_ cells in preventing local inflammation contrasts with the prevalent view of terminal differentiation of T cells, which is thought to lead to a dysfunctional or exhausted state. Although the precise mechanisms by which these cells support tissue and organismal health remain unclear, the mild cellular infiltration of the colon after depletion of IL-10^+^ T_reg_ cells raises the possibility that this subset acts on stromal or parenchymal cells. Importantly, given the dispensability of continuous T_reg_ production of IL-10 in controlling inflammation in adulthood, this essential function must be reliant on a spectrum of effector molecules, probably including CD39, CTLA-4, galectin-3 and granzyme B enriched expression in this T_reg_ subset. Together, our studies reveal that colonic terminally differentiated IL-10^+^ T_reg_ cells have an essential role in maintaining colonic health and do so by deploying a combination of effector mechanisms in addition to IL-10.

## Methods

### Mice

*Foxp3*^Thy1.1^, *Foxp3*^GFP-DTR^, *Foxp3*^CreER-GFP^ and *Il10*^fl^ mice have been previously described and were maintained in-house^8,33,70,71^. *Gt(ROSA)26Sor*^*LSL-YFP*^ and *Rorc*^fl^ have been previously described and were purchased from Jackson Laboratories^34,72^. *Tcra*^fl^ and *Maf*^fl^ have been previously described^55,73^. *Maf*^fl^ mice were provided by D. R. Littman and *Tcra*^fl^ mice were provided by M. Schmidt-Supprian. *Il10*^FM^ mice were generated by intercrossing *Foxp3*^Thy1.1^, *Gt(ROSA)26Sor*^*LSL-YFP*^ and *Il10*^tdTomato-CreER^ mice (see below) to homozygosity for each allele. Littermates were used in all experiments and were distributed among experimental groups evenly whenever possible, with different experimental groups co-housed. In experiments with different genotypes, all genotypes were represented in each litter analyzed. All mice were maintained at the Research Animal Resource Center for Memorial Sloan Kettering Cancer Center (MSKCC) and Weill Cornell Medicine under specific-pathogen-free conditions, with controlled humidity and temperature, a 12 h/12 h light/dark cycle and ad libitum access to diet (LabDiet 5053) and reverse-osmosis-filtered water. For studies in which treatments were given in drinking water, the same reverse-osmosis-filtered water was used as the vehicle. All studies were under protocol 08-10-023 and approved by the MSKCC Institute Institutional Animal Care and Use Committee. All animals used in this study had no previous history of experimentation and were naive at the time of analysis. Both sexes were used in all experiments unless otherwise noted, as no sex differences in IL-10 expression were detected.

### Generation of *Il10*^tdTomato-CreER^ and *Il10*^tdTomato-Cre^ mice

*Il10*^tdTomato-CreER^ mice were generated by insertion of a targeting construct into the *Il10* locus by homologous recombination in embryonic stem cells on the C57BL/6 background. The targeting construct was generated by inserting a sequence containing exons 2–5 of the *Il10* gene into a plasmid backbone containing a PGK promoter driving expression of diphtheria toxin A subunit followed by BGHpA sequence (modified PL452 plasmid). A SalI restriction enzyme site was simultaneously engineered into the *Il10* 3′ UTR between the stop codon and the polyadenylation site. The Clontech Infusion HD Cloning system was used to generate in the pUC19 plasmid backbone sequence encoding (in order from 5′ to 3′) encephalomyocarditis virus IRES; tdTomato; T2A self-cleaving peptide from *Thosea asigna* virus; Cre recombinase fused to the estrogen receptor ligand binding domain (CreER); followed by a FRT site-flanked PGK-Neomycin resistance gene (Neo)-BGHpA cassette. The IRES-tdTomato-T2A-CreERT2-FRT-Neo-BGHpA-FRT sequence was PCR-amplified and inserted into the SalI site in the *Il10* 3′ UTR in the modified PL452 backbone. The resulting plasmid was linearized with the restriction enzyme NotI before electroporation into embryonic stem cells. *Il10*^tdTomato-CreER^ mice were bred to *Gt(ROSA)26Sor*^*FLP1*^ mice (MSKCC Mouse Genetics Core) to excise the Neo cassette and backcrossed to C57BL/6 mice to remove the *Gt(ROSA)26Sor*^*FLP1*^ allele. *Il10*^tdTomato-Cre^ mice were generated in an identical manner except that the targeting vector contained a codon-optimized NLS-Cre encoding sequence after the T2A.

### Generation of *Foxp3*^LSL-DTR^ mice

*Foxp3*^LSL-DTR^ mice were generated by Biocytogen. First, a guide RNA targeting the 3′ UTR of the *Foxp3* gene was designed and validated (GGAAAGTTCACGAATGTACCA). Then, a targeting vector was constructed including 1,400 bp homology upstream and downstream of an SspI site in the *Foxp3* 3′ UTR. The following sequence was inserted into the SspI site: loxP-IRES-thy1.1-pA-loxP-IRES-DTR-eGFP. Cas9 protein, in-vitro-transcribed sgRNA and the targeting vector were then micro-injected into C57BL/6N zygotes. Founder pups were then bred and confirmed to have the proper integration by PCR and Southern blot analysis.

### Mouse treatments

For tamoxifen treatment, mice were gavaged with 8 mg tamoxifen dissolved in 200 μl corn oil (Sigma-Aldrich). Tamoxifen was dissolved by gentle agitation at 37 °C overnight. Aliquots were frozen (−80 °C) and thawed as needed throughout the experiments. We found that freezing and storing at −80 °C rather than −20 °C greatly reduced the tendency of the tamoxifen to precipitate when thawed. For *Il10*^iΔTCR^ experiments, mice were treated on days 0 or 11 and analyzed on day 21. For *Foxp3*^iΔIl10^ experiments, mice were treated on days 0 and 2 and analyzed on day 16. For *Il10*^iΔRorc^, *Il10*^iΔMaf^ and *Foxp3*^iΔIl10^ (long-term) experiments, mice were treated on days 0, 4, 11, 18, 25 and 32 and analyzed on day 35. DT was reconstituted in sterile PBS at 1 mg ml^−1^ and frozen at −80 °C in single-use aliquots. Aliquots were thawed and diluted in 995 μl PBS. For inactivated control (bDT), this 1 ml dilution was heated at 95–100 °C for 30 min. Both active and control DT were filtered through 0.22 μm syringe-driven filters. Mice were injected intraperitoneally with 200 μl of this dilution for the first two doses (1,000 ng DT) or with 200 μl of a 1:1 dilution with PBS for subsequent doses (500 ng DT), except for experiments depicted in Extended Data Fig. 9h–k, in which 1,000 ng DT was administered for each dose. Bleomycin was dissolved in PBS at a concentration of 5.7 U ml^−1^, sterile-filtered and frozen (−80 °C) in single-use aliquots. Aliquots were diluted with sterile PBS immediately before use. Mice were anesthetized with isofluorane (3% in O_2_, 3 l min^−1^; Covetrus), and 0.1 U bleomycin in 35 μl PBS was administered intranasally using a micropipette. Mice were exposed to isoflurane for at least 5 min before delivery of bleomycin, and the mouth was gently pressed shut during delivery to prevent swallowing. Bleomycin was given drop-wise, with pauses between drops to ensure inhalation. For antibiotic treatment, a solution of 1 g l^−1^ ampicillin sodium salt, 1 g l^−1^ kanamycin sulfate, 0.8 g l^−1^ vancomycin hydrochloride, 0.5 g l^−1^ metronidazole and 2.5 g l^−1^ sucralose (Splenda) was prepared in acidified, reverse-osmosed water and sterile-filtered. The control solution contained only sucralose but was otherwise treated similarly. Solutions were replaced every 7 days for the duration of the experiment. For chemically induced colitis (Fig. 1), 15 g of DSS salt (molecular weight, ~40,000) was dissolved in 50 ml distilled deionized water and then sterile-filtered. This solution was then diluted in acidified, reverse-osmosed water, resulting in a final concentration of 3% (w/v) DSS. Control groups received the same amount of sterile-filtered distilled deionized water diluted into acidified, reverse-osmosed water. For these experiments, female mice were used, as male mice proved to be highly sensitive to even lower concentrations of DSS^74^. For chemically induced colitis (Fig. 7 and Extended Data Fig. 6), 7.5 g of DSS (molecular weight, ~40,000) was dissolved in 50 ml distilled deionized water and then sterile-filtered. For *Foxp3*^CreER^*Il10*^fl/KO^ male mice (Extended Data Fig. 6c–e), 5 g of DSS was dissolved in 50 ml distilled water and then sterile-filtered. The stock solution was then diluted in 450 ml of acidified, reverse-osmosed water, resulting in a final concentration of 1.5% or 1% (w/v) DSS. The solution was replaced after 7 days. *Foxp3*^CreER^*Il10*^*f*l/KO^ mice were orally administered with two doses of 8 mg tamoxifen dissolved in 200 μl corn oil (Sigma-Aldrich) 48 h apart and, 7 days after the last dose of tamoxifen, they were administered 1.5% w/v (females) or 1% w/v (males) DSS in drinking water, a relatively low dose which causes minimal weight loss in control *Il10*^Cre^*Foxp3*^LSL-DTR^ mice treated with bDT.

### Cell isolation for flow cytometry

Mice were injected retro-orbitally with 1.5 μg anti-mouse CD45.2 (Brilliant Violet 510 conjugated; BioLegend, 109838) in 200 μl sterile PBS 3 min before the mice were killed to label and exclude blood-exposed cells. All centrifugations were performed at 800*g* for 3 min at 4 °C. SLOs were dissected and placed in 1 ml wash medium (RPMI 1640, 2% FBS, 10 mM HEPES buffer, 1% penicillin–streptomycin, 2 mM l-glutamine). Tissues were then mechanically disrupted with the back end of a syringe plunger and then passed through a 100 μm, 44% open area nylon mesh. For skin and lung, both ears and all lung lobes were collected. Ears were peeled apart to expose the dermis and cut into six total pieces. Tissues were then placed in 5 ml snap-cap tubes (Eppendorf, 0030119401) in 3 ml wash medium supplemented with 0.2 U ml^−1^ collagenase A, 5 mM calcium chloride and 1 U ml^−1^ DNase I along with three ¾ inch ceramic beads (MP Biomedicals, 116540424-CF). The tubes were shaken horizontally at 250 RPM for 45 min at 37 °C for the lung and for two rounds of 25 min for skin, replacing collagenase solution in between. Digested samples were then passed through a 70 μm strainer (Milltenyi Biotec, 130-095-823) and centrifuged to remove the collagenase solution. Lungs were then treated with ACK buffer (155 mM ammonium chloride, 10 mM potassium bicarbonate, 100 nM EDTA pH 7.2) to lyse red blood cells and then washed by centrifugation in 40% Percoll (ThermoFisher, 45-001-747) in wash medium to remove debris and enrich for lymphocytes. For colon, the cecum and large intestine were dissected and, after the removal of fat and the cecal patch, opened longitudinally and vigorously shaken in 1× PBS to remove luminal contents. Colon tissue was then cut into 1–2 cm pieces, placed in a 50 ml screw-cap tube with 25 ml wash medium supplemented with 5 mM EDTA and 1 mM dithiothreitol and shaken horizontally at 250 RPM for 15–20 min at 37 °C. After a 5 s vortex, epithelial and immune cells from the epithelial layer were removed by filtering the suspension through a tea strainer. The remaining tissue was placed back in 50 ml tubes, washed with 25 ml wash medium, strained again and replaced in 50 ml tubes. Then, 25 ml wash medium supplemented with 0.2 U ml^−1^ collagenase A, 4.8 mM calcium chloride and 1 U ml^−1^ DNase I was added along with four ¾ inch ceramic beads, and tissues were shaken horizontally at 250 RPM for 35 min at 37 °C. The suspension was then passed through a 100 μm strainer, centrifuged to remove debris and collagenase solution and then washed by centrifugation in 40% Percoll in wash medium. The small intestine was processed with the same steps as the colon, except that the pieces were cleaned by shaking in corn starch and then rinsed with PBS before EDTA treatment. All enzymatically digested samples were washed by centrifugation in 5 ml wash medium.

### Flow cytometry

To assess cytokine production after ex vivo restimulation, single-cell suspensions were incubated for 4 h at 37 °C with 5% CO_2_ in the presence of 50 ng ml^−1^ PMA and 500 ng ml^−1^ ionomycin with 1 μg ml^−1^ brefeldin A and 2 μM monensin to inhibit endoplasmic reticulum and Golgi transport. For flow cytometric analysis, cells were stained in 96-well V-bottom plates with antibodies and reagents used at concentrations indicated in Supplementary Table 1. All centrifugations were performed at 900*g* for 2 min at 4 °C. Staining with primary antibodies was carried out in 100 μl for 25 min at 4 °C in staining buffer (PBS, 0.2 % (w/v) BSA, 2 mM EDTA, 10 mM HEPES, 0.1% (w/v) NaN_3_). Cells were then washed with 200 μl PBS and then concurrently stained with Zombie NIR Fixable Viability dye and treated with 20 U ml^−1^ DNase I in DNase buffer (2.5 mM MgSO_4_, 0.5 mM CaCl_2_, 136.9 mM NaCl, 0.18 mM Na_2_HPO_4_, 5.36 mM KCl, 0.44 mM KH_2_PO_4_, 25 mM HEPES) for 10 min at room temperature (18–23 ºC). Cells were washed with 100 μl staining buffer, resuspended in 200 μl staining buffer and passed through a 100 μm nylon mesh. For cytokine staining, cells were fixed and permeabilized with BD Cytofix/Cytoperm per the manufacturer’s instructions. Intracellular antigens were stained overnight at 4 °C in 1× Perm/Wash buffer. Samples were then washed twice in 200 μl 1× Perm/Wash buffer, resuspending each time, resuspended in 200 μl staining buffer and passed through a 100 μm nylon mesh. All samples were acquired on an Aurora cytometer (Cytek Biosciences) and analyzed using FlowJo (v.10) (BD Biosciences).

### Histopathological analysis

Sections of colon (~1 cm) were fixed in 4% PFA for >48 h. Tissue embedding, sectioning and staining was carried out by Histowiz Inc. A blinded pathologist scored sections based on the Simplified Geboes Score rubric described in Supplementary Table 2 (ref. ^63^).

### Flow cytometric identification of immune cell populations

Generally, the following populations were identified with the associated markers (all immune cells were first gated as CD45^+^ and ZombieNIR^–^ and excluded doublets):

T_reg_ cells: CD90.2^+^CD5^+^SSC^lo^FSC^lo^TCRβ^+^TCRγδ^–^CD4^+^CD8α^–^Thy1.1^+^

T_H_ cells (CD44^hi^CD4^+^ cells): CD90.2^+^CD5^+^SSC^lo^FSC^lo^TCRβ^+^TCRγδ^–^CD4^+^CD8α^–^Thy1.1^–^CD44^+^

Macrophages: CD11b^+^CD64^+^CD90.2^–^CD19^–^NK1.1^–^Gr-1^–^SiglecF^–^Ly6C^–/lo^

Monocytes: CD11b^+^CD64^–/lo^CD90.2^–^CD19^–^NK1.1^–^Gr-1^–^SiglecF^–^Ly6C^hi^

Plasma cells: CD19^lo^CD44^hi^CD64^–^CD11b^–/lo^CD11c^–/lo^CD90.2^–^NK1.1^–^SiglecF^–^Gr-1^–^

Germinal center B cells: CD19^hi^CD44^lo^CD73^+^IgD^–^CD64^–^CD11b^–/lo^CD11c^–/lo^ CD90.2^–^NK1.1^–^SiglecF^–^Gr-1^–^

γδT cells: CD90.2^+^SSC^lo^FSC^lo^TCRβ^–^TCRγδ^+^

CD8^eff^ cells (CD44^hi^CD8^+^ T cells): CD90.2^+^SSC^lo^FSC^lo^TCRβ^+^TCRγδ^–^CD4^–^CD8α^+^CD44^hi^CD62L^–^

Natural killer cells: NK1.1^+^CD90.2^+/–^SSC^lo^FSC^lo^TCRβ^–^TCRγδ^–^CD19^–^CD64^–^CD11b^–^CD127^–^

Neutrophils: Gr-1^+^CD11b^+^CD64^–/lo^CD90.2^–^CD19^–^NK1.1^–^SiglecF^–^

Eosinophils: SiglecF^+^CD11b^+^CD64^–/lo^CD90.2^–^CD19^–^NK1.1^–^

### Cell sorting for sequencing

Cell isolation was performed as described above, except that samples were not washed with 40% Percoll. Staining was performed as described above, except the buffer contained 2 mM l-glutamine and did not contain NaN_3_, with the staining volume adjusted to 500 μl, washes adjusted to 5 ml and staining performed in 15 ml screw-cap tubes. ‘Hash-tag’ antibodies (1 μg; BioLegend, 155801, 155803, 155805, 155807) were added to the extracellular antigen stain for scRNA-seq sorting, and scRNA-seq sort samples were not treated with DNase I. Samples were resuspended in wash buffer supplemented with 5 mM EDTA for sorting. Samples were double sorted, with the first sort enriching for all Thy1.1^+^ T_reg_ cells and the second sort separating *Il10*^neg^, *Il10*^recent^ and *Il10*^stable^ or tdTomato^+^ versus tdTomato^–^ cells. For bulk RNA-seq, samples were sorted directly into Trizol-LS per the manufacturer’s instructions in 1.5 ml microcentrifuge tubes. For scRNA-seq, samples were sorted into PBS with 0.04% BSA (w/v) in 1.5 ml Protein LoBind tubes (Eppendorf, 0030108442). For ATAC–seq, samples were sorted into wash medium in 1.5 ml Protein LoBind tubes. All sorting was performed on an Aria II (BD Biosciences).

### Preparation of reference genome

The mm39 mouse genome assembly and NCBI RefSeq annotation information (GTF file) were downloaded from the UCSC Genome browser^75–78^. To account for the presence of the *Il10*^tdTomato-CreER^, *Foxp3*^Thy1.1^ and *Gt(ROSA)26Sor*^*LSL-YFP*^ targeted mutations, the corresponding sequences were inserted into the appropriate locations of the mm39 genome using the ‘reform’ script, creating the ‘reformed mm39’ genome^79^. The GTF file was modified to appropriately extend the *Il10*, *Foxp3* and *Gt(ROSA)26Sor* transcript and gene annotations and to shift all other affected annotations, resulting in a ‘reformed GTF’ using a custom R script, relying on the ‘GenomicRanges’ and ‘rtracklayer’ packages^80–82^. The reformed mm39 and reformed GTF were used for bulk RNA-seq and ATAC–seq alignment and analyses after generating a STAR genome index using STAR (v.2.7.3a)^83^.

### Bulk RNA-seq

A total of 5,000 cells were sorted per population per replicate for bulk RNA-seq, with each replicate pooled from two mice. RNA was extracted and libraries were prepared using SMARTer Stranded RNA-Seq Kits according to the manufacturer’s protocols (Takara) by the Integrated Genomics Operation (IGO) Core at MSKCC. Paired-end 50 bp reads (20–30 million per sample) were sequenced on an Illumina HiSeq 3000 by IGO.

### Bulk RNA-seq data processing

Samples were processed and aligned using Trimmomatic (v.0.39), STAR (v.2.7.3a) and Samtools (v.1.12), with the following steps, where *Sample* represents each *Il10*^neg^, *Il10*^recent^ or *Il10*^stable^ replicate^83–85^.

TrimmomaticPE *Sample*_R1.fastq.gz *Sample*_R2.fastq.gz -baseout *Sample*.fastq.gz ILLUMINACLIP:TruSeq3-PE.fa:2:30:10 LEADING:3 TRAILING:3 SLIDINGWINDOW:4:15 MINLEN:36

STAR–runThreadN 6–runMode alignReads–genomeLoad NoSharedMemory–readFilesCommand zcat–genomeDir mm39_100_RNA–readFilesIn *Sample*_1P.fastq.gz *Sample*_2P.fastq.gz–outFileNamePrefix *Sample*–outSAMtype BAM Unsorted–outBAMcompression 6–outFilterMultimapNmax 1–outFilterMismatchNoverLmax 0.06–outFilterMatchNminOverLread 0.35–outFilterMatchNmin 30–alignEndsType EndToEnd

samtools sort -@ 4 -n -o *Sample*.bam *Sample*Aligned.out.bam

samtools fixmate -@ 4 -rm *Sample*.bam *Sample*.fixmate.bam

samtools sort -@ 4 -o *Sample*.resort.bam *Sample*.fixmate.bam

samtools markdup -@ 4 -l 1500 -r -d 100 -s *Sample*.resort.bam *Sample*.duprm.bam

samtools index -@ 4 -b *Sample*.duprm.bam

This procedure resulted in the retention of all uniquely aligning reads, with PCR and optical duplicates removed, to be used for downstream analysis. Reads aligning to genes derived from the reformed GTF were then counted using a custom R script relying on the ‘GenomicAlignments’, ‘GenomicRanges’ and ‘GenomicFeatures’ packages with default counting parameters^81^. Differential expression analysis was carried out using the ‘DESeq2’ package, with the formula ‘~ Celltype + Replicate’, in which Celltype was either *Il10*^neg^, *Il10*^recent^ or *Il10*^stable^ and replicates were the separate samples from which each of the three populations were sorted^86^. Fragments per kilobase mapped (FPKM) normalized counts were extracted using the *fpkm* function of DESeq2. Differential expression analysis and statistical testing were performed for all pairwise comparisons of ‘Celltype’: *Il10*^neg^, *Il10*^recent^ and *Il10*^stable^. Differential expression analysis was performed on all genes, but genes with FPKM counts below the mean FPKM count of *Cd8a* (a gene functionally not expressed in T_reg_ cells), genes with zero counts in the majority of samples or genes corresponding to immunoglobulin or TCR variable, diversity or junction segments were eliminated from subsequent analyses. This process did not remove any significantly differentially expressed genes except immunoglobulin or TCR variable, diversity or junction segments, whose differential expression was not interpretable. *K*-means clustering was performed with R using per-gene *Z*-score-normalized counts of genes differentially expressed (adjusted *P* < 0.05) in any pairwise comparison between the three cell populations, with seven clusters chosen based on preliminary hierarchical clustering. TCR-activated and repressed genes were defined as genes that lost and gained expression in T_reg_ cells ablated of the *Tcra* gene^52^.

### scRNA-seq

Uniquely ‘hash-tagged’ samples from different tissues were pooled during sorting as separate tdTomato^+^ and tdTomato^–^ samples or total T_reg_ cells from large intestine lamina propria (LILP) and small intestine lamina propria (SILP) (Thy1.1^+^CD4^+^TCRβ^+^). The tdTomato^+^ sample had 48,000 cells (35,000 from LILP; 1,100 from lung; 11,000 from mesLN; and 900 from mediastinal lymph node (medLN)) and the tdTomato^–^ sample had 60,000 cells (30,000 from LILP; 10,000 each from lung, mesLN and medLN) or total 16,537 (Thy1.1^+^CD4^+^TCRβ^+^) T_reg_ cells from LILP and SILP. Samples were centrifuged and resuspended in 30 μl PBS with 0.04% BSA (w/v). Libraries were then prepared following the 10× Single Cell 3′ Reagent Kit (v.3) or 5′ kit with V(D)J enrichment for immune profiling (10× Genomics) following the manufacturer’s instructions, incorporating the BioLegend TotalSeq-A hash-tag oligonucleotide (HTO) protocol. Samples were sequenced on an Illumina NovaSeq platform by IGO.

### scRNA-seq processing

Reads from the tdTomato^+^ and tdTomato^–^ samples were processed, aligned to the mm39 genome and demultiplexed using Cell Ranger software (10× Genomics, v.7.0) with default parameters. Reads for the HTOs of the tdTomato^+^ and tdTomato^–^ samples were processed and demultiplexed using Cell Ranger software with default parameters. Samples were further processed and analyzed with a custom R script relying on the ‘Seurat’ (v.4) package^87^. First, genes detectable in fewer than 0.1% of cells were removed. Second, HTO identities (that is, LILP, lung, mesLN, medLN) were assigned using the *HTODemux* function, and cells without an unambiguous HTO identity or those determined to be a doublet were excluded^88^. Then, cells with mitochondrial genes accounting for >10% of gene counts, presumed to be dead or dying, as well as cells in the top or bottom 2% of total counts were eliminated. This latter cutoff was chosen based on a percentile rather than an arbitrary absolute value to account for different cell numbers and different median unique molecular identifier counts across the two samples. Afterwards, tdTomato^+^ and tdTomato^–^ samples were merged and analyzed together. First, the top 2,000 variable genes were identified and scaled. A principal component analysis (PCA) was performed on these genes and the top 30 principal components were used to assign *k*-nearest neighbors, generate a shared nearest-neighbor graph and then optimize the modularity function to determine clusters, at resolution = 0.5 (ref. ^89^). Based on these original clusters, a small population of cells dominated by high type I interferon signaling was excluded, and subsequent analyses were performed only on cells with LILP or mesLN HTO identities. The remaining cells had gene counts scaled again. A PCA was performed on the 3,000 most variable genes and the top 30 principal components were used to assign *k*-nearest neighbors, generate a shared nearest-neighbor graph and then optimize the modularity function to determine clusters, at resolution = 0.5. The shared nearest-neighbor graph was used as input for the Python-based algorithm ‘Harmony’ (600 iterations) to generate a two-dimensional force-directed layout for visualization^90^. The 30 principal components were used as input for the Python-based algorithm ‘Palantir’ to determine ‘pseudotime’ and ‘entropy’ values^42^. To reconcile scRNA-seq clusters and bulk RNA-seq populations, the ‘Seurat’ function *AddModuleScore* was used to assign scores for each bulk gene cluster to each cell. Mean scores for every cell cluster were calculated. For enrichment testing, the phyper function of R was used, in which *q* represents genes in a given bulk RNA-seq *k*-means cluster and also significantly over-expressed or under-expressed in a given scRNA-seq subset (combination of cell cluster and tdTomato^+^ or tdTomato^-^ identity); *m* represents genes in a given bulk RNA-seq *k*-means cluster; *n* represents all other genes with detectable expression in a given scRNA-seq subset; and *k* comprises all genes significantly over-expressed or under-expressed in a given scRNA-seq subset. For determining the relationship between IL-10-expressing T_reg_ cell subsets, we performed paired scRNA-seq and V(D)J-seq (Extended Data Figs. 9 and 10). Reads from the LILP and SILP samples were processed, aligned to the custom mm10 genome that contained *Il10*^tdTomato-CreER^, *Foxp3*^Thy1.1^ and *Gt(ROSA)26Sor*^*LSL-YFP*^ targeted mutations and demultiplexed using Cell Ranger software (10× Genomics) with default parameters. Reads for the HTOs of the LILP and SILP samples were processed and demultiplexed using Cell Ranger software with default parameters. Samples were further processed and analyzed with a custom Python script using the ‘scanpy’ package^91^. First, cells without an unambiguous HTO identity (LILP or SILP) or those determined to be a doublet were excluded. Next, cells with mitochondrial genes accounting for >5% of total genes were considered dead or dying and were removed. All genes encoding ribosomal proteins and genes expressed in less than 0.1% of cells were also removed. Then, using log-normalized data, the top 3,000 variable genes were identified to perform PCA. The top 100 principal components were used to generate a shared neighbors graph and a uniform manifold approximation and projection visualization. Initial clustering was performed using the ‘leiden’ function of scanpy with resolution = 1. Cells enriched in *Malat1* expression and with low library size were deemed as low-quality and were removed^92^. Gene counts of the remaining cells were scaled again, and a uniform manifold approximation and projection embedding was generated after calculating a PCA with the top 3,000 variable genes and creating a neighbors graph with 100 principal components. Unsupervised clustering was performed using resolution = 0.75. Bulk RNA-seq gene cluster (bI–bIV) (Fig. 2c) enrichment score for each annotated scRNA-seq T_reg_ cell cluster was calculated using the ‘score_genes’ function within scanpy. Differential gene expression analysis was performed using the Python-based ‘rpy2’ and R-based ‘MAST’ packages. Up to the top 50 differentially expressed genes (adjusted *P* < 0.05 and log_10_FC > 0.5) by either *Gata3*^+^ *Il10*^+^ or *Rorc*^+^ *Il10*^+^ T_reg_ cells from both tissues were plotted. To determine the extent of transcriptional similarity between and predict the developmental trajectory of annotated LILP or SILP T_reg_ cell clusters, the Python-based ‘PAGA’^93^ analysis was performed using distances computed on a diffusion map. To this end, after removing TCR-related genes, a diffusion map was created using the ‘diffmap’ function of scanpy and a PAGA map was generated using the ‘paga’ function of scanpy. ‘Pseudotime’ and ‘entropy’ values were calculated using the ‘Palantir’ algorithm with 30 principal components. Clonotypes with identical nucleotide-level CDR3 regions (ranging from 1–3 matching chains) were called using the built-in Cell Ranger enclone software, with 4,693 out of 7,868 cells (~60%) that had passed upstream RNA-level quality control being assigned to a clone. To measure clonal relatedness between phenotypic clusters, we computed the Jaccard overlap, defined as:

$$J({C}_{1},{C}_{2})=\,\frac{|{C}_{1}\cap {C}_{2}|}{|{C}_{1}|+|{C}_{2}|-|{C}_{1}\cap {C}_{2}|}$$

where ${C}_{1}$ is the set of clonotypes belonging to phenotype 1 and ${C}_{2}$ is the set of clonotypes belonging to phenotype 2. We computed both the pooled overlap and the mouse-level overlaps. To measure the reproducibility of this clonal structure, we measured the correlation between the overlap matrices of pairs of mice. This correlation was quantitatively probed using the Mantel matrix permutation test^94^, which takes as a null hypothesis that any two overlapping matrices are uncorrelated. Visualizations for paired scRNA-seq and V(D)J-seq were generated using the Python-based ‘matplotlib’ package.

### ATAC–seq

A total of 40,000 cells were sorted per population per replicate for ATAC–seq, with replicates one and two originating from a single mouse each and replicate three representing two pooled mice. ATAC–seq libraries were prepared as previously described, with some modifications^95^. Cells were pelleted in a fixed rotor benchtop centrifuge at 500*g* for 5 min at 4 °C. Cells were then washed in 1 ml cold PBS and pelleted again. The supernatant was aspirated and the cells were resuspended in 50 µl ice-cold cell lysis buffer (10 mM Tris-Cl pH 7.4, 10 mM NaCl, 3 mM MgCl_2,_ 0.1% NP-40) to disrupt plasma membranes. Nuclei were pelleted at 1,000*g* for 10 min. The supernatant was aspirated and the nuclei were resuspended in 40 µl of transposition reaction mixture (Illumina Tagment Kit: 20 µl TD buffer; 2 µl TDE1; 18 µl ddH_2_O). Samples were incubated in a ThermoMixer at 1,100 RPM for 45 min at 42 °C. DNA was then purified using a MinElute Reaction Cleanup Kit, according to the manufacturer’s instructions. DNA was eluted in 10 µl buffer EB. Libraries were then barcoded and amplified with NEBNext High-Fidelity Master Mix and primers described in a previous publication^96^ (50 µl reaction with 10 µl DNA and 2.5 µl of 25 µM primers; one cycle of 5 min at 72 °C, 30 s at 98 °C; five cycles of 10 s at 98 °C, 20 s at 63 °C, 1 min at 72 °C). A qPCR analysis on the product determined that an additional seven cycles (10 s at 98 °C, 20 s at 63 °C, 1 min at 72 °C) were required. The library was purified and size-selected with AMPure XP beads: 45 µl of PCR product was incubated with 18 µl beads and the supernatant was collected (beads bound larger than ~2,000 bp fragments). The supernatant (63 µl) was then incubated with an additional 63 µl of beads for 5 min, the supernatant was removed, the beads were washed twice with 75% ethanol and DNA was eluted into 50 µl H_2_O by incubating for 2 min. Samples were quality-control-checked and quantified on an Agilent BioAnalyzer by IGO. Paired-end 50 bp reads, 20–30 million per sample, were sequenced on an Illumina HiSeq 3000 by IGO.

### ATAC–seq data processing

Samples were processed and aligned using Trimmomatic (v.0.39), STAR (v.2.7.3a) and Samtools (v.1.12) with the following steps, where *Sample* represents each *Il10*^neg^, *Il10*^recent^ or *Il10*^stable^ replicate:

TrimmomaticPE *Sample*_R1.fastq.gz *Sample*_R2.fastq.gz -baseout *Sample*.fastq.gz ILLUMINACLIP:TruSeq3-PE.fa:2:30:10 LEADING:3 TRAILING:3 SLIDINGWINDOW:4:15 MINLEN:36

STAR–runThreadN 6–runMode alignReads–genomeLoad NoSharedMemory–readFilesCommand zcat–genomeDir mm39_100–readFilesIn *Sample*_1P.fastq.gz *Sample*_2P.fastq.gz–outFileNamePrefix *Sample*–outSAMtype BAM Unsorted–outBAMcompression 6–outFilterMultimapNmax 1–outFilterMismatchNoverLmax 0.06–outFilterMatchNminOverLread 0.35–outFilterMatchNmin 30–alignIntronMax 1–alignEndsType Local

samtools sort -@ 4 -n -o *Sample*.bam *Sample*Aligned.out.bam

samtools fixmate -@ 4 -rm *Sample*.bam *Sample*.fixmate.bam

samtools sort -@ 4 -o *Sample*.resort.bam *Sample*.fixmate.bam

samtools markdup -@ 4 -l 1500 -r -d 100 -s *Sample*.resort.bam *Sample*.duprm.bam

samtools sort -@ 4 -n -o *Sample*.byname.bam *Sample*.duprm.bam

samtools index -@ 4 -b *Sample*.duprm.bam

This procedure resulted in the retention of all uniquely aligning reads, with PCR and optical duplicates removed, to be used for downstream analysis. Then, peaks were called across the three replicates of each cell population individually using Genrich (v.0.5), with the following command (in which *Celltype* stands in for each cell population, *Sample* (r1–r3) represents the three replicates and X is 0.002% of the mean number of uniquely aligned reads for each cell population)^97^:

Genrich -t *Sample*_r1.byname.bam,*Sample*_r2.byname.bam,*Sample*_r3.byname.bam -o./Gen_out/Peak/Celltype.narrowPeak -j -d 25 -g 5 -v -q 0.01 -a X.

Peak atlases for each population were then concatenated, sorted and clustered using bedtools (v.2.27.1) to identify overlapping peaks with the following command^90^:

bedtools cluster -d -1 -i combined_sort.narrowPeak > clustered.narrowPeak

A custom R script was then used to merge the atlases according to the following principles. If all the peaks in a cluster entirely overlapped, defined as all peak summits falling within the maximal start and minimal end positions of the cluster, the merged peak was defined as the mean start, summit and end of all peaks in that cluster. This was the case for ~82% of all peaks. In the other cases, clusters had multiple distinct summits. These clusters were divided into distinct peaks, with one for each distinct summit, and the boundaries were defined by the most proximal downstream and upstream start and end positions within the cluster. Peaks assigned to regions of the assembly not corresponding to any chromosome and peaks with a width >3,500 bases were eliminated. The *getfasta* function of bedtools and a custom R script were used to identify and eliminate peaks with >70% repetitive elements. The final combined atlas contained 70,323 peaks. The R packages ‘GenomicRanges’ and ‘ChIPpeakAnno’ were used to assign peaks to the closest gene according to the following principles^81,98,99^. Peaks 2,000 bases upstream or 500 bases downstream of a transcription start site were considered ‘promoter’ peaks. Non-promoter peaks within the body of a gene were considered ‘intragenic’ peaks. Peaks 100,000 bases up or downstream of a gene body were considered ‘intergenic’. All other peaks were not assigned to any specific gene. Differential accessibility analysis was carried out using the ‘DESeq2’ package, with the formula ‘~ Celltype + Replicate’, where Celltype is either *Il10*^neg^, *Il10*^recent^ or *Il10*^stable^ and replicates are the separate samples from which each of the three populations were sorted. Differential accessibility analysis and statistical testing were performed for all pairwise comparisons of ‘Celltype’: *Il10*^neg^, *Il10*^recent^ and *Il10*^stable^.

### Motif identification and model generation

Motif discovery in peaks and model creation were carried out as previously described^44^, with some modifications. Motifs for all mouse TFs were downloaded from CisBP (v.2.00)^100,101^. TFs with mean FPKM > 1 in any cell population were used for further analysis. This resulted in 319 motifs for 188 TF-encoding genes. The ‘AME’ software from the MEME suite (v.5.3.0) was used to identify which of these motifs were enriched in the sequences corresponding to the combined peak atlas^102,103^. At this stage, the best motif for each TF-encoding gene, defined as being detected in the highest fraction of peaks, was chosen for further analysis. Then, the ‘Tomtom’ software from the MEME suite was used to determine closely related motifs^104^. A custom R script was used to group motifs according to the following principles. Motifs were grouped if their ‘Tomtom’ E-value was <0.00001 and if they were in the same protein family (according to CisBP). Then, groups were merged based on overlapping members until each gene belonged to at most one group. This resulted in 142 motif groups. Finally, motifs not significantly enriched in the peak atlas (‘AME’-adjusted *P* < 0.01) were excluded. This resulted in 76 TF-encoding genes organized into 58 groups. The ‘FIMO’ software from the MEME suite was used to identify individual instances (*P* < 0.0001) of each of the 76 motifs across the entire peak atlas^105^. Motif families occurring in fewer than 2% of peaks were eliminated. This resulted in a final set of 57 motifs within 40 groups. Finally, a peak-by-motif matrix was generated, in which 1 indicated at least one instance of a motif belonging to that family and 0 indicated no motif.

The ‘ridge’ package in R was used to fit a linear ridge regression for the log_2_FC in accessibility at each peak as a function of the peak-by-motif matrix^45,46^. This package applies an algorithm for semi-automatically determining the optimal ridge parameter(s) to use to maximize model performance and also performs significance testing using the method of Cule^45^. This was done separately for the *Il10*^stable^ vs *Il10*^recent^ log_2_FC (the svr model) and *Il10*^stable^ vs *Il10*^neg^ log_2_FC (the svn model). Motif families with significant coefficients in the models (*P* < 0.001) were used in subsequent analyses. At the same time, linear ridge regressions were fit as above, except with each motif individually removed from the matrix, generating a series of ‘zeroed-out’ models. Then, for sets of peaks of interest (for example, those associated with a specific cluster of genes), the correlations between the actual log_2_FC and the log_2_FC predicted by the svr or svn model as well as the correlations between the actual log_2_FC and the log_2_FC predicted by the ‘zeroed-out’ svr or svn models were determined. Decreased correlation for the ‘zeroed-out’ model was assumed to be indicative of the ‘zeroed-out’ motif disproportionately contributing to the model’s predictiveness at those specific peaks, and therefore potentially regulating accessibility.

### Plotting RNA-seq and ATAC–seq tracks

The UCSC utility ‘faCount’ was used to determine the effective genome size of the ‘reformed mm39’ genome^106^. The bamCoverage function of deeptools (v.3.5.1) was used to generate ‘bigwig’ files for ATAC–seq and RNA-seq tracks, with the following command^107^:

bamCoverage -b *Sample*.duprm.bam–effectiveGenomeSize *x* -bs 1–maxFragmentLength *y*–scaleFactor *z* -o *Sample*.normdt.bw, where *Sample* represents each *Il10*^neg^, *Il10*^recent^ or *Il10*^stable^ replicate; *x* is the effective genome size as defined above; *y* is the equivalent value determined by STAR during alignment ((*2ˆwinBinNbits*)*winAnchorDistNbins) for ATAC–seq or the default for RNA-seq; and *z* is the inverse of the size factors determined by DESeq2.

### TCR deletion RNA-seq

A total of 5,000 cells were sorted per population per replicate for bulk RNA-seq. RNA was extracted and libraries were prepared using SMARTer Stranded RNA-Seq Kits according to the manufacturer’s protocols (Takara) by the IGO Core at MSKCC. Paired-end 50 bp reads (20–30 million per sample) were sequenced on an Illumina HiSeq 3000 by IGO.

### TCR deletion RNA-seq data processing

Samples were processed and aligned using Trimmomatic (v0.39), STAR (v.2.7.3a) and samtools (v.1.12), with the following steps (in which *Sample* represents each d10 or d21 TCR^+^ or TCR^–^ replicate):

TrimmomaticPE *Sample*_R1.fastq.gz *Sample*_R2.fastq.gz -baseout *Sample*.fastq.gz ILLUMINACLIP:TruSeq3-PE.fa:2:30:10 LEADING:3 TRAILING:3 SLIDINGWINDOW:4:15 MINLEN:36

STAR–runThreadN 6–runMode alignReads–genomeLoad NoSharedMemory–readFilesCommand zcat–genomeDir mm39_100_RNA–readFilesIn *Sample*_1P.fastq.gz *Sample*_2P.fastq.gz–outFileNamePrefix *Sample*–outSAMtype BAM Unsorted–outBAMcompression 6–outFilterMultimapNmax 1–outFilterMismatchNoverLmax 0.06–outFilterMatchNminOverLread 0.35–outFilterMatchNmin 60–alignEndsType EndToEnd

samtools sort -@ 4 -n -o *Sample*.bam *Sample*Aligned.out.bam

samtools fixmate -@ 4 -rm *Sample*.bam *Sample*.fixmate.bam

samtools sort -@ 4 -o *Sample*.resort.bam *Sample*.fixmate.bam

samtools markdup -@ 4 -l 1500 -r -d 100 -s *Sample*.resort.bam *Sample*.duprm.bam

samtools index -@ 4 -b *Sample*.duprm.bam

This procedure resulted in the retention of all uniquely aligning reads, with PCR and optical duplicates removed, to be used for downstream analysis. Reads aligning to genes derived from the reformed GTF were then counted using a custom R script relying on the ‘GenomicAlignments’, ‘GenomicRanges’ and ‘GenomicFeatures’ packages with default counting parameters. Differential expression analysis was carried out using the ‘DESeq2’ package, with the formula ‘~Conditon’, in which Condition is each unique combination of timepoint and whether cells lost or retained cell surface TCR. FPKM-normalized counts were extracted using the *fpkm* function of DESeq2. Differential expression analysis and statistical testing were performed for TCR^+^ versus TCR^−^ replicates at each timepoint. Differential expression analysis was performed on all genes, but genes with FPKM counts below the mean FPKM count of *Cd8a* (a gene functionally not expressed in T_reg_ cells), genes with zero counts in the majority of samples or genes corresponding to immunoglobulin or TCR variable, diversity or junction segments were eliminated for subsequent analyses. This process did not remove any significantly differentially expressed genes, except immunoglobulin or TCR variable, diversity or junction segments, whose differential expression was not interpretable.

### In vitro transduction

To produce virus, HEK293T cells (ATCC CRL-3216) grown to 70% confluency in complete DMEM (10% FBS) were transfected with 25 µg modified MIGR1 vector, 20 µg pCL-Eco helper plasmid, and 135 µl Fugene transfection reagent (Promega). The vector structure was as follows: 5′ LTR – MESV psi – gag – lox71 (sense) – IRES-mAmetrine (antisense) – Maf/Rorc/empty (antisense) – lox66 – 3′ LTR. This enabled Cre-mediated swapping of the Maf/Rorc/empty – IRES – mAmetrine reading frame into the translated, sense orientation. Then, 48 h after transfection, the virus was concentrated using RetroX concentrator reagent (Takara). Meanwhile, T_reg_ cells were sorted as CD4^+^TCRβ^+^Thy1.1^+^tdTomato^−^ and cultured in complete RPMI (10% FBS) containing 1000 U ml^−1^ IL-2, on anti-CD3/CD28 (2 µg ml^−1^) pre-coated plates. At 24 h post-culture, cells were ‘spin-fected’ at 1,250*g* for 90 min at 32 °C in complete RPMI containing concentrated virus, 8 µg ml^−1^ polybrene and 1,000 U ml^−1^ IL-2; 3 days later, cells were isolated and analyzed by flow cytometry.

### Statistical analysis and data plotting

All statistical tests were carried out in R using base R or the indicated packages, or by the indicated software or Python algorithms. No statistical methods were used to pre-determine sample sizes but sizes are limited by experimental feasibility and are typical for the field (see previous works^10,17,23,25,27^). Data distribution was assumed to be normal but this was not formally tested. Mice were randomly assigned to treatments whenever possible, except to ensure that equal numbers of mice were allocated to all relevant groups. Data collection was randomized whenever possible. Data collection and analysis were not performed blind to the conditions of the experiments. Plots were generated in R, using the ggplot2, patchwork, RColorBrewer, ggrepel and Seurat packages^87,108–111^. Inkscape (v.1.0.2)^112^ was used to modify and assemble plots for presentation.

### Reporting summary

Further information on research design is available in the Nature Portfolio Reporting Summary linked to this article.

## Online content

Any methods, additional references, Nature Portfolio reporting summaries, source data, extended data, supplementary information, acknowledgements, peer review information; details of author contributions and competing interests; and statements of data and code availability are available at 10.1038/s41590-024-02075-6.

## Supplementary information

Supplementary Information Supplementary Figs. 1–10, Supplementary Tables 1 and 2.

Reporting Summary

## Source data

Source Data Fig. 1 Statistical Source Data.

Source Data Fig. 2 Statistical Source Data.

Source Data Fig. 3 Statistical Source Data.

Source Data Fig. 4 Statistical Source Data.

Source Data Fig. 5 Statistical Source Data.

Source Data Fig. 6 Statistical Source Data.

Source Data Fig. 7 Statistical Source Data.

Source Data Fig. 8 Statistical Source Data.

Source Data Extended Data Fig. 1 Statistical Source Data.

Source Data Extended Data Fig. 2 Statistical Source Data.

Source Data Extended Data Fig. 3 Statistical Source Data.

Source Data Extended Data Fig. 4 Statistical Source Data.

Source Data Extended Data Fig. 5 Statistical Source Data.

Source Data Extended Data Fig. 6 Statistical Source Data.

Source Data Extended Data Fig. 7 Statistical Source Data.

Source Data Extended Data Fig. 8 Statistical Source Data.

Source Data Extended Data Fig. 9 Statistical Source Data.

Source Data Extended Data Fig. 10 Statistical Source Data.

## Data Availability

Bulk RNA-seq, ATAC–seq, scRNA-seq and TCR-seq data have been deposited in the Gene Expression Omnibus (GEO) under accession code GSE207969. Source data are provided with this paper.

## Code availability

All custom code used for the analysis of data and generation of plots is available upon request (sdikiy@scripps.edu).
