# Supplementary material for: Terminal differentiation and persistence of effector regulatory T cells essential for preventing intestinal inflammation
Source: Nat Immunol. 2025 Feb 4;26(3):444–58. doi: 10.1038/s41590-024-02075-6 (PMC11876075; doi:10.1038/s41590-024-02075-6)
Supplement: Supplementary file 1 — Supplementary Figs. 1–10, Supplementary Tables 1 and 2. [file 41590_2024_2075_MOESM1_ESM.pdf]

nature immunology

Supplementary information https://doi.org/10.1038/s41590-024-02075-6

Terminal differentiation and persistence of effector regulatory T cells essential for preventing intestinal inflammation

In the format provided by the authors and unedited

Supplementary Figure 1 - Gating strategy for Thelper and Treg cells

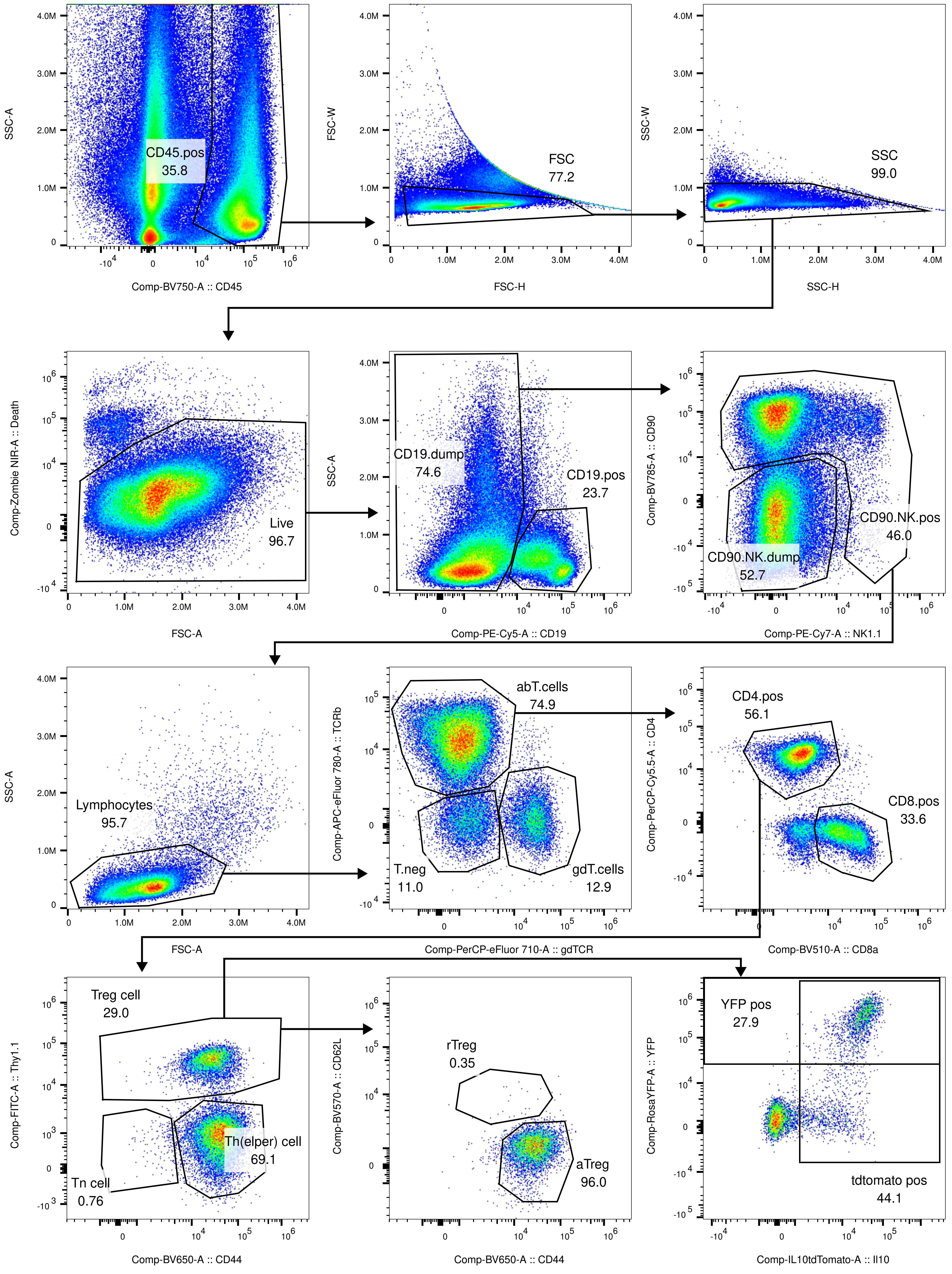

Supplementary Figure 2 - Gating strategy for Macrophages and monocytes

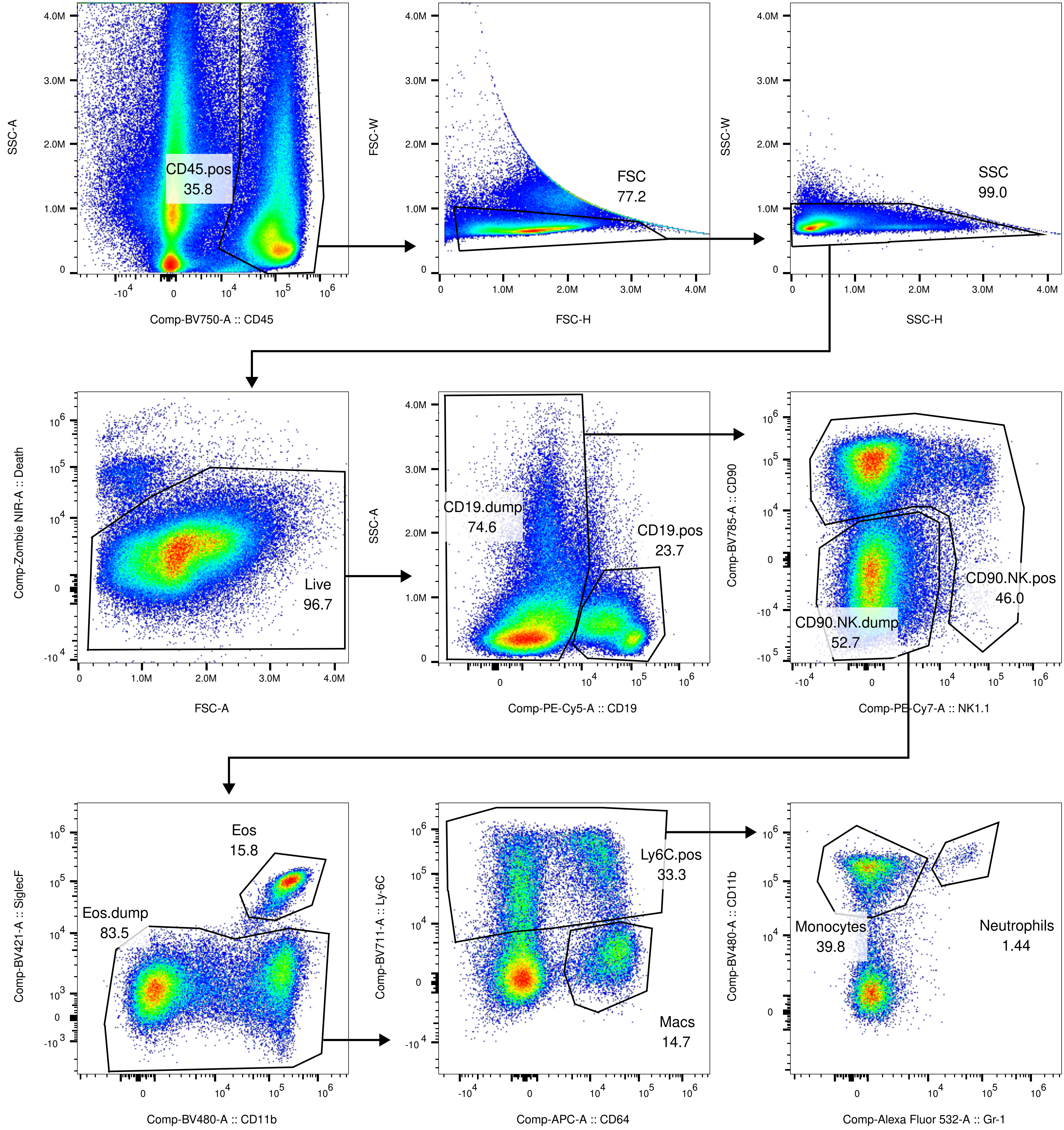

Supplementary Figure 3 - Gating strategy for B cells

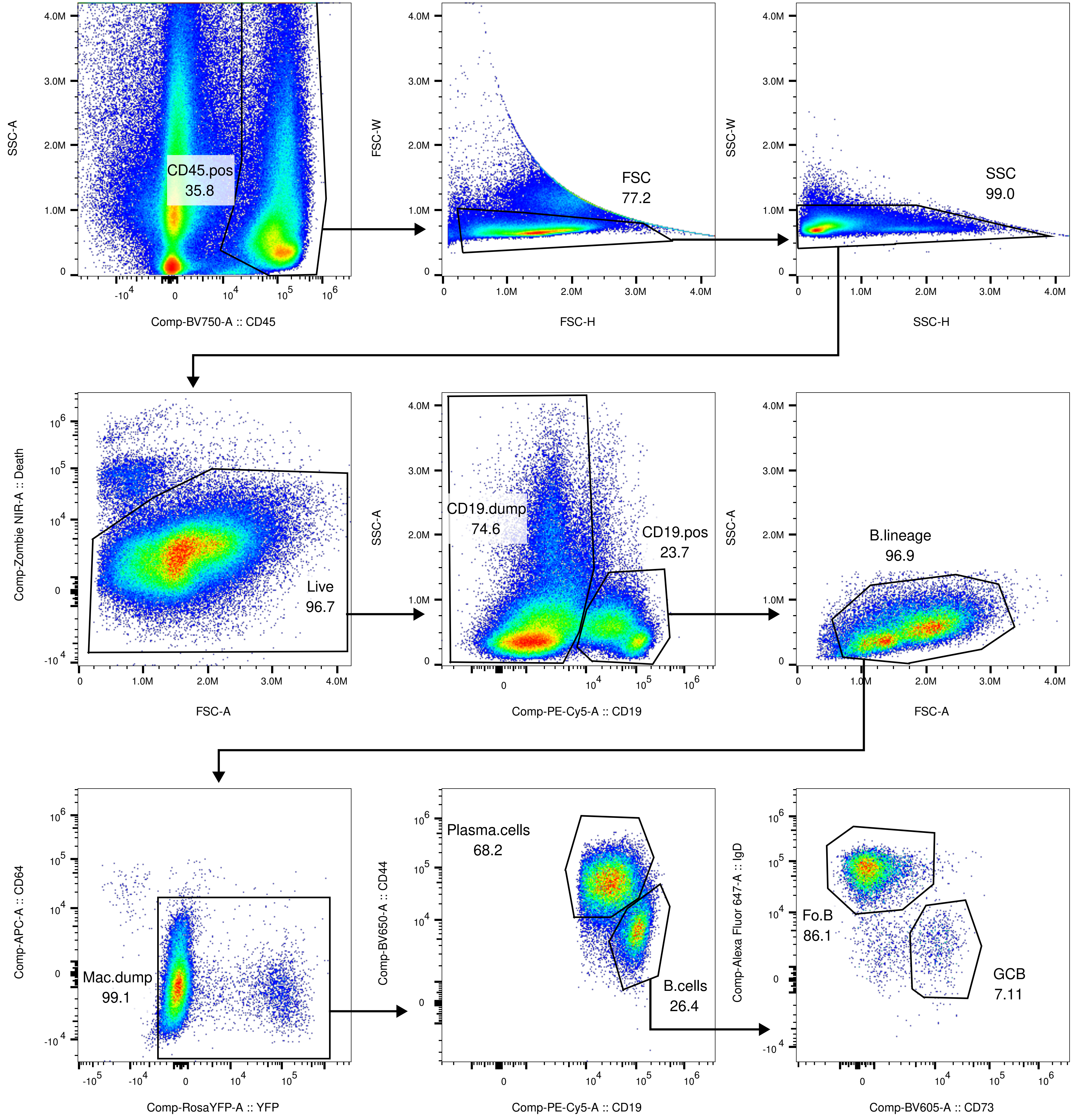

Supplementary Figure 4 - Gating strategy for Treg cells in Il10$^{i\Delta Trac}$ mie

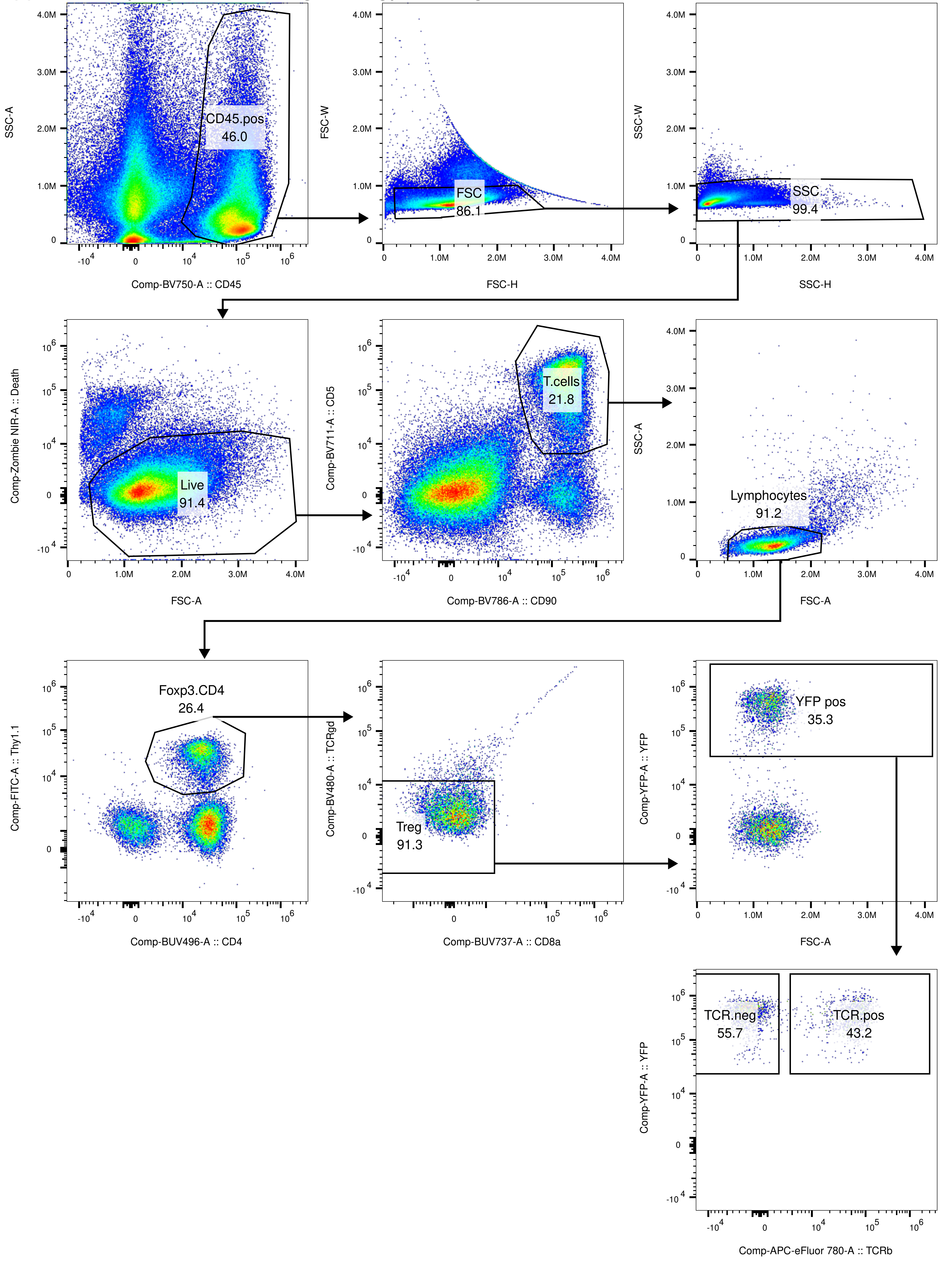

Supplementary Figure 5 - Gating strategy for Treg cells in Foxp3^{CreER} mice

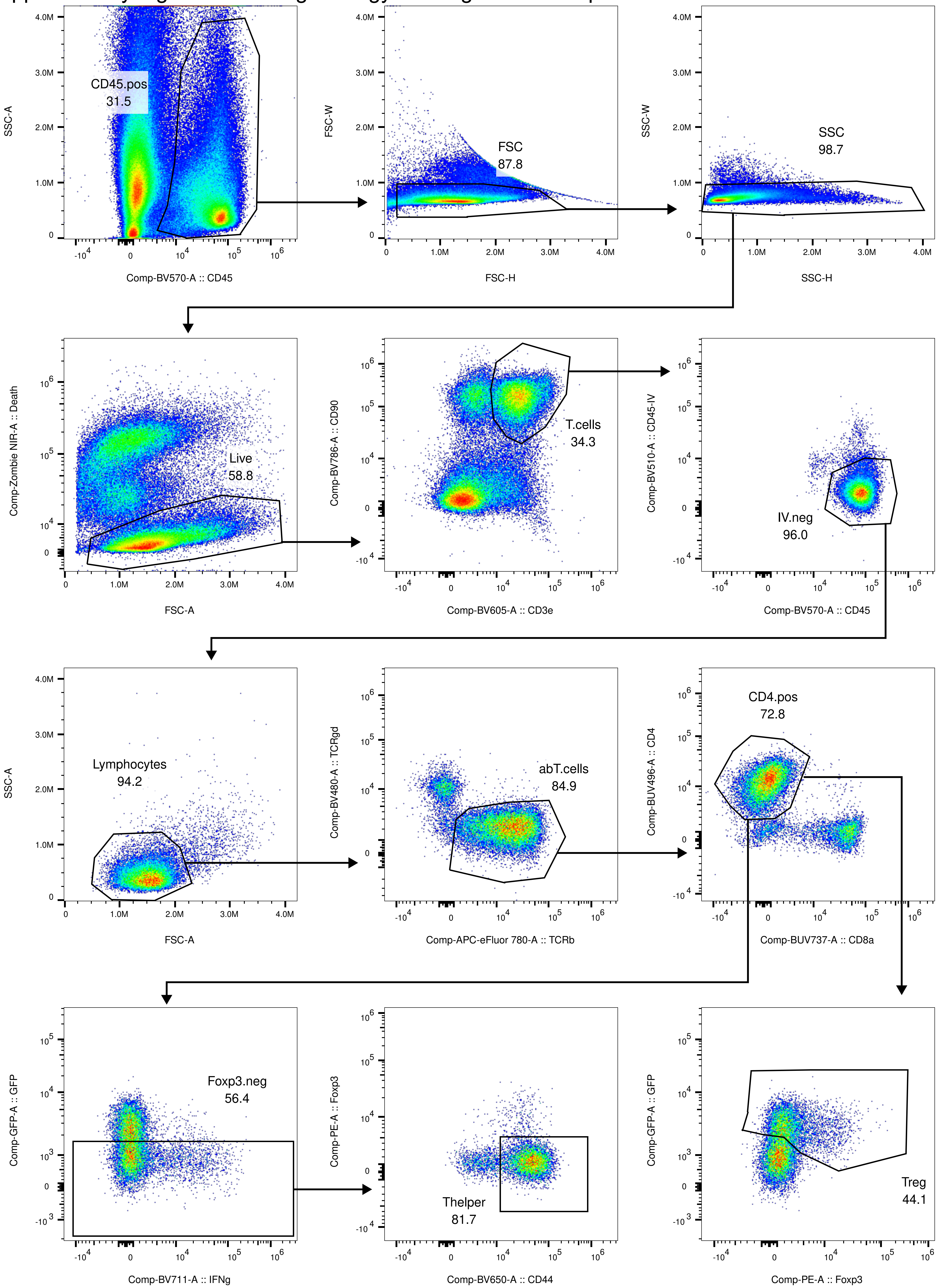

Supplementary Figure 6 - Gating strategy for GFP in Foxp3$^{LSL-DTR}$ mice

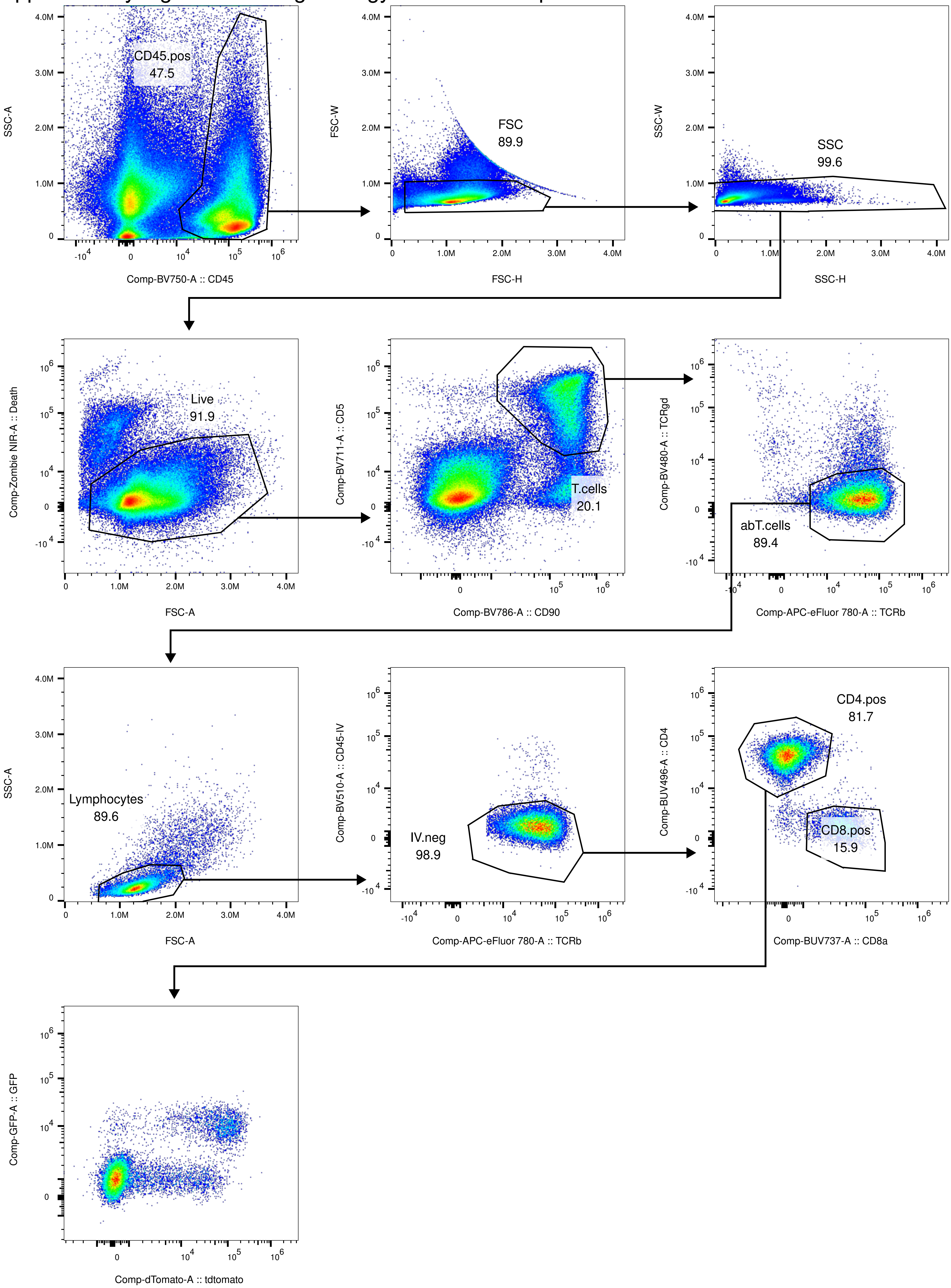

Supplementary Figure 7 - Gating strategy for Foxp3 in Foxp3$^{LSL-DTR}$ mice

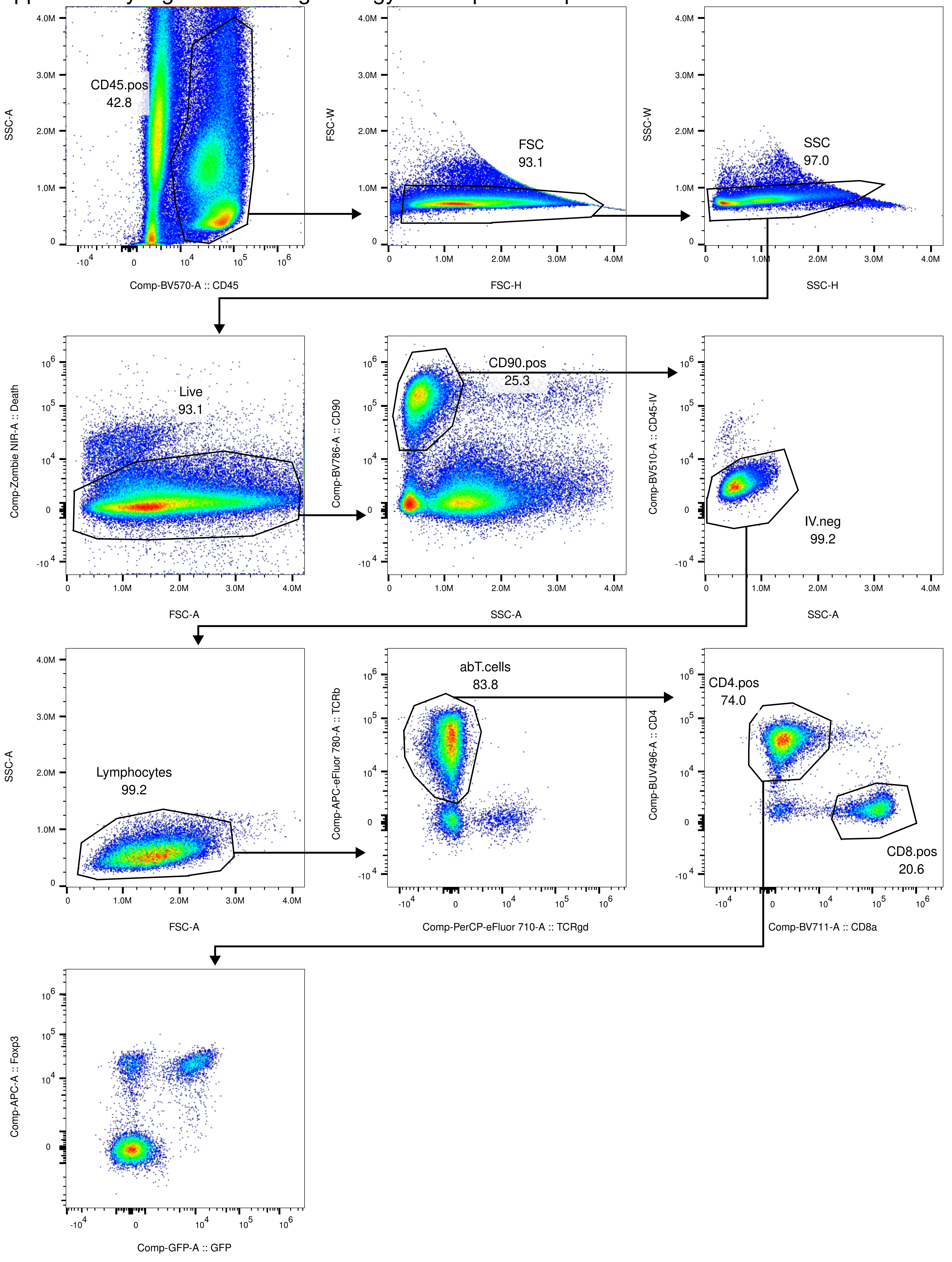

Supplementary Figure 8 - Gating strategy for Neutrophils and Eosinophils

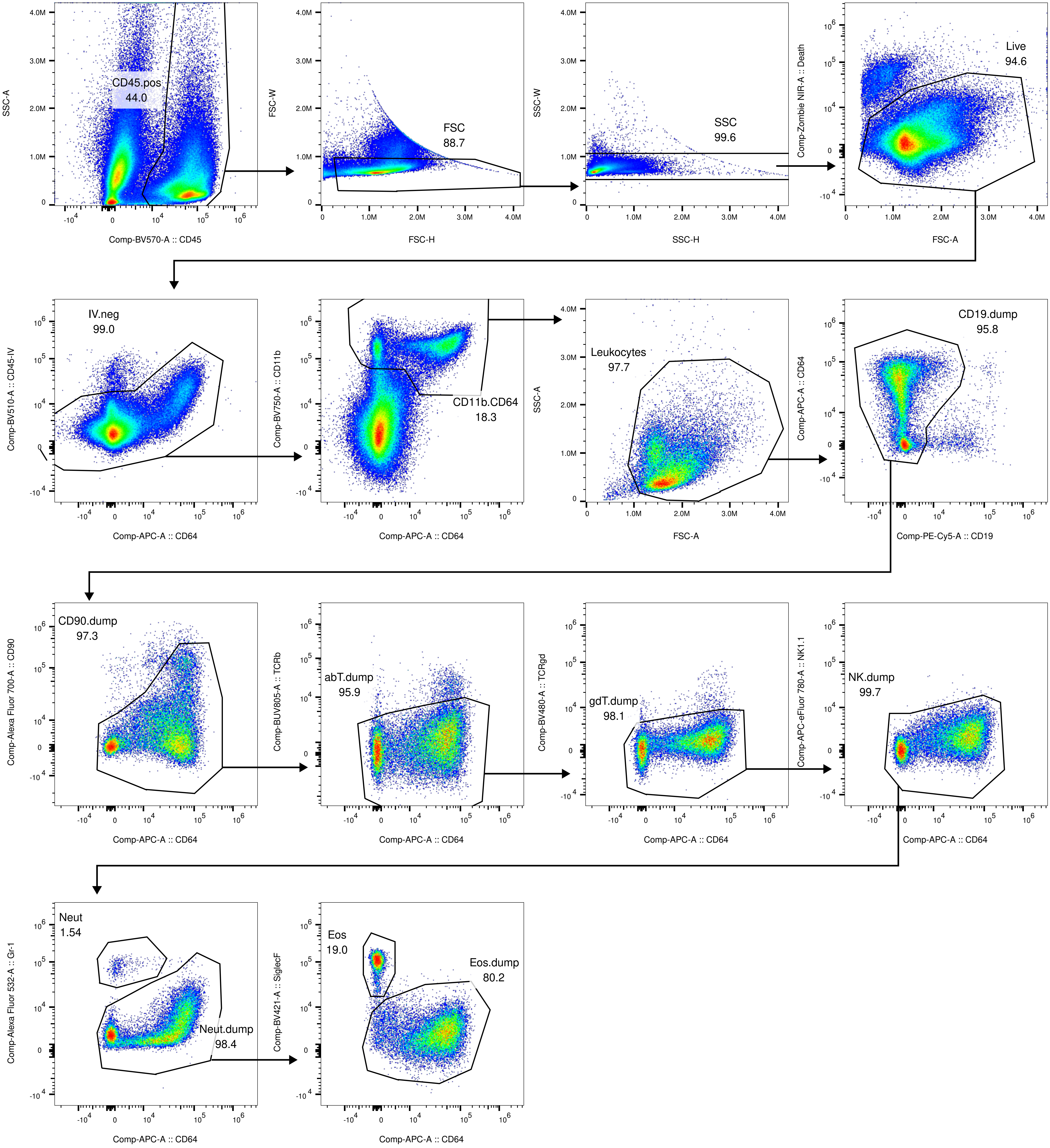

Supplementary Figure 9 - Gating strategy for ILCs and NK cells

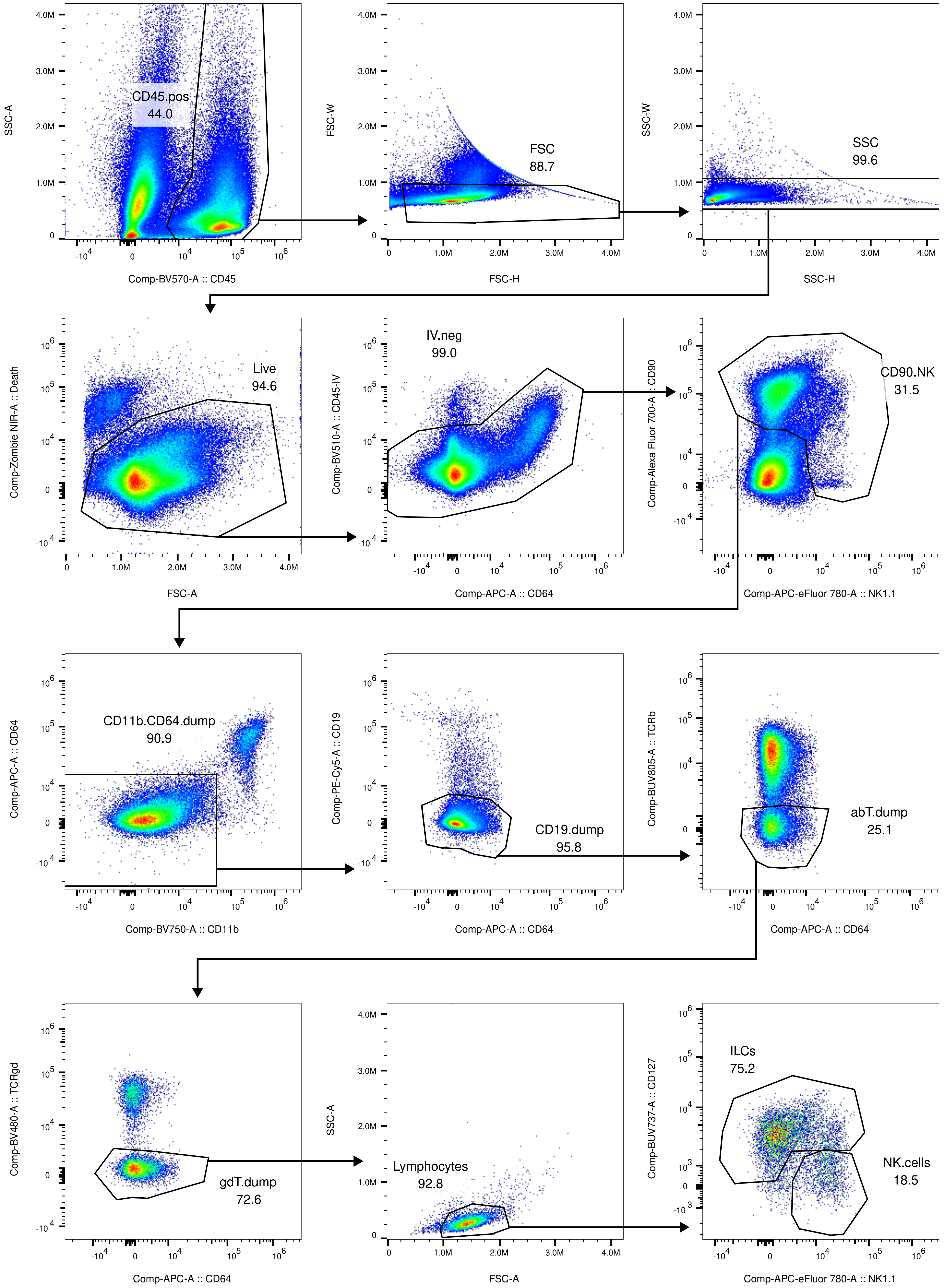

Supplementary Figure 10 - Gating strategy for Treg cells from $Il10^{FM}$ mice for sort

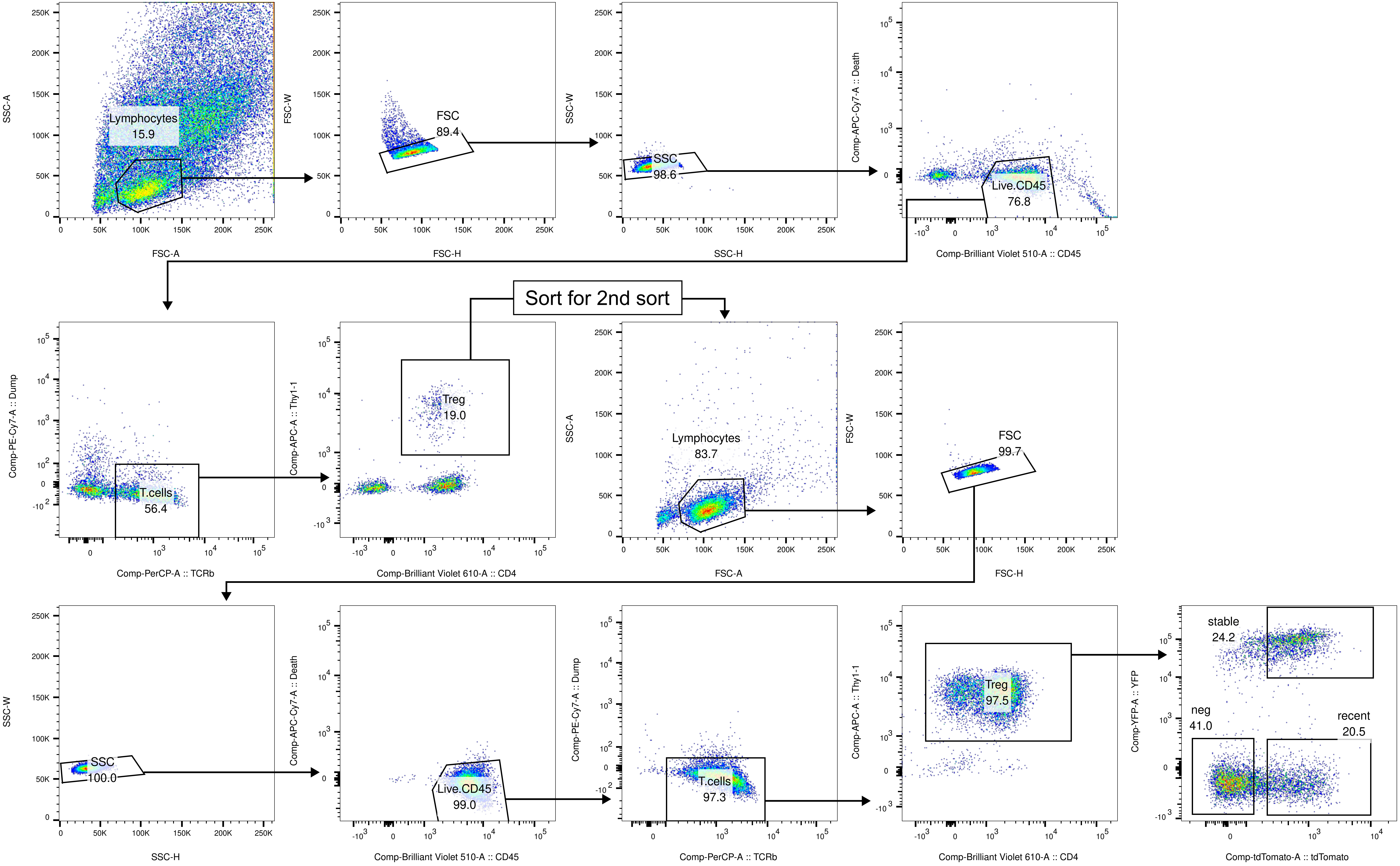

Table S1. Antibody usage and validation information, including other flow cytometry reagents

Antigen	Fluorophore	Dilution (1/x)	Isotype	Clone	Company	Catalog No.	Validation
CD19	Brilliant UltraViolet 395	400	Rat IgG2a, κ	1D3	BD Biosciences	563557	Tested on mouse splenocytes vs isotype control (manufacturer)
CD25	Brilliant UltraViolet 395	300	Rat IgG1, λ	PC61	BD Biosciences	564022	Tested on stimulated mouse splenocytes vs isotype control (manufacturer)
CD45R (B220)	Brilliant UltraViolet 496	400	Rat IgG2a, κ	RA3-6B2	BD Biosciences	564662	Tested on mouse splenocytes vs isotype control (manufacturer)
CD4	Brilliant UltraViolet 496	400	Rat IgG2b, κ	GK1.5	BD Biosciences	564667	Tested on mouse splenocytes vs isotype control (manufacturer)
CD62L	Brilliant UltraViolet 737	600	Rat IgG2a, κ	MEL-14	BD Biosciences	565213	Tested on mouse splenocytes vs isotype control (manufacturer)
CD8α	Brilliant UltraViolet 737	500	Rat IgG2a, κ	53-6.7	BD Biosciences	564297	Tested on mouse splenocytes vs isotype control (manufacturer)
TCRβ (TCRb, TCR beta chain)	Brilliant UltraViolet 805	300	Ar Ham IgG	H57-597	BD Biosciences	748405	Not validated by manufacturer (clone previously validated)
Siglec-F	Brilliant Violet 421	500	Rat IgG2a, κ	E50-2440	BD Biosciences	562681	Tested on mouse bone marrow vs isotype control (manufacturer)
CD11b	Brilliant Violet 480	600	Rat IgG2b, κ	M1/70	BD Biosciences	566117	Tested on mouse bone marrow vs isotype control (manufacturer)
MHC Class II (I-A/I-E, MHCII, MHC-II)	Brilliant Violet 480	800	Rat IgG2a, κ	M5/114.15.2	BD Biosciences	566086	Tested on mouse splenocytes vs isotype control (manufacturer)
γδTCR (TCRgd, TCR gamma/delta)	Brilliant Violet 480	300	Armenian Hamster IgG2, κ	GL3	BD Biosciences	746343	Not validated by manufacturer (clone previously validated)
CD44	Brilliant Violet 711	400	Rat IgG2b, κ	IM7	BD Biosciences	563971	Tested on mouse bone marrow vs isotype control (manufacturer)
CD90.2 (Thy-1.2)	Brilliant Violet 786	1000	Rat IgG2a, κ	53-2.1	BD Biosciences	564365	Tested on mouse thymocytes vs isotype control (manufacturer)
CD90.1 (Thy-1.1)	FITC	300	Mouse IgG2a, k	HIS51	BD Biosciences	554894	Tested on mouse and rat splenocytes vs isotype control (manufacturer)
cMaf (c-Maf, Maf, mouse/human)	PE	200	Mouse IgG2a, κ	T54-853	BD Biosciences	565795	Tested on human PBMC and mouse PEC vs isotype control (manufacturer)
Siglec-F	PE	800	Rat IgG2a, κ	E50-2440	BD Biosciences	552126	Tested on mouse bone marrow vs isotype control (manufacturer)
RORγt	Brilliant Violet 421	200	Mouse IgG2a, κ	Q31-378	BD Biosciences	562894	Tested on mouse thymocytes vs isotype control (manufacturer)
CCR9	AlexaFluor 647	200	Mouse IgG2a, κ	CW-1.2	BioLegend	128708	Tested on mouse thymocytes vs isotype control (manufacturer)
CD8b.2 (Ly-3.2)	AlexaFluor 700	1000	Rat IgG2b, κ	YTS156.7.7	BioLegend	126618	Tested on mouse splenocytes vs isotype control (manufacturer)
CD90.2	AlexaFluor 700	500	Rat IgG2b, κ	30-H12	BioLegend	105320	Tested on mouse thymocytes vs isotype control (manufacturer)
IgD	AlexaFluor 700	700	Rat IgG2a Kaapa	11-26c.2a	BioLegend	405729	Tested on mouse splenocytes vs isotype control (manufacturer)
TNFa	AlexaFluor 700	500	Rat IgG2a, k	RM4-5	BioLegend	506338	Tested on stimulated mouse splenocytes vs isotype control (manufacturer)
CD64	APC	200	Rat IgG2a, κ	X54-5/7.1	BioLegend	139306	Tested on mouse bone marrow vs isotype control (manufacturer)
CD223 (LAG-3)	APC	200	Rat IgG1, κ	C9B7W	BioLegend	125210	Tested on stimulated mouse splenocytes vs isotype control (manufacturer)
Streptavidin	Brilliant Violet 421	2000	N/A	Streptavidin	BioLegend	405225	NA
CD304 (Nrp-1, Neuropilin-1)	Brilliant Violet 421	400	Rat IgG2a, κ	3E12	BioLegend	145209	Tested on mouse splenocytes vs isotype control (manufacturer)
IL-10 (Il10)	Brilliant Violet 421	400	Rat IgG2b, k	JES5-16E3	BioLegend	505021	Tested on polarized stimulated mouse splenocytes vs isotype control (manufacturer)
IL-5	Brilliant Violet 421	400	Rat IgG2b, κ	17A2	BioLegend	504311	Tested on polarized stimulated mouse splenocytes vs isotype control (manufacturer)
Streptavidin	Brilliant Violet 510	2000	N/A	SA	BioLegend	405233	NA
CD45	Brilliant Violet 510	1000	Rat IgG2b, κ	30-F11	BioLegend	103137	Tested on mouse splenocytes vs isotype control (manufacturer)
CD8a (Ly-2)	Brilliant Violet 510	400	Rat IgG2a, κ	53-6.7	BioLegend	100752	Tested on mouse splenocytes vs isotype control (manufacturer)
CD45.2	Brilliant Violet 510	NA	Mouse (SJL) IgG2a, κ	104	BioLegend	109837	Tested on mouse splenocytes vs isotype control (manufacturer), used for IV labeling
CD45	Brilliant Violet 570	1000	Rat IgG2b, κ	30-F11	BioLegend	103136	Tested on mouse splenocytes vs isotype control (manufacturer)
CD62L	Brilliant Violet 570	100	Rat IgG2a, κ	MEL-14	BioLegend	104433	Tested on mouse splenocytes vs isotype control (manufacturer)
CD3e	Brilliant Violet 605	400	Rat IgG2b, κ	17A2	BioLegend	100237	Tested on mouse splenocytes vs isotype control (manufacturer)
CD4	Brilliant Violet 605	500	Rat IgG2a, k	RM4-5	BioLegend	100548	Tested on mouse splenocytes vs isotype control (manufacturer)
CD73 (ecto-5`-nucleotidase)	Brilliant Violet 605	400	Rat IgG1, κ	TY/11.8	BioLegend	127215	Tested on mouse splenocytes vs isotype control (manufacturer)
Streptavidin	Brilliant Violet 650	2000	N/A	Streptavidin	BioLegend	405232	NA
CD44	Brilliant Violet 650	400	Rat IgG2b, κ	IM7	BioLegend	103049	Tested on mouse splenocytes vs isotype control (manufacturer)
MHCII (I-A/E)	Brilliant Violet 650	1000	Rat IgG2b, κ	M5/114.15.2	BioLegend	107641	Tested on mouse splenocytes vs isotype control (manufacturer)
CD5	Brilliant Violet 711	500	Rat IgG2a, κ	53-7.3	BioLegend	100639	Tested on mouse splenocytes vs isotype control (manufacturer)
CD8	Brilliant Violet 711	500	Rat IgG2a, κ	53-6.7	BioLegend	100759	Tested on mouse splenocytes vs isotype control (manufacturer)
IFN-γ (IFNg, IFN-g, IFNγ)	Brilliant Violet 711	200	Rat IgG1, κ	XMG1.2	BioLegend	505836	Tested on stimulated mouse splenocytes vs isotype control (manufacturer)
Ly-6C (Ly6C)	Brilliant Violet 711	1000	Rat IgG2c, κ	HK1.4	BioLegend	128037	Tested on mouse bone marrow vs isotype control (manufacturer)
CD11b	Brilliant Violet 750	1000	Rat IgG2b, κ	M1/70	BioLegend	101267	Tested on mouse bone marrow vs isotype control (manufacturer)
CD45	Brilliant Violet 750	1000	Rat IgG2b, κ	30-F11	BioLegend	103157	Tested on mouse splenocytes vs isotype control (manufacturer)
CX3CR1	Brilliant Violet 785	200	Mouse IgG2a, k	SA011F11	BioLegend	149029	Tested on mouse splenocytes vs isotype control (manufacturer)
CD127 (IL-7 Receptor alpha, IL-7Ra)	Brilliant Violet 785	200	Rat IgG2a, κ	A7R34	BioLegend	135037	Tested on mouse splenocytes vs isotype control (manufacturer)
CD19	PE-Cy5	400	Rat IgG2a, κ	6D5	BioLegend	115510	Tested on mouse splenocytes vs isotype control (manufacturer)
IL-2	PE-Cy5	500	Rat IgG2b, κ	JES6-5H4	BioLegend	503824	Tested on stimulated mouse splenocytes vs isotype control (manufacturer)
CCR5 (CD195)	PE-Cy7	200	Armenian Hamster IgG	HM-CCR5 (7A4)	BioLegend	107018	Tested on transfected cell line vs isotype control (manufacturer)
FcεRIα	PE-Cy7	200	Ar Ham IgG	MAR1	BioLegend	134318	Tested on mast cell cell line vs isotype control (manufacturer)
IL-10	PE-Cy7	200	Rat IgG2b, κ	JES5-16E3	BioLegend	505026	Tested on stimulated mouse splenocytes vs isotype control (manufacturer)
TCR β chain	PerCP-Cy5.5	400	Ar Ham IgG	H57-597	BioLegend	109227	Tested on mouse splenocytes vs isotype control (manufacturer)
CD4 (RM4-4)	PerCP-Cy5.5	600	Rat IgG2b, κ	RM4-4	BioLegend	116012	Tested on mouse splenocytes vs isotype control (manufacturer)
CD62L	PerCP-Cy5.5	600	Rat IgG2a, κ	MEL-14	BioLegend	104432	Tested on mouse splenocytes vs isotype control (manufacturer)
TCR β chain	PerCP-Cy5.5	400	Ar Ham IgG	H57-597	BioLegend	109227	Tested on mouse splenocytes vs isotype control (manufacturer)
IgD	AlexaFluor 647	1500	Rat IgG2a, κ	11-26c.2a	BioLegend	405708	Tested on mouse splenocytes vs isotype control (manufacturer)
CD8a (CD8α, Ly-2)	AlexaFluor 532	300	Rat IgG2a, κ	53-6.7	ThermoFisher	58-0081-80	Tested on mouse splenocytes vs isotype control (manufacturer)
Gr-1 (Gr1, Ly-6G/C)	AlexaFluor 532	1000	Rat IgG2b, κ	RB6-8C5	ThermoFisher	58-5931-82	Tested on mouse splenocytes vs isotype control (manufacturer)
CD45(LCA, Ly-5)	AlexaFluor 700	500	IgG2b, kappa	30-F11	ThermoFisher	56-0451-82	Tested on mouse splenocytes vs isotype control (manufacturer)
EGR2	APC	200	Rat IgG2a, k	erongr2	ThermoFisher	17-6691-82	Tested on stimulated mouse splenocytes vs isotype control (manufacturer)
Foxp3	APC	400	Rat IgG2a, κ	FJK-16s	ThermoFisher	17-5773-82	Tested on mouse splenocytes vs isotype control (manufacturer)
IL-4	APC	400	Rat IgG1, κ	11B11	ThermoFisher	17-7041-82	Tested on polarized stimulated mouse splenocytes vs isotype control (manufacturer)

Table S1. Antibody usage and validation information, including other flow cytometry reagents (continued)

Antigen	Fluorophore	Dilution (1/x)	Isotype	Clone	Company	Catalog No.	Validation
KLRG1/MAFA	APC	400	Golden Syrian Hamster IgG	2F1	ThermoFisher	17-5893-82	Tested on mouse splenocytes vs isotype control (manufacturer)
CD90.1 (Thy-1.1)	APC	500	Mouse IgG2a, kappa	HIS51	ThermoFisher	17-0900-82	Tested on mouse splenocytes vs isotype control (manufacturer)
Ly-6C (Ly6C)	APC-eFluor 780	500	Rat IgG2c, kappa	HK1.4	ThermoFisher	47-5932-82	Tested on mouse splenocytes vs isotype control (manufacturer)
NK1.1	APC-eFluor 780	400	Mouse IgG2a, κ	PK136	ThermoFisher	47-5941-82	Tested on mouse splenocytes vs isotype control (manufacturer)
TCRβ chain	APC-eFluor 780	400	Ar Ham IgG	H57-597	ThermoFisher	47-5961-82	Tested on mouse splenocytes vs isotype control (manufacturer)
CD11c (Integrin aX, p150/90)	eFluor 450	400	Armenian Hamster IgG	N418	ThermoFisher	48-0114-82	Tested on mouse splenocytes vs isotype control (manufacturer)
CD4 (L3T4)	eFluor 450	500	Rat IgG2a, κ	RM4-5	ThermoFisher	48-0042-82	Tested on mouse splenocytes vs isotype control (manufacturer)
GITR (TNFRSF18, AITR)	eFluor 450	400	Rat IgG2b	DTA-1	ThermoFisher	48-5874-82	Tested on mouse splenocytes vs isotype control (manufacturer)
IL-17a	eFluor 450	500	IgG1, kappa	17B7	ThermoFisher	48-7177-82	Tested on polarized stimulated mouse splenocytes vs isotype control (manufacturer)
Gata-3	eFluor 660	200	IgG2b, kappa	TWAJ	ThermoFisher	50-9966-42	Tested on mouse thymocytes vs isotype control (manufacturer)
FcεRIα	FITC	200	Ar Ham IgG	03/01/22	ThermoFisher	11-5898-82	Tested on mast cell cell line vs isotype control (manufacturer)
Gr-1 (Gr1,Ly-6C/G, Ly-6G/C)	FITC	1000	Rat IgG2b, κ	RB6-8C5	ThermoFisher	11-5931-82	Tested on mouse bone marrow vs isotype control (manufacturer)
Foxp3	PE	400	Rat IgG2a, κ	FJK-16s	ThermoFisher	12-5773-82	Tested on mouse splenocytes vs isotype control (manufacturer)
Gata-3 (Gata3, Human/Mouse)	eFluor 450	200	Rat IgG2b, κ	TWAJ	ThermoFisher	48-9966-42	Tested on mouse thymocytes vs isotype control (manufacturer)
CD69	PE-Cy5	500	Ar Ham IgG	H1.2F3	ThermoFisher	15-0691-82	Tested on stimulated mouse splenocytes vs isotype control (manufacturer)
CD25	PE-Cy5.5	400	Rat IgG1, lambda	PC61.5	ThermoFisher	35-0251-82	Tested on mouse splenocytes vs isotype control (manufacturer)
KLRG1 (MAFA)	PE-Cy5.5	500	Syrian Hamster IgG	2F1	ThermoFisher	35-5893-82	Tested on mouse splenocytes vs isotype control (manufacturer)
Streptavidin	PE-Cy7	1000	NA	Streptavidin	ThermoFisher	25-4317-82	NA
CD127 (IL-7 Receptor alpha, IL-7Ra, IL7ra)	PE-Cy7	200	Rat IgG2a, κ	A7R34	ThermoFisher	25-1271-82	Tested on mouse splenocytes vs isotype control (manufacturer)
ICOS	PE-Cy7	200	Rat IgG2b, κ	7E.17G9	ThermoFisher	25-9942-82	Tested on stimulated mouse splenocytes vs isotype control (manufacturer)
IL-13	PE-Cy7	500	Rat IgG1, Î°	ebio13A	ThermoFisher	25-7133-82	Tested on polarized stimulated mouse splenocytes vs isotype control (manufacturer)
NK-1.1 (NK1.1, NKR-P1C, Ly-55)	PE-Cy7	400	Mouse IgG2a, kappa	PK136	ThermoFisher	25-5941-82	Tested on mouse splenocytes vs isotype control (manufacturer)
T-bet	PE-Cy7	200	Mouse IgG1, κ	4B10	ThermoFisher	25-5825-82	Tested on human PBMCs vs isotype control (manufacturer)
IL-13	PE-eFluor610	800	Rat IgG1, κ	eBio13A	ThermoFisher	61-7133-82	Tested on polarized stimulated mouse splenocytes vs isotype control (manufacturer)
Ki-67	PE-eFluor610	1000	Rat IgG2a, κ	SolA15	ThermoFisher	61-5698-82	Tested on stimulated mouse splenocytes vs isotype control (manufacturer)
Gr-1 (Gr1, Ly-6G/C)	PerCP-Cy5.5	1000	IgG2b, kappa	RB6-8C5	ThermoFisher	45-5931-80	Tested on mouse bone marrow vs isotype control (manufacturer)
CD117 (c-Kit, cKit)	PerCP-eFluor 710	200	Rat IgG2b, κ	2B8	ThermoFisher	46-1171-82	Tested on Lin- mouse bone marrow vs isotype control (manufacturer)
CD39	PerCP-eFluor 710	300	Rat IgG2b, κ	24DmS1	ThermoFisher	46-0391-82	Tested on mouse splenocytes vs isotype control (manufacturer)
IL-22	PerCP-eFluor 710	400	IgG1, kappa	1H8PWSR	ThermoFisher	46-7221-82	Tested on polarized stimulated mouse splenocytes vs isotype control (manufacturer)
γδTCR (TCRgd, TCR gamma/delta)	PerCP-eFluor 710	500	Armenian Hamster IgG2, κ	GL3	ThermoFisher	46-5711-82	Tested on mouse splenocytes vs isotype control (manufacturer)
CD45.2	redfluor710	400	Ms IgG2a, κ	104	Tonbo Bioscience	80-0454-U100	Tested on mouse splenocytes vs isotype control (manufacturer)
CD8a (Ly-2)	APC	400	Rat IgG2a, κ	53-6.7	Tonbo Bioscience	20-0081-U100	Tested on mouse splenocytes vs isotype control (manufacturer)
MHC Class II (I-A/I-E, MHCII, MHC-II)	redfluor710	500	Rat IgG2b, κ	M5/114.15.2	Tonbo Bioscience	80-5321-U100	Tested on mouse splenocytes vs isotype control (manufacturer)
ZombieNIR	Death	1000	NA	NA	BioLegend	423106	Tested on old mouse splenocytes vs isotype control (manufacturer)
CD3ε	None	NA	Armenian Hamster IgG1	145-2C11	BioXCell	BE0001-1	Tested on mouse splenocytes vs isotype control (manufacturer)
CD28	None	NA	Syrian Hamster IgG2	37.51	BioXCell	BE0015-1	Tested on recombinant protein by SDS-PAGE/Western Blot (manufacturer)

Table S2. Simplified Geboes Score rubric

Grade	Grade description	Score	Score Description
0	No inflammatory Activity	0	No abnormalities
		1	Presence of architectural changes
		2	Presence of architectural changes and chronic mononuclear cell infiltrate
1	Basal Plasma cells	0	No increase
		1	Mild increase
		2	Marked increase
2A	Eosinophils In lamina propria	0	No increase
		1	Mild increase
		2	Marked increase
2A	Neutrophils In lamina propria	0	No increase
		1	Mild increase
		2	Marked increase
3	Neutrophils In epithelium	0	None
		1	< 50% crypts involved
		2	> 50% crypts involved
4	Epithelial injury In crypt and Surface Epithelium	0	None
		1	Marked attenuation
		2	Probable crypt destruction: probable erosions
		3	Unequivocal crypt destruction: unequivocal erosion
		4	Ulcer or granulation tissue
