# Supplementary material for: Terminal differentiation and persistence of effector regulatory T cells essential for preventing intestinal inflammation
Source: Nat Immunol. 2025 Feb 4;26(3):444–58. doi: 10.1038/s41590-024-02075-6 (PMC11876075; doi:10.1038/s41590-024-02075-6)
Supplement: Supplementary file 2 — Reporting Summary [file 41590_2024_2075_MOESM2_ESM.pdf]

nature portfolio

Corresponding author(s): Alexander Rudensky

Last updated by author(s): Dec 23, 2024

Reporting Summary

Nature Portfolio wishes to improve the reproducibility of the work that we publish. This form provides structure for consistency and transparency in reporting. For further information on Nature Portfolio policies, see our Editorial Policies and the Editorial Policy Checklist.

Statistics

For all statistical analyses, confirm that the following items are present in the figure legend, table legend, main text, or Methods section.

n/a	Confirmed	
☐	☒	The exact sample size (*n*) for each experimental group/condition, given as a discrete number and unit of measurement
☐	☒	A statement on whether measurements were taken from distinct samples or whether the same sample was measured repeatedly
☐	☒	The statistical test(s) used AND whether they are one- or two-sided<br>*Only common tests should be described solely by name; describe more complex techniques in the Methods section.*
☐	☒	A description of all covariates tested
☐	☒	A description of any assumptions or corrections, such as tests of normality and adjustment for multiple comparisons
☐	☒	A full description of the statistical parameters including central tendency (e.g. means) or other basic estimates (e.g. regression coefficient) AND variation (e.g. standard deviation) or associated estimates of uncertainty (e.g. confidence intervals)
☐	☒	For null hypothesis testing, the test statistic (e.g. *F*, *t*, *r*) with confidence intervals, effect sizes, degrees of freedom and *P* value noted<br>*Give P values as exact values whenever suitable.*
☒	☐	For Bayesian analysis, information on the choice of priors and Markov chain Monte Carlo settings
☒	☐	For hierarchical and complex designs, identification of the appropriate level for tests and full reporting of outcomes
☐	☒	Estimates of effect sizes (e.g. Cohen's *d*, Pearson's *r*), indicating how they were calculated

Our web collection on statistics for biologists contains articles on many of the points above.

Software and code

Policy information about availability of computer code

Data collection	The software used for data collection and their versions are explicitly stated in the relevant portions of the Methods.
Data analysis	The software used for data analysis and their versions are explicitly stated in the relevant portions of the Methods. Availability of data and code are described in the appropriate sections:<br>Bulk RNA-seq, ATAC-seq, and single cell RNA-seq / TCR-seq data is available from the GEO database (GSE207969).<br>All custom code used in analysis of data and generation of plots is available upon request (sdikiy@scripps.edu).

For manuscripts utilizing custom algorithms or software that are central to the research but not yet described in published literature, software must be made available to editors and reviewers. We strongly encourage code deposition in a community repository (e.g. GitHub). See the Nature Portfolio guidelines for submitting code & software for further information.

Data

Policy information about availability of data

All manuscripts must include a data availability statement. This statement should provide the following information, where applicable:

- Accession codes, unique identifiers, or web links for publicly available datasets
- A description of any restrictions on data availability
- For clinical datasets or third party data, please ensure that the statement adheres to our policy

All sequencing data (Bulk RNA-seq, ATAC-seq, and single cell RNA-seq) genrated for this study are available from NCBI GEO database (GSE207969). Previously published datasets are appropriately referenced in the main text and methods sections.

Research involving human participants, their data, or biological material

Policy information about studies with human participants or human data. See also policy information about sex, gender (identity/presentation), and sexual orientation and race, ethnicity and racism.

Reporting on sex and gender	NA
Reporting on race, ethnicity, or other socially relevant groupings	NA
Population characteristics	NA
Recruitment	NA
Ethics oversight	NA

Note that full information on the approval of the study protocol must also be provided in the manuscript.

Field-specific reporting

Please select the one below that is the best fit for your research. If you are not sure, read the appropriate sections before making your selection.

☒ Life sciences ☐ Behavioural & social sciences ☐ Ecological, evolutionary & environmental sciences

For a reference copy of the document with all sections, see nature.com/documents/nr-reporting-summary-flat.pdf

Life sciences study design

All studies must disclose on these points even when the disclosure is negative.

Sample size	No tests were performed to determine appropriate sample size. Sample sizes for experiments were chosen based on previous experience and feasibility (6-12 mice in a single experiment). Post hoc power calculations demonstrate that experiments were not under-powered.
Data exclusions	No data were excluded. In RNA-seq analysis, certain low expressed genes were excluded. The rationale is described in the Methods.
Replication	Almost all 'wet' experiments were repeated at least twice, most thrice, as described in Figure legends. Two experiments (depicted in Extended Data Figures 4 and 10) were performed once. Most data are presented as pooled across multiple repeated experiments.
Randomization	For experiments without genetic controls, mice were randomly allocated to groups within each sex and litter. Sex and litter were intentionally balanced in different groups in all experiments.
Blinding	Researchers were not blind during experimentation and data acquisition, as the person performing the experiment was the person designing them. As data were for the most part strictly quantitative, this was deemed acceptable.

Reporting for specific materials, systems and methods

We require information from authors about some types of materials, experimental systems and methods used in many studies. Here, indicate whether each material, system or method listed is relevant to your study. If you are not sure if a list item applies to your research, read the appropriate section before selecting a response.

Materials & experimental systems

n/a	Involved in the study	
☐	☒	Antibodies
☐	☒	Eukaryotic cell lines
☒	☐	Palaeontology and archaeology
☐	☒	Animals and other organisms
☒	☐	Clinical data
☒	☐	Dual use research of concern
☒	☐	Plants

Methods

n/a	Involved in the study	
☒	☐	ChIP-seq
☐	☒	Flow cytometry
☒	☐	MRI-based neuroimaging

Antibodies

Antibodies used: This information is provided in Table S1

Validation: This information is provided in Table S1

Eukaryotic cell lines

Policy information about cell lines and Sex and Gender in Research

Cell line source(s): 293T cells from ATCC (cat CRL-3216)

Authentication: Cell lines were not authenticated as they are not a known commonly misidentified cell line.

Mycoplasma contamination: Cells were not tested for mycoplasma contamination

Commonly misidentified lines (See ICLAC register): None were used

Animals and other research organisms

Policy information about studies involving animals; ARRIVE guidelines recommended for reporting animal research, and Sex and Gender in Research

Laboratory animals: All mouse strains used were on the C57BL/6 background. Specific strains are described and referenced where appropriate in the Methods. Age and sex are explicitly stated in all figure legends.

Wild animals: No wild animals were used in the study

Reporting on sex: In most cases data for both sexes of mice were collected and reported. Deviations from this are explictly described and explained in relevant figure legends and methods.

Field-collected samples: No field collected samples were used in the study

Ethics oversight: The MSKCC IACUC approved all studies under protocol 08-10-023.

Note that full information on the approval of the study protocol must also be provided in the manuscript.

Plants

Seed stocks: *Report on the source of all seed stocks or other plant material used. If applicable, state the seed stock centre and catalogue number. If plant specimens were collected from the field, describe the collection location, date and sampling procedures.*

Novel plant genotypes: *Describe the methods by which all novel plant genotypes were produced. This includes those generated by transgenic approaches, gene editing, chemical/radiation-based mutagenesis and hybridization. For transgenic lines, describe the transformation method, the number of independent lines analyzed and the generation upon which experiments were performed. For gene-edited lines, describe the editor used, the endogenous sequence targeted for editing, the targeting guide RNA sequence (if applicable) and how the editor was applied.*

Authentication: *Describe any authentication procedures for each seed stock used or novel genotype generated. Describe any experiments used to assess the effect of a mutation and, where applicable, how potential secondary effects (e.g. second site T-DNA insertions, mosiacism, off-target gene editing) were examined.*

Flow Cytometry

Plots

Confirm that:

☒ The axis labels state the marker and fluorochrome used (e.g. CD4-FITC).

☒ The axis scales are clearly visible. Include numbers along axes only for bottom left plot of group (a 'group' is an analysis of identical markers).

☒ All plots are contour plots with outliers or pseudocolor plots.

☒ A numerical value for number of cells or percentage (with statistics) is provided.

Methodology

Sample preparation	Described in detail in the Methods.
Instrument	Cytek Aurora 5 laser used for analysis; BD Aria II used for sorting.
Software	Cytek SpectroFlo (multiple versions) and BD FACSDiva (multiple versions) used for acquistion; FlowJo v10.X used for analysis.
Cell population abundance	For sorted populations, all samples were determined to be >95% pure by a post-sort purity check on the sorted sample.
Gating strategy	Gating strategies are demonstrated in supplementary figures 1-10.

☒ Tick this box to confirm that a figure exemplifying the gating strategy is provided in the Supplementary Information.
